# In Vitro Anticancer and Cancer-Preventive Activity of New Triterpene Glycosides from the Far Eastern Starfish *Solaster pacificus*

**DOI:** 10.3390/md20030216

**Published:** 2022-03-20

**Authors:** Timofey V. Malyarenko, Olesya S. Malyarenko, Alla A. Kicha, Anatoly I. Kalinovsky, Pavel S. Dmitrenok, Natalia V. Ivanchina

**Affiliations:** 1 G.B. Elyakov Pacific Institute of Bioorganic Chemistry, Far Eastern Branch of the Russian Academy of Sciences, Pr. 100-Let Vladivostoku 159, 690022 Vladivostok, Russia; malyarenko.os@gmail.com (O.S.M.); kicha@piboc.dvo.ru (A.A.K.); kaaniw@piboc.dvo.ru (A.I.K.); paveldmt@piboc.dvo.ru (P.S.D.); 2 Department of Bioorganic Chemistry and Biotechnology, School of Natural Sciences, Far Eastern Federal University, Russky Island, Ajax Bay, 10, 690922 Vladivostok, Russia

**Keywords:** triterpene glycosides, starfish, *Solaster pacificus*, neoplastic cell transformation, EGF, TPA, X-ray, UVB, cancer prevention, anticancer activity

## Abstract

Sea stars or starfish (class Asteroidea) and holothurians or sea cucumbers (class Holothuroidea), belonging to the phylum Echinodermata (echinoderms), are characterized by different sets of glycosidic metabolites: the steroid type in starfish and the triterpene type in holothurians. However, herein we report the isolation of eight new triterpene glycosides, pacificusosides D–K (**1**–**3, 5**–**9**) along with the known cucumarioside D (**4**), from the alcoholic extract of the Far Eastern starfish *Solaster pacificus*. The isolated new compounds are closely related to the metabolites of sea cucumbers, and their structures of **1**–**3** and **5**–**9** were determined by extensive NMR and ESIMS techniques. Compounds **2**, **5**, and **8** have a new type of tetrasaccharide chain with a terminal non-methylated monosaccharide unit. Compounds **3**, **6**, and **9** contain another new type of tetrasaccharide chain, having 6-*O*-SO_3_-Glc as one of the sugar units. The cytotoxic activity of **1**–**9** against non-cancerous mouse epidermal cells JB6 Cl41 and human melanoma cell lines SK-MEL-2, SK-MEL-28, and RPMI-7951 was determined by MTS assay. Compounds **1**, **3**, **4**, **6**, and **9** showed potent cytotoxicity against these cell lines, but the cancer selectivity (SI > 9) was observed only against the SK-MEL-2 cell line. Compounds **1**, **3**, **4**, **6**, and **9** at the non-toxic concentration of 0.1 μM significantly inhibited neoplastic cell transformation of JB6 Cl41 cells induced by chemical carcinogens (EGF, TPA) or ionizing radiation (X-rays and UVB). Moreover, compounds **1** and **4** at the non-toxic concentration of 0.1 µM possessed the highest inhibiting activity on colony formation among the investigated compounds and decreased the colonies number of SK-MEL-2 cells by 64% and 70%, respectively. Thus, triterpene glycosides **1** and **4** can be considered as prospective cancer-preventive and anticancer-compound leaders.

## 1. Introduction

Carcinogenesis is a multiphase and complex process characterized by uncontrolled growth and spread of atypical cells and representing one of the most burdensome problems, both from a medical and socio-economic point of view. Despite the significant advances in the field of surgical oncology, radiation therapy, and oncopharmacology, cancer ranks second in the structure of mortality in most countries of the world [1,2]. The majority of human cancers’ cases are caused by environmental and lifestyle factors. The etiology of all cancers is associated with inherited genetic aberrations (5–10%) and acquired genetic abnormality (90–95%) caused by exogenous and/or endogenous environmental agents [3]. A number of epidemiological studies have successfully shown that exposures to certain environmental effects (exogenous/societal, occupational, lifestyle, industrial, agricultural, etc.), physical (UV/solar, ionizing radiations) and biological agents (viruses such as Epstein–Barr (EBV), hepatitis B (HBV), hepatitis C (HCV), human papilloma (HPV), and human immunodeficiency virus type 1 (HIV-1)), bacteria such as *Helicobacter* *pylori*, and parasites such as liver fluke increase the cancer risk [4,5]. Cancer prevention is one of the approaches in the fight against this disease. The idea of cancer chemoprevention was introduced by Sporn and Wattenberg [6,7]. Chemoprevention is one of the anticancer approaches wherein natural/synthetic agent(s) are prescribed with the aim to delay or disrupt multiple pathways and processes involved at multiple steps: the initiation, promotion, and progression of cancer [4]. Natural compounds from terrestrial plants (resveratrol, (–)-epigallocatechin gallate (EGCG) [6], and others) or derived from marine organisms, for example, polysaccharides from brown seaweeds, have been reported to possess cancer-preventive effects via the modulation of various molecular signal transduction pathways [8]. The further search for new natural products with cancer-preventive activity continues to be an important mission.

The phylum Echinodermata is one of the main sources of marine natural compounds [9]. Moreover, classes Holothuroidea (sea cucumbers) and Asteroidea (starfish) are the highest sources of glycosylated compounds, which possess a wide spectrum of biological activity: cytotoxic [10,11], antifungal [12,13], bactericidal, hemolytic, antiviral, antiparasitic [14], antibiofouling, neuritogenic, anti-inflammatory [15,16,17,18,19,20,21,22,23], and immunomodulatory [24]. It is also worth noting that triterpene glycosides are typical secondary metabolites of sea cucumbers [25], while polar steroids and their glycosides (glycosides of polyhydroxysteroids, cyclic glycosides, and asterosaponins) are typical secondary metabolites of starfish [15,16,17,18,19,20,21,22,23]. Only two new triterpene glycosides, rollentosides A and B, were earlier isolated from the starfish *Asterias rollestoni* [26], and one previously known triterpene glycoside, holothurin A2 (=echinoside A), was isolated from the starfish *Choriaster granulatus* [27]. In addition, very recently, we reported about the isolation and structure elucidation of three new triterpene glycosides, pacificusosides A–C, and three previously known triterpene glycosides, cucumariosides C_1_, C_2_, and A_10_, from the Far Eastern starfish *Solaster pacificus*. We supposed that these compounds were obtained with diet from sea cucumbers belonging to the genus *Eupentacta* and partly metabolized in the starfish [28]. We have also described the cytotoxicity of isolated triterpene glycosides against human embryonic kidney HEK 293 cells, colorectal carcinoma HT-29 cells, melanoma RPMI-7951 cells, and breast cancer MDA-MB-231 cells using MTS assay. Among the isolated compounds, cucumariosides C_1_, C_2_, and A_10_ demonstrated the highest cytotoxic activity against the tested cell lines. At the same time, the cytotoxic effects of the investigated triterpene glycosides were reduced by cholesterol, such as with the similar effects of the previously investigated triterpene glycosides. Finally, pacificusoside C and cucumariosides C_1_ and C_2_ almost completely suppressed the colony formation of the HT-29, RPMI-7951, and MDA-MB-231 cells at a non-toxic concentration of 0.5 μM [28].

Continuing our research on the active secondary metabolites of the starfish *Solaster pacificus* (order Valvatida, family Solasteridae), collected in the Sea of Okhotsk near Iturup Island, we report herein the results of studies on isolation and structures of series of new triterpene glycosides, pacificusosides D–K (**1**–**3**, **5**–**9**), and one previously known triterpene glycoside, cucumarioside D (**4**). Moreover, our research was focused on the cancer-preventive and anticancer activities of the isolated triterpene glycosides. In particular, the obtained results revealed the potent inhibiting effect of these compounds on the EGF-, TPA-, X-ray-, and UVB-induced neoplastic cell transformation of non-cancerous cells and the colony formation of human melanoma cells.

## 2. Results and Discussion

### 2.1. The Isolation and Structure Elucidation of Compounds 1–9 from S. pacificus

**Figure 1:**
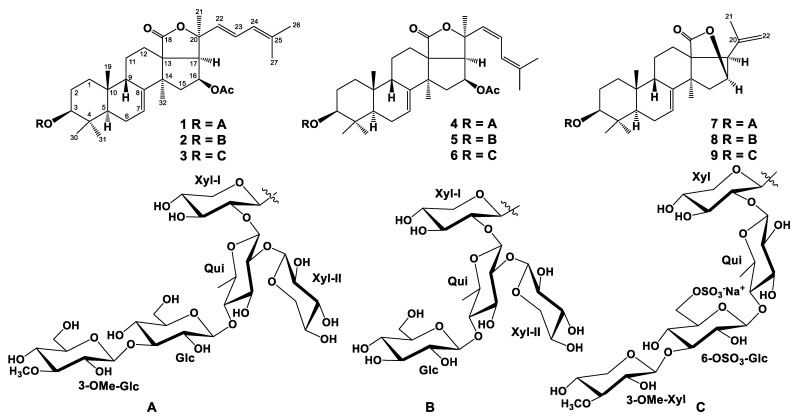
The structures of compounds **1**−**9** isolated from *S. pacificus*.

The concentrated ethanol extract of the starfish *S. pacificus* was subjected to sequential separation by chromatography on columns with Polychrome 1 and Si gel, followed by HPLC on semi-preparative Diasorb-130-C16T, Diasfer-110-C18, and Discovery C18 columns, to yield eight new triterpene glycosides, named as pacificusosides D–K (**1**–**3**, **5**–**9**), and one previously known triterpene glycoside (**4**) (Figure 1). Compound **4** was identified by comparison of its ^1^H, ^13^C NMR, and MS spectra with those reported for cucumarioside D—a minor triterpene glycoside from the sea cucumber *Eupentacta fraudatrix* [29].

**Figure 2:**
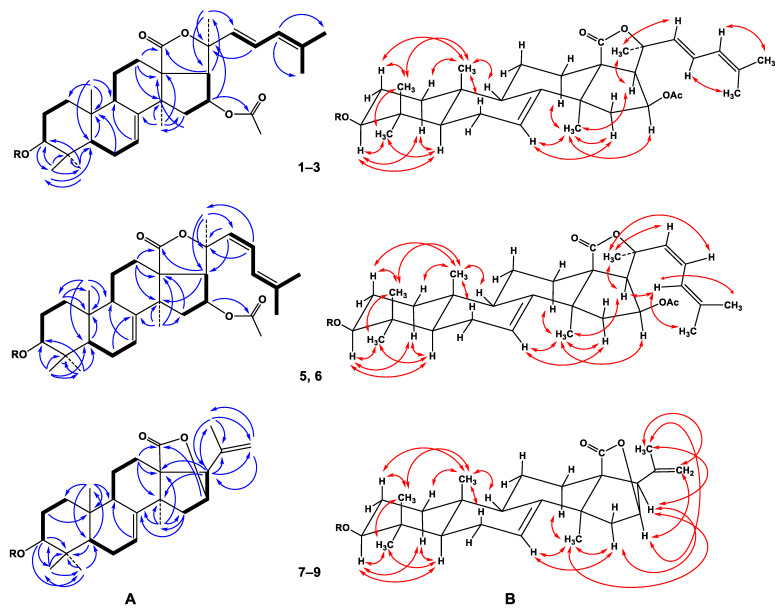
(**A**) ^1^H-^1^H COSY and key HMBC correlations for compounds **1**–**3** and **5**–**9**. (**B**) Key ROESY correlations for compounds **1**–**3** and **5**–**9**.

The molecular formula of **1** was determined to be C_61_H_94_O_27_ from the [M+Na]^+^ sodium adduct ion peak at *m/z* 1281.5879 (calcd for [C_61_H_94_O_27_Na]^+^, 1281.5875) in the (+)HRESIMS spectrum (Figure S1). The IR spectrum of compound **1** showed the presence of hydroxy (3418 cm^−1^), γ-lactone (1735 cm^−1^), and olefinic (1622 cm^−1^) groups (Figure S2). The ^1^H and ^13^C NMR spectroscopic data belonging to polycyclic moiety of **1** showed the resonances of protons and carbons of four methyl groups (*δ*_H_ 1.24 s, 1.14 s, 1.34 s, 1.18 s; *δ*_C_ 23.9, 17.2, 28.6, 32.1), the 7(8)-double bond (*δ*_H_ 5.71 m; *δ*_C_ 120.5, 145.5), OAc group (*δ*_H_ 1.98 s, *δ*_C_ 21.2, 170.3), and lactone carbonyl (*δ*_C_ 179.2) (Table 1, Figures S3–S10). The ^1^H-^1^H COSY and HSQC correlations attributable to the terpene nucleus revealed the corresponding sequences of protons from C-1 to C-3, C-5 to C-7, C-9 to C-12 through C-11, and from C-15 to C-17 (Figure 2A, Figures S11 and S12). The key HMBC cross-peaks, such as H-3/C-30; H-5/C-10; H-6/C-10; H-7/C-9; H-12/C-13, C-14, C-18; H-15/C-13, C-32; H-16/C-20, CO(OAc); H-17/C-18, C-21; H_3_-19/C-1, C-5, C-9, C-10; H_3_-30/C-3, C-4, C-5, C-31; H_3_-31/C-3, C-4, C-5, C-30; H_3_-32/C-8, C-13, C-14, C-15, confirmed the overall structure of the pentacyclic terpene moiety of **1** (Figure 2A and Figure S13). The key ROESY cross-peaks showed the common 5α/9β/10β/13β,14α stereochemistry of the terpene nucleus and 3β,16β-configurations of the oxygenated substituents in **1** (Figure 2B and Figure S14).

The proton and carbon signals of the aglycon side chain of **1** demonstrated the presence of three methyl groups (*δ*_H_ 1.57 s, 1.61 s, 1.69 s; *δ*_C_ 30.2, 17.9, 25.6) and the conjugated 22,24-diene system (*δ*_H_ 5.92 d (*J* = 15.7), 6.56 dd (*J* = 15.7, 11.0), 5.86 brd (11.0); *δ*_C_ 134.0, 122.4, 125.2, 134.6). The observation of a UV absorption maximum at λ_max_ = 240 nm was consistent with this assignment [30,31] (Figure S15). The protons sequence from H-22 to H-27 correlated with the corresponding carbon atoms of the side chain of **1** was assigned using the COSY and HSQC experiments (Table 1 and Table 2, Figures S11 and S12). The key HMBC H_3_-21/C-17, C-20, C-22; H-22/C-20 and ROESY correlations H_3_-21/H-17, H-20, H-22; H-23/H-20, H_3_-27; and H-24/H_3_-26 supported the total structure of the Δ^22,24^-cholestane side chain (Figure 1, Figures S13 and S14). The *trans* configuration of the 22(23)-double bond followed from *J*_22,23_ = 15.7 Hz. The NMR spectroscopic data of the aglycon part of **1** were coincident with those of the known cucumarioside C_2_ from *E. fraudatrix*, with holostane-type aglycon having 16β-OAc and 7(8)-double bonds in the nucleus and Δ^22,24^-lanostane side chain [32].

In addition to the above-mentioned signals, the ^1^H NMR spectrum of **1** exhibited five resonances in the deshielded region due to the anomeric protons of monosaccharide units at *δ*_H_ 4.87, 5.20, 4.92, 5.27, and 5.39 that correlated in the HSQC experiment with a carbon signal at *δ*_C_ 105.1, 103.0, 104.7, 105.5, and 105.8, respectively, as well as a resonance due to an *O*-methyl group at *δ*_H_ 3.87 that correlated in the HSQC experiment with a carbon signal at *δ*_C_ 60.5 (Table 2, Figures S3–S10).

The sequence of monosaccharide units in the carbohydrate chain of **1** was confirmed by ESIMS/MS data. In fact, the (−)ESIMS/MS spectrum of the molecular anion peak [M − H]^–^ at *m/z* 1257 showed fragmentary peaks obtained due to the losses of sugar units at *m/z* 1125 [(M − H)−132]^−^, loss of pentose; 1081 [(M – H) –176]^–^, loss of *O*-Me-hexose; 949 [(M − H) – 176 − 132]^–^, losses of *O*-Me-hexose and pentose; 919 [(M – H) – 176 – 162]^–^, losses of *O*-Me-hexose and hexose; 787 [(M – H) – 176 – 132 – 162]^–^, losses of *O*-Me-hexose, hexose, and pentose; 641 [(M − H) – 176 – 132 – 162 – 146]^–^, and losses of *O*-Me-hexose, hexose, pentose, and 6-deoxyhexose.

**Figure 3:**
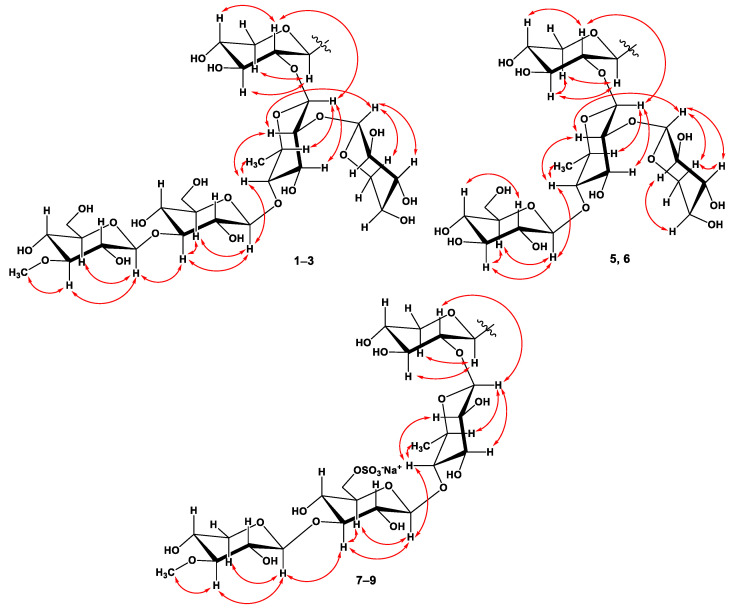
Key ROESY correlations of oligosaccharide moiety for compounds **1**–**3** and **5**–**9**.

Along with mass spectral information, these data showed the presence of five monosaccharide residues in the oligosaccharide moiety of **1**. The existence of a 6-deoxy-sugar unit was supported by one methyl doublet at *δ*_H_ 1.70. The coupling constants (7.0–8.0 Hz) of anomeric protons were indicative of a β-configuration of all the glycosidic bonds. The chemical shifts and coupling constants of H-1–H-5 or H-6 of monosaccharide units were determined by irradiation of anomeric protons in the 1D TOCSY experiments. ^1^H-^1^H COSY, HSQC, HMBC, and ROESY experiments led to the assignment of all the proton and carbon signals of the carbohydrate chain of **1** (Table 2, Figure 3, and Figures S11–S14).

The spectroscopic data of the oligosaccharide moiety strictly coincided with those of terminal 3-*O*-methyl-β-d-glucopyranosyl and β-d-xylopyranosyl residues and internal 3-*O*-substituted β-d-glucopyranosyl, 2,4-di-*O*-substituted β-d-quinovopyranosyl, and 2-*O*-substituted β-d-xylopyranosyl residues in the earlier reported ^1^H and ^13^C NMR spectra of the known cucumarioside D [29].

Respectively, the (+)ESIMS/MS spectrum of the sodiated molecular ion peak [M + Na]^+^ at *m/z* 1281 of **1** showed a series of fragmentary peaks at *m/z* 1221 [(M + Na) – 60]^+^, loss of OAc group; 1149 [(M + Na) – 132]^+^, loss of pentose; 1105 [(M + Na) – 176]^+^, loss of *O*-Me-hexose; 973 [(M + Na) – 176–132]^+^, losses of *O*-Me-hexose and pentose; 811 [(M + Na) – 176 – 132 – 162]^+^, losses of *O*-Me-hexose, pentose, and hexose; 789 [carbohydrate chain+Na]^+^; 639 [(carbohydrate chain+Na)–132–H_2_O]^+^, losses of pentose and H_2_O from carbohydrate chain; 507 [(carbohydrate chain + Na) – 132 – H2O – 132]^+^, losses of two pentoses and H_2_O from the carbohydrate chain; 361 [(carbohydrate chain + Na) – 132 – H_2_O – 132 – 146]^+^ losses of two pentoses, 6-deoxyhexose and H_2_O from carbohydrate chain; 217 [*O*MeGlc + Na]^+^; 185 [Glc + Na]^+^. The attachment of the oligosaccharide chain to the aglycon and positions of the interglycosidic linkages were deduced from long-range correlations in the HMBC and ROESY spectra (Table 2, Figures S13 and S14).

There were the cross-peaks between H-1 of Xyl*_p_*-I and C-3 (H-3) of aglycon; H-1 of Qui*_p_* and C-2 (H-2) of Xyl*_p_*-I; H-1 of Glc*_p_* and C-4 (H-4) of Qui*_p_*; H-1 of 3-*O*Me-Glc*_p_* and C-3 (H-3) of Glc*_p_*; H-1 of Xyl*_p_*-II and C-2 (H-2) of Qui*_p_*. Acid hydrolysis of glycoside 1 with 2 M TFA was carried out to ascertain its monosaccharide composition. Alcoholysis of the mixture of sugars by (*R*)-(−)-octanol followed by acetylation, GC analysis, and comparison with the corresponding derivatives of standard monosaccharides allowed us to establish the D-configuration for all the monosaccharide units comprising the carbohydrate moiety of 1 (D-xylose, D-quinovose, D-glucose, and 3-*O*-methyl-d-glucose). On the basis of all the above-mentioned data, the structure of pacificusoside A (1) was elucidated to be 3β-*O*-{3-*O*-methyl-β-d-glucopyranosyl-(1→3)-β-d-glucopyranosyl-(1→4)-[β-d-xylopyranosyl-(1→2)]-β-d-quinovopyranosyl-(1→2)-β-d-xylopyranosyl}-16β-acetoxyholosta-7,22*E*,24-triene.

The molecular formula of **2** was determined to be C_54_H_82_O_22_ from the [M + Na]^+^ sodium adduct ion peak at *m/z* 1105.5183 (calculated for [C_54_H_82_O_22_Na]^+^, 1105.5190) in the (+)HRESIMS spectrum (Figure S16). The IR spectrum of compound **2** showed the presence of hydroxy (3414 cm^–1^), γ-lactone (1736 cm^–1^), and olefinic (1619 cm^–1^) groups (Figure S17). The comparison of the ^1^H, ^13^C NMR spectra and application of extensive 2D NMR analysis of compounds **1**, **2**, and **3** exhibited that the triterpene aglycon of **1** is identical to that in compounds **2** and **3**, while compounds **1**, **2**, and **3** differ from each other in oligosaccharide moieties only (Table 1 and Table 2).

The ^1^H NMR spectrum of **2** exhibited four resonances in the deshielded region due to anomeric protons of monosaccharide units at *δ*_H_ 4.87, 5.21, 4.96, and 5.39 that correlated in the HSQC experiment with carbon signals at *δ*_C_ 105.1, 103.0, 105.3, and 105.9, respectively. Along with mass-spectra information, these data showed the presence of four monosaccharide residues in the oligosaccharide moiety of **2**. The existence of a 6-deoxy-sugar unit was supported by one methyl doublet at *δ*_H_ 1.71. The coupling constants (6.9–8.0 Hz) of the anomeric protons were indicative of the β-configurations of all glycosidic bonds. The chemical shifts and coupling constants of H-1–H-5, or H-6 of monosaccharide units, were determined by the irradiation of anomeric protons in the 1D TOCSY experiments. ^1^H-^1^H COSY, HSQC, HMBC, and ROESY experiments led to the assignment of all the proton and carbon signals of the carbohydrate chain of **2** (Figure 3 and Figures S18–S23). The ^1^H and ^13^C NMR spectroscopic data of the oligosaccharide moiety of **2** strictly coincided with those of terminal β-d-glucopyranosyl and β-d-xylopyranosyl residues and the internal 2,4-di-*O*-substituted β-d-quinovopyranosyl and 2-*O*-substituted β-d-xylopyranosyl residues of **1**.

The sequence of monosaccharide units in the carbohydrate chain of **2** was confirmed by the ESIMS/MS data. In fact, the (–)ESIMS/MS spectrum of the molecular anion peak [M – H]^–^ at *m/z* 1081 showed fragmentary peaks obtained due to the losses of sugar units at *m/z* 949 [(M – H) – 132]^–^, the loss of pentose; 919 [(M – H) – 162]^–^, loss of hexose; 787 [(M – H) – 162 – 132]^–^, losses of hexose and pentose; 641 [(M – H) – 162 – 132 – 146]^–^, losses of hexose, pentose, and 6-deoxyhexose. Respectively, the (+)ESIMS/MS spectrum of the sodiated molecular ion peak [M + Na]^+^ at *m/z* 1105 showed a series of fragmentary peaks at *m/z* 1045 [(M + Na) – 60]^+^, the loss of OAc group; 973 [(M + Na) – 132]^+^, loss of pentose; 943 [(M + Na) – 162]^+^, loss of hexose; 811 [(M + Na) – 162 – 132]^+^, losses of hexose and pentose; 613 [carbohydrate chain+Na]^+^; 463 [(carbohydrate chain + Na) – 132 – H_2_O]^+^, losses of pentose and H_2_O from carbohydrate chain; 331 [(carbohydrate chain + Na)–2 × 132 – H_2_O]^+^, losses of two pentoses and H_2_O from the carbohydrate chain; 185 [Glc + Na]^+^, losses of two pentoses, 6-deoxyhexose, and H_2_O from the carbohydrate chain. The attachment of the oligosaccharide chain to the aglycon and positions of the interglycosidic linkages were deduced from long-range correlations in the HMBC and ROESY spectra (Table 2, Figures S22 and S23). There were the cross-peaks between H-1 of Xyl*_p_*-I and C-3 (H-3) of aglycon; H-1 of Qui*_p_* and C-2 (H-2) of Xyl*_p_*-I; H-1 of Glc*_p_* and C-4 (H-4) of Qui*_p_*; H-1 of Xyl*_p_*-II and C-2 (H-2) of Qui*_p_*. The D-series of monosaccharide units was expected by analogy with co-occurring glycoside **1**. Accordingly, the structure of pacificusoside E (**2**) was determined as 3β-*O*-{β-d-glucopyranosyl-(1→4)-[β-d-xylopyranosyl-(1→2)]-β-d-quinovopyranosyl-(1→2)-β-D-xylopyranosyl}-16β-acetoxyholosta-7,22*E*,24-triene. To the best of our knowledge, compound **2** has a novel type of oligosaccharide chain containing non-methylated terminal monosaccharide units never found before in the sea cucumber triterpene glycosides.

The molecular formula of **3** was determined to be C_55_H_83_O_25_SNa from the [M – Na]^–^ ion peak at *m/z* 1175.4944 (calcd for [C_55_H_83_O_25_S]^–^, 1175.4950) in the (–)HRESIMS spectrum (Figure S24). The IR spectrum of compound **3** showed the presence of hydroxy (3415 cm^–1^), γ-lactone (1742 cm^–1^), olefinic (1619 cm^–1^), and sulfate (1243, 817 cm^–1^) groups (Figure S25). The ^1^H NMR spectrum of **3** exhibited four resonances in the deshielded region due to the anomeric protons of monosaccharide units at *δ*_H_ 4.76, 5.06, 4.85, and 5.21 that correlated in the HSQC experiment with a carbon signal at *δ*_C_ 105.5, 104.9 × 2, and 105.9, respectively, as well as one resonance due to an *O*-methyl group at *δ*_H_ 3.85 that correlated in the HSQC experiment with a carbon signal at *δ*_C_ 60.4 (Table 2, Figures S26 and S27). Along with mass-spectrometric information, these data showed the presence of four monosaccharide residues in the oligosaccharide moiety of **3**. The existence of a 6-deoxy-sugar unit was supported by one methyl doublet at *δ*_H_ 1.69. The coupling constants (7.3–8.1 Hz) of the anomeric protons were indicative of a β-configuration of all the glycosidic bonds. The chemical shifts and coupling constants of H-1–H-5, or H-6 of monosaccharide units, were determined by irradiation of the anomeric protons in the 1D TOCSY experiments. ^1^H-^1^H COSY, HSQC, HMBC, and ROESY experiments led to the assignment of all the proton and carbon signals of the carbohydrate chain of **3** (Figure 3, Figures S28–S31). The examination of the NMR spectroscopic data of compounds **3** and **1** exhibited that the signals of oligosaccharide moiety of **3** strictly coincided with those of the terminal 3-*O*Me-β-d-xylopyranosyl residue and internal 4-*O*-substituted β-d-quinovopyranosyl and 2-*O*-substituted β-d-xylopyranosyl residues. The signals of H-6, H′-6 at *δ*_H_ 5.25, 4.82, and C-6 at *δ*_C_ 67.6 of the internal 3-*O*-substituted-β-d-glucopyranosyl residue in the ^1^H and ^13^C NMR spectra of **3** were deshielded in comparison with the same signals at *δ*_H_ 4.45, 4.18, and *δ*_C_ 61.9 in the ^1^H and ^13^C NMR spectra of **1**, respectively, that revealed the presence of a sulfoxy group at the C-6 position in this monosaccharide unit of **3**.

The sequence of monosaccharide units in its carbohydrate chain was confirmed by ESIMS/MS data. In fact, the (–)ESIMS/MS spectrum of the molecular anion peak [M – Na]^–^ at *m/z* 1175 showed fragmentary peaks obtained due to the losses of sugar units at *m/z* 1131 [(M – Na) – 44]^–^, loss of CO_2_; 1115 [(M – Na) – 60]^–^, loss HOAc; 1071 [(M – Na) – 44 – 60]^–^, losses of CO_2_ and HOAc; 1029 [(M – Na) – 146]^–^, loss of *O*-Me-pentose; 665 [carbohydrate chain – Na]^–^; 533 [(carbohydrate chain – Na) – 132]^–^ loss of pentose; 387 [(carbohydrate chain – Na) – 132 – 146]^–^ losses of pentose and *O*-Me-pentose or 6-deoxyhexose; 241 [(carbohydrate chain – Na) – 132 – 146 – 146]^–^ losses of pentose, *O*-Me-pentose, and 6-deoxyhexose. Respectively, the (+)ESIMS/MS spectrum of the sodiated molecular ion peak [M + Na]^+^ at *m/z* 1221 showed a series of fragmentary peaks at *m/z* 1161 [(M + Na) – 60]^+^, loss of acetic acid; 1177 [(M + Na) – 44]^+^, loss of CO_2_; 1075 [(M + Na) – 146]^+^, loss of *O*-Me-pentose; 1015 [(M + Na) – 146 – 60]^+^, losses of *O*-Me-pentose and HOAc; 971 [(M + Na) – 146 – 60 – 44]^+^, losses of *O*-Me-pentose, HOAc, and CO_2_; 711 [carbohydrate chain + Na]^+^; 579 [(carbohydrate chain + Na) – 132]^+^ loss of pentose from the carbohydrate chain; 433 [(carbohydrate chain + Na) – 132 – 146]^+^ losses of pentose and *O*-Me-pentose or 6-deoxyhexose from the carbohydrate chain; 287 [(carbohydrate chain + Na) – 132 – 146 – 146]^+^ losses of pentose, *O*-Me-pentose, and 6-deoxyhexose from the carbohydrate chain. The attachment of the oligosaccharide chain to the aglycon and positions of interglycosidic linkages were deduced from long-range correlations in the HMBC and ROESY spectra (Table 2, Figures S30 and S31). There were the cross-peaks between H-1 of Xyl*_p_* and C-3 (H-3) of aglycon; H-1 of Qui*_p_* and C-2 (H-2) of Xyl*_p_*; H-1 of 6-OSO_3_-Glc*_p_* and C-4 (H-4) of Qui*_p_*; H-1 of 3-OMe-Xyl*_p_* and C-3 (H-3) of 6-OSO_3_-Glc*_p_*. The D-configurations of monosaccharide units were proposed by analogy with the co-occurring glycoside **1**. Hence, the structure of pacificusoside F (**3**) was established as 3β-*O*-{3-*O*-methyl-β-d-xylopyranosyl-(1→3)-6-sulfoxy-β-d-glucopyranosyl-(1→4)-β-d-quinovopyranosyl-(1→2)-β-d-xylopyranosyl}-16β-acetoxyholosta-7,22*E*,24-triene. To the best of our knowledge, compound **3** contains a new type of oligosaccharide chain never found before in the sea cucumber triterpene glycosides.

The molecular formula of **5** was determined to be C_54_H_82_O_22_ from the [M+Na]^+^ sodium adduct ion peak at *m/z* 1105.5195 (calculated for [C_54_H_82_O_22_Na]^+^, 1105.5190) in the (+)HRESIMS (Figure S32). The IR spectrum of compound **5** showed the presence of hydroxy (3419 cm^–1^), γ-lactone (1740 cm^–1^), and olefinic (1618 cm^–1^) groups (Figure S33). Compounds **5** and **2** have the same molecular weight. The thorough comparison of the ^1^H and ^13^C NMR data of **5** and **2** showed that they differed from each other only in signals of their side chains (Table 1, Figures S34–S41). The NMR chemical shifts of the aglycon side chain of **5** demonstrated the presence of three methyl groups (*δ*_H_ 1.71 s, 1.74 s, 1.70 s; *δ*_C_ 29.0, 17.5, 26.0) and a conjugated 22,24-diene system (*δ*_H_ 5.79 d (*J* = 12.2), 6.13 t (*J* = 12.2), 6.49 d (12.2); *δ*_C_ 132.1, 120.3, 121.1, 136.8). The existence of the conjugated diene at C-22 was confirmed by the UV spectrum (λ_max_ = 241 nm in MeOH) (Figure S42). The protons sequence from H-22 to H-27, correlated with the corresponding carbon atoms, in the side chain of **5** was assigned using the COSY and HSQC experiments (Table 1, Figures S43 and S44). The key HMBC correlations H_3_-21/C-17, C-20, C-22; H-22/C-17, C-20, and H-23/C-20, C-21, and key ROESY correlations H_3_-21/H-17, H-22, H-23; H-24/H_3_-26, H_3_-27 supported the total structure of the Δ^22,24^-lanostane side chain (Figure 2A,B, Figure S45 and S46). The *cis* configuration of the 22(23)-double bond followed from *J*_22,23_ = 12.2 Hz. Thus, the aglycon of glycoside **5** was an identical aglycon of the known cucumarioside D (**4**) [29]. Therefore, the structure of pacificusoside G (**5**) was established as 3β-*O*-{β-d-glucopyranosyl-(1→4)-[β-d-xylopyranosyl-(1→2)]-β-d-quinovopyranosyl-(1→2)-β-d-xylopyranosyl}-16β-acetoxyholosta-7,22*Z*,24-triene.

The molecular formula of **6** was determined to be C_55_H_83_O_25_SNa from the [M–Na]^–^ ion peak at *m/z* 1175.4937 (calculated for [C_55_H_83_O_25_S]^–^, 1175.4950) in the (–)HRESIMS spectrum (Figure S47). The IR spectrum of compound **6** showed the presence of hydroxy (3421 cm^–1^), γ-lactone (1744 cm^−1^), olefinic (1635 cm^−1^), and sulfate (1245, 819 cm^−1^) groups (Figure S48). Compounds **6** and **3** have the same molecular weight. On the basis of an extensive 2D NMR analysis of glycosides **3** and **6,** we suggested that the oligosaccharide moiety of **3** is identical to that of glycoside **6**. Moreover, a detailed NMR analysis of the proton and carbon signals of the triterpene aglycon of **6** clearly indicated that glycosides **6** and **5** have the same aglycon. All proton and carbon resonances of **6** were received from the ^1^H-^1^H COSY, HSQC, HMBC, and ROESY experiments and confirmed the structure of both the aglycon and carbohydrate moieties (Table 1 and Table 2, Figures S49–S54). Accordantly, the structure of pacificusoside H (**6**) was established to be 3β-*O*-{3-*O*-methyl-β-d-xylopyranosyl-(1→3)-6-*O*-6-sulfoxy-β-d-glucopyranosyl-(1→4)-β-d-quinovopyranosyl-(1→2)-β-d-xylopyranosyl}-16β-acetoxyholosta-7,22*Z*,24-triene.

The molecular formula of **7** was determined to be C_54_H_84_O_25_ from the [M – H]^–^ ion peak at *m/z* 1131.5221 (calculated for [C_54_H_83_O_25_]^–^, 1131.5229) in the (–)HRESIMS (Figure S55). The IR spectrum of compound **7** showed the presence of hydroxy (3413 cm^−1^), γ-lactone (1770 cm^−1^), and olefinic (1633 cm^−1^) groups (Figure S56). The ^1^H and ^13^C NMR spectroscopic data belonging to the pentacyclic moiety of the aglycon of **7** showed the resonances of protons and carbons of four methyl groups (*δ*_H_ 1.04 s, 1.09 s, 1.31 s, 1.36 s; *δ*_C_ 23.8, 17.0, 28.5, 33.9), the 7(8)-double bond (*δ*_H_ 5.65 brd (*J* = 7.1); *δ*_C_ 122.6, 147.4), and lactone fragment (*δ*_C_ 180.6) (Table 1, Figures S57 and S58). The signals of the OAc group in the ^1^H and ^13^C NMR spectra of compound **7** were absent. The ^1^H-^1^H COSY and HSQC correlations attributable to the triterpene nucleus revealed the corresponding sequences of protons from C-1 to C-3, C-5 to C-7, C-9 to C-12 through C-11, and C-15 to C-17 (Figure 2A, Figures S59 and S60). Key HMBC cross-peaks, such as H-2/C-10; H-3/C-4, C-30, C-31; H-5/C-4, C-19; H-7/C-9; H-12/C-13, C-14, C-18; H-15/C-8, C-32; H-16/C-18; H-17/C-13, C-14, C-20, C-21, C-22; H_3_-19/C-1, C-5, C-9, C-10; H_3_-30/C-3, C-4, C-31; H_3_-31/C-4, C-5, C-30; H_3_-32/C-8, C-13, C-14, C-15, confirmed the overall structure of the pentacyclic terpene moiety of **7** (Figure 2A and Figure S61). The key ROESY cross-peaks showed the common 5α/9β/10β/13β,14α stereochemistry of the triterpene nucleus and 3β-configuration of the oxygenated substituent in **7** (Figure 2B and Figure S62). The proton and carbon signals of the aglycon side chain of **7** demonstrated the presence of only one methyl group (*δ*_H_ 1.76 s; *δ*_C_ 23.0) and one 20,22-double bond (*δ*_H_ 5.07 s, 4.98 s; *δ*_C_ 139.9, 113.9) (Table 1, Figures S57 and S58). The key HMBC correlations H_3_-21/C-17, C-20, C-22 and H-22/C-17, C-21 and ROESY correlations H_3_-21/H-16, H-17, H-22, and H_2_-22/H-16 supported the total structure of the side chain (Figure 1, Figures S61 and S62). The NMR spectroscopic data of the aglycon part of **7** were coincident with those of the known cucumarioside A_10_ from *E. fraudatrix* and pacificusoside B from *S. pacificus* with 23,24,25,26,27-pentanor-lanosta-7,20(22)-diene-18(16)-lactone-3β-ol aglycon [28,33].

On the basis of extensive 2D NMR and MS analysis of glycosides **1**, **4**, and **7**, we suggested that the oligosaccharide moiety of **7** is identical to those of glycosides **1** and **4**. Thus, the structure of pacificusoside I (**7**) was elucidated to be 3β-*O*-{3-*O*-methyl-β-d-glucopyranosyl-(1→3)-β-d-glucopyranosyl-(1→4)-[β-d-xylopyranosyl-(1→2)]-β-d-quinovopyranosyl-(1→2)-β-d-xylopyranosyl}-23,24,25,26,27-pentanor-5α-lanosta-7,20(22)-diene-18(16)-lactone.

The comparison of ^1^H, ^13^C NMR and MS spectra and the application of extensive 2D NMR analysis for compounds **8**, **9**, and **7** exhibited that they have the identical triterpene aglycon and differ from each other only in the oligosaccharide moieties (Table 1 and Table 2).

The molecular formula of **8** was determined to be C_47_H_72_O_20_ from the [M + Na]^+^ sodium adduct ion peak at *m/z* 979.4512 (calculated for [C_47_H_72_O_20_Na]^+^, 979.4509) in the (+)HRESIMS spectrum (Figure S63).The IR spectrum of compound **8** showed the presence of hydroxy (3415 cm^−1^), γ-lactone (1766 cm^−1^), and olefinic (1638 cm^−1^) groups (Figure S64). The examination of the ^1^H, ^13^C NMR and MS spectra of compounds **8**, **2**, and **5** clearly revealed that glycoside **8** has the same oligosaccharide chain (Table 2, Figures S65–S70). Therefore, the structure of pacificusoside J (**8**) was established as 3β-*O*-{β-d-glucopyranosyl-(1→4)-[β-d-xylopyranosyl-(1→2)]-β-d-quinovopyranosyl-(1→2)-β-d-xylopyranosyl}-23,24,25,26,27-pentanor-5α-lanosta-7,20(22)-diene-18(16)-lactone.

The molecular formula of **9** was determined to be C_48_H_73_O_23_SNa from the [M – Na]^–^ ion peak at *m/z* 1049.4262 (calculated for [C_48_H_73_O_23_S]^–^, 1049.4269) in the (–)HRESIMS spectrum (Figure S71). The IR spectrum of compound **9** showed the presence of hydroxy (3415 cm^−1^), γ-lactone (1744 cm^−1^), olefinic (1638 cm^−1^), and sulfate (1241, 815 cm^–1^) groups (Figure S72). The detailed comparison of the ^1^H, ^13^C and 2D NMR and MS spectra of glycosides **9**, **3**, and **6** exhibited that the oligosaccharide moiety of **9** is identical to that of **3** and **6** (Table 2, Figures S73–S84). Accordantly, the structure of pacificusoside K (**9**) was established to be 3β-*O*-{3-*O*-methyl-β-d-xylopyranosyl-(1→3)-6-*O*-6-sulfoxy-β-d-glucopyranosyl-(1→4)-β-d-quinovopyranosyl-(1→2)-β-d-xylopyranosyl}-23,24,25,26,27-pentanor-5α-lanosta-7,20(22)-diene-18(16)-lactone.

Previously, only three triterpene glycosides were isolated from two species of starfish, *A. rollestoni* and *C. granulatus* [26,27], and we also reported about six triterpene glycosides from *S. pacificus* [28]. Now, we have isolated nine additional compounds of this type. Pacificusosides D–K (**1**–**3, 5**–**9**) from *S. pacificus* have a structural similarity to the triterpene glycosides obtained from sea cucumber *E. fraudatrix* earlier. In particular, these glycosides contain a holostane type of aglycons, having a 16β-OAc and 7(8)-double bond in the nucleus and *trans-* or *cis-*Δ^22,24^ side chains or a rare non-holostane type of aglycons, namely 23,24,25,26,27-pentanor-lanosta-7,20(22)-diene-18(16)-lactone-3β-ol. Their oligoglycoside chains contain 3-*O*-methyl-d-xylose or 3-*O*-methyl-d-glucose as a terminal monosaccharide unit. For instance, the tetrasaccharide carbohydrate chain without 3-*O*-methyl-d-glucose or 3-*O*-methyl-d-xylose as a terminal monosaccharide unit, in glycosides **2**, **5**, and **8**, was found. To the best of our knowledge, triterpene glycosides with this type of carbohydrate chain are very rare triterpene glycosides. In addition, a tertrasaccharide carbohydrate chain containing a 6-*O*-SO_3_-glucopyranose residue, in glycosides **3**, **6**, and **9**, was not earlier found in echinoderms.

Previously, we investigated the metabolite profiling of triterpene glycosides of the Far Eastern sea cucumber *E. fraudatrix* using LC-ESI QTOF-MS [34]. Totally, 54 triterpene glycosides were detected by this method, including compounds **1**, **2**, and **5.** However, even such a sensitive method as LC-ESI QTOF-MS did not allow detecting and predicting the structures of compounds **3** and **6**–**9.** This fact may also indicate that the starfish *S. pacificus* can partially metabolize the triterpene glycosides of sea cucumbers using their own enzyme systems. It is worth noting that at the present time, in the starfish *S. pacificus*, we did not find polar steroid compounds—typical secondary metabolites of sea stars. At the same time, triterpene glycosides from starfish *A. rollestoni* and *C. granulatus* were isolated together with steroid glycosides that make the detection of triterpene glycosides in the starfish *S. pacificus* unique [26,27]. Probably, dietary glycosides play the same protective biological role against predators and pathogens in this starfish as they play in holothurians.

### 2.2. The Biological Activity Investigation

#### 2.2.1. The Effect of the Triterpene Glycosides on Cell Viability

To determine the cytotoxic activities of triterpene glycosides **1**–**9** from the starfish *S. pacificus*, non-cancerous mouse epidermal cells JB6 Cl41 and the panel of human melanoma cell lines SK-MEL-2, SK-MEL-28, and RPMI-7951 were treated by the investigated compounds at a concentrations range of 0.1–62.5 µM for 24 h, and cell viability was estimated by MTS assay. As shown in Table 3, compounds **1**, **3**, **4**, **6**, and **9** possessed the highest cytotoxic effects against SK-MEL-2 cell lines with IC_50_ of 0.7, 0.68, 0.67, 0.69, and 0.75 µM, respectively. As for SK-MEL-28 cells, compounds **1**, **4**, and **6** inhibited their cell viability with IC_50_ of 8.4, 8.3, and 8.2 µM, respectively (Table 3). The triterpene glycosides **2**, **5**, **7**, and **8** were found to be less effective against the tested cells (Table 3). The RPMI-7951 cell line seemed to be resistant to the action of the investigated compounds (Table 3). In this work, mouse non-cancerous epidermal cells JB6 Cl41 were used as a reference to check compounds **1**–**9** for cancer selectivity. The selectivity indexes of triterpene glycosides **1**–**9** were calculated as the ratio of respective IC_50_ values against cancer cell lines SK-MEL-2, SK-MEL-28, and RPMI-7951 and that against JB6 Cl41 normal cells (Table 3). Ideally, the potential drug should kill the cancer cells, but it should not affect the normal cells, so the higher the magnitude of the selectivity index, the greater is its cancer selectivity [35]. Thus, compounds **1**, **3**, **4**, **6**, and **9** showed a high selectivity (more than nine) only against the SK-MEL-2 cell line.

Since the most known triterpene glycosides belong to a class of saponins and possess membranolytic and hemolytic activities, the hemolytic activity of investigated compounds **1**–**9** was studied. It was found that compounds **1**, **3**, **4**, and **6** have high cytotoxic activity and were able to lyse the erythrocytes with ED_50_ of 2.03, 0.72, 2.48, and 1.68 µM, respectively (Table 4). Based on obtained results, the triterpene glycosides **1**–**9** from *S. pacificus* were used at low non-toxic concentrations of 0.1 μM for further study of their biological activity.

#### 2.2.2. The Effect of the Triterpene Glycosides on Neoplastic Cell Transformation Induced by Carcinogenic Factors

**Figure 4:**
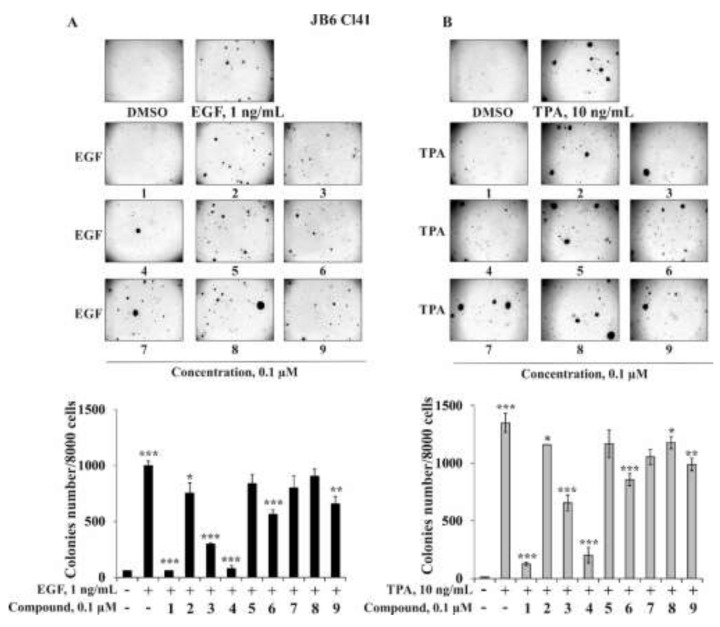
The effect of the triterpene glycosides **1**–**9** from *S. pacificus* on neoplastic cells transformation of JB6 Cl41 induced by chemical carcinogens. JB6 Cl41 cells (2.4 × 10^4^/mL) treated with/without (**A**) EGF (1 ng/mL) and investigated compound (0.1 µM) or (**B**) TPA (10 ng/mL) or investigated compound (0.1 µM) in agar mix. The culture was maintained at 37 °C in a 5% CO_2_ atmosphere for 2 weeks. The colonies were counted under a microscope with the aid of the ImageJ software program (*n* = 9 for control and each compound; *n*—quantity of photos). The magnification of representative photos of colonies is ×10. The asterisks (* *p* < 0.05, ** *p* < 0.01, *** *p* < 0.001) indicate a significant decrease of colony formation in cells treated with compound compared with the EGF- or TPA-treated cells.

The initial stage of cancer development is the neoplastic transformation of non-cancerous cells into cancer ones induced by chemical (growth factors: epidermal growth factor (EGF), vascular endothelial growth factor (VEGF), transforming growth factor β (TGF-β), formaldehydes, nitrites, peroxides, etc.), physical (ionizing radiation: UV and X-ray), and biological (oncogenic viruses, some bacteria) carcinogens [36,37]. In light of this, the investigation of effective and non-toxic cancer-preventive compounds could be a winning weapon to manage cancer initiation. The promotion-sensitive mouse epidermal cells JB6 Cl41 are known to respond irreversibly to carcinogens such as EGF, 12-*O*-tetradecanoilphorbol 13-acetate (TPA), or UV irradiation with the induction of anchorage-independent growth in soft agar [38]. That is why this well-established culture system was used to identify the effect of the triterpene glycosides **1**–**9** from starfish *S. pacificus* on EGF-, TPA-, X-ray-, or UVB-induced neoplastic cell transformation. JB6 Cl41 cells without EGF or TPA treatment did not form colonies in soft agar (negative control), while under the action of EGF (1 ng/mL) or TPA (10 ng/mL), they were transformed and formed colonies (positive control) (Figure 4A,B). Compounds **1**, **3**, **4**, **6**, and **9** at a non-toxic concentration of 0.1 μM significantly inhibited EGF-induced neoplastic transformation of JB6 Cl41 cells by 94%, 70%, 92%, 44%, and 34%, respectively, (Figure 4A) or TPA-induced neoplastic transformation by 98%, 36%, 99%, 61%, and 42%, respectively, compared with the positive control (Figure 4B).

The percentage of inhibition of neoplastic cell transformation of JB6 Cl41 cells of compounds **2**, **5**, **7**, and **8** was 25%, 16%, 20%, and 10% for EGF-induced cell transformation and 18%, 17%, 33%, and 20% for TPA-induced cell transformation compared to the positive control (Figure 4A,B).

People are exposed to both natural sources of ionizing radiation (UV, soil, water, plants) and artificial sources (X-rays and medical devices). As the use of ionizing radiation increases, the potential for health hazards also increases. It was shown that low doses of ionizing radiation (X-rays and UV) can increase the risk of longer-term effects such as cancer, especially skin cancer.

**Figure 5:**
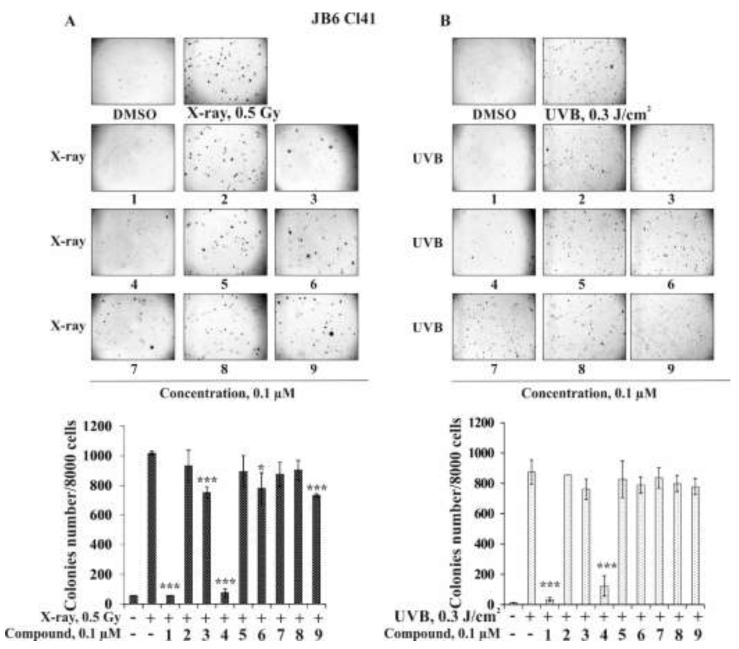
The effect of the triterpene glycosides **1**–**9** from *S. pacificus* on neoplastic cells transformation of JB6 Cl41 induced by ionizing radiation. JB6 Cl41 cells (2.4 × 10^4^/mL) treated with/without (**A**) X-rays (0.5 Gy/9 times) and investigated compound (0.1 µM) or (**B**) UVB (0.3 mJ/cm^2^) or investigated compound (0.1 µM) in 1 mL of 0.3% basal medium Eagle (BME’s) agar containing 10% FBS and overlaid with 3.5 mL of 0.5% BME’s agar containing 10% FBS. The culture was maintained at 37 °C in a 5% CO_2_ atmosphere for 2 weeks. The colonies were counted under a microscope with the aid of the ImageJ software program (*n* = 9 for control and each compound; *n*—quantity of photos). The magnification of representative photos of colonies is ×10. The asterisks (* *p* < 0.05, *** *p* < 0.001) indicate a significant decrease of colony formation in cells treated with compound compared with the X-ray- or UVB-treated cells.

In this work, we hypothesized that the triterpene glycosides from starfish *S. pacificus* were able to prevent the neoplastic cell transformation induced by low doses of X-ray and UVB radiation. The optimal doses and time of cells treatment were experimentally chosen, so in a soft agar the formation and growth of colonies of JB6 Cl41 cells were observed under the irradiation of cells by 0.5 Gy of X-rays or by 0.3 J/cm^2^ of UVB (wavelength 312 nm) three times in week for three weeks (Figure 5A,B). The triterpene glycosides **1** and **4** at 0.1 µM were found to almost completely suppress X-ray-induced cell transformation by 94% and 97%, respectively. Compounds **3**, **6**, and **9** at the same concentration inhibited JB6 Cl41 colonies formation by 26%, 23%, and 28%, respectively (Figure 5A). Other investigated compounds, **2**, **5**, **7**, and **8,** possessed slight cancer-preventive activity; the percentage of inhibition was less than 15% (Figure 5A). It was shown that the inhibiting effect of compounds **1**–**9** on UVB-induced neoplastic cell transformation was weaker than on X-ray-induced cell transformation. The most effective compounds, **1** and **4** (0.1 µM), decreased the colonies number by 96% and 86%, respectively, while compounds **3**, **6**, and **9** inhibited JB6 Cl41 cell transformation induced by UVB by 13%, 10%, and 11%, respectively. Triterpene glycosides **2**, **5**, **7**, and **8** at the same concentration were not effective in the prevention of UVB-induced neoplastic cell transformation.

To the best of our knowledge, an investigation of the inhibition of the EGF-, TPA-, X-ray-, and UVB-induced neoplastic cell transformation of JB6 Cl41 cells by triterpene glycosides has been described here for the first time.

**Figure 6:**
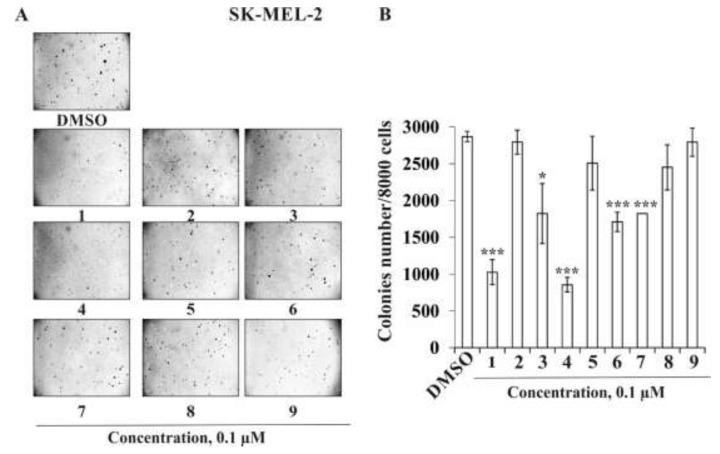
The effect of the triterpene glycosides **1**–**9** from *S. pacificus* on colony formation of melanoma SK-MEL-2 cells. (**A**,**B**) SK-MEL-2 cells (2.4 × 10^4^/mL) treated with/without and investigated compound (0.1 µM) in agar mix. The culture was maintained at 37 °C in a 5% CO_2_ atmosphere for 2 weeks. The colonies were counted under a microscope with the aid of the ImageJ software program (*n* = 9 for control and each compound; *n*—quantity of photos). The magnification of representative photos of colonies is ×10. The asterisks (* *p* < 0.05, *** *p* < 0.001) indicate a significant decrease of colony formation in cells treated with compound compared with the X-ray- or UVB-treated cells.

Next, we determined the influence of the investigated triterpene glycosides on the colony formation of human melanoma cells SK-MEL-2. As in the experiments described above, compounds **1** and **4** at a non-toxic concentration of 0.1 µM possessed the highest activity among the investigated compounds and decreased the colonies number of SK-MEL-2 cells by 64% and 70%, respectively; compounds **3** and **6** decreased the number at the same concentration by 34% and 40%, respectively, while compounds **2**, **5**, **7**, and **8** inhibited colony formation by 3%, 13%, 15%, and 3%, respectively (Figure 6A,B).

We were able to isolate nine compounds (**1**–**9**), which consist of three types of triterpene aglycons and three types of oligosaccharide chains. It was found that compounds **1** and **4** were the most active compounds in all test systems, compounds **3, 6**, and **9** possessed moderate activities, and compounds **2**, **5**, **7**, and **8** are not active. This indicates that the biological activity of triterpene glycosides depends both on the structure of the triterpene aglycon (in particular, the side chain) and on the structure of the carbohydrate chain. Indeed, the most active compounds, **1** and **4,** have *trans-* and *cis-*Δ^22,24^-lanostane side chains in triterpene aglycon and a pentasaccharide chain with terminal 3-*O*-methyl-d-glucose. The least active compounds, **2**, **5**, **7**, and **8,** have a tetrasaccharide chain without a terminal 3-*O*-methyl-monosaccharide (**2** and **5**) or non-holostane type of aglycon (23,24,25,26,27-pentanorlanosta-7,20(22)-diene-18(16)-lactone-3β-ol (**7** and **8**)), respectively. It is obvious that such structural changes lead to an almost complete loss of biological activity. While compounds **3**, **6**, and **9** consisted of a tetrasaccharide chain with terminal 3-*O*-methyl-D-xylose, they demonstrated moderate activity regardless of the structure of the triterpene aglycon.

## 3. Materials and Methods

### 3.1. General Methods

Optical rotations were determined on a PerkinElmer 343 polarimeter (Waltham, MA, USA). UV spectra were recorded on a Shimadzu UV-1601 PC spectrophotometer. IR spectra were recorded using an Equinox 55 spectrophotometer (Bruker, Bremen, Germany). The ^1^H and ^13^C NMR spectra were recorded on Bruker Avance III 700 spectrometer (Bruker, Bremen, Germany) at 700.13 and 176.04 MHz, respectively, and chemical shifts were referenced to the corresponding residual solvent signal (*δ*_H_ 3.30/*δ*_C_ 49.0 for CD_3_OD). The HRESIMS spectra were recorded on a Bruker Impact II Q-TOF mass spectrometer (Bruker, Bremen, Germany); the samples were dissolved in MeOH (c 0.001 mg/mL). HPLC separations were carried out on an Agilent 1100 Series chromatograph (Agilent Technologies, Santa Clara, CA, USA) equipped with a differential refractometer; Diasorb-130-C16T (11 µm, 250 × 16 mm, Biochemmack, Moscow, Russia), Diasfer-110-C18 (10 µm, 250 × 15 mm, Biochemmack, Moscow, Russia), and Discovery C_18_ (5 µm, 250 × 4 mm, Supelco, North Harrison, PA, USA) columns were used. Low-pressure liquid column chromatography was carried out with Polychrome 1 (powdered Teflon, 0.25−0.50 mm; Biolar, Olaine, Latvia) and Si gel KSK (50–160 µm, Sorbpolimer, Krasnodar, Russia). Sorbfil Si gel plates (4.5 × 6.0 cm, 5–17 µm, Sorbpolimer, Krasnodar, Russia) were used for thin-layer chromatography.

### 3.2. Animal Material

Specimens of *S. pacificus* Djakonov, 1938 (order Valvatida, family Solasteridae) were collected at a depth of 10–20 m in the Sea of Okhotsk near Iturup Island during the research vessel *Akademik Oparin’s* 42th scientific cruise in August 2012. Species identification was carried out by Mr. B.B. Grebnev (G.B. Elyakov Pacific Institute of Bioorganic Chemistry of the FEB RAS, Vladivostok, Russia). A voucher specimen (no. 042-112) is on deposit at the marine specimen collection of the G.B. Elyakov Pacific Institute of Bioorganic Chemistry of the FEB RAS, Vladivostok, Russia.

### 3.3. Extraction and Isolation

The fresh animals (1.7 kg) were chopped and extracted twice with EtOH at 20 °C. The H_2_O/EtOH layer was evaporated, and the residue was dissolved in H_2_O (0.5 L). The H_2_O-soluble materials were passed through a Polychrome 1 column (6.5 cm × 21 cm), eluted with distilled H_2_O until a negative chloride ion reaction was obtained and eluted with EtOH. The combined EtOH eluate was evaporated to give a brownish residue (48 g). This material was chromatographed over a Si gel column (6 cm × 22 cm) using CHCl_3_/EtOH (stepwise gradient, 8:1 to 1:1), EtOH, and EtOH/H_2_O (4:1, 2:1, and 1:2) to yield fifteen fractions, 1–15, that were then analyzed by TLC on Si gel plates in the eluent system BuOH/EtOH/H_2_O (4:1:2). HPLC separation of fractions 7 (715 mg) and 8 (178mg) on a Diasorb-130-C16T column (11 µm, 250 × 16 mm, 2.5 mL/min) with EtOH/H_2_O (65:35) as an eluent system followed by the further separation on a Diasfer-110-C18 column (10 μm, 250 mm × 15 mm, 2.5 mL/min) with EtOH/H_2_O (55:45) as an eluent system yielded pure **1** (8.5 mg, *t*_R_ 35.4 min), **2** (4.0 mg, *t*_R_ 38.2 min), **4** (8.0 mg, *t*_R_ 29.7 min), **5** (5.7 mg, *t*_R_ 31.7 min), **6** (3.8 mg, *t*_R_ 16.5 min), **8** (4.5 mg, *t*_R_ 17.6 min), and subfractions 71-6 (36 mg), 71-7 (8.5 mg), and 81-2 (11.5 mg). HPLC separation of fractions 71-6 and 71-7 on a Discovery C18 column (5 μm, 250 mm × 10 mm, 2.5 mL/min) with EtOH/H_2_O (50:50) as an eluent system yielded pure **3** (9.5 mg, *t*_R_ 16.6 min), **7** (3.0 mg, *t*_R_ 15.8 min), and fraction 81-2. HPLC separation of fraction 81-2 on a Discovery C18 column (5 μm, 250 mm × 10 mm, 2.5 mL/min) with EtOH/H_2_O (45:55) as an eluent system yielded pure **9** (5.2 mg, *t*_R_ 7.5 min).

### 3.4. Spectral Data of New Compounds

*Pacificusoside D (**1**),* C_61_H_94_O_27_, amorphous powder; ${\left[\alpha \right]}_{D}^{25}$ –38.6° (c 0.35, MeOH); UV (MeOH) *λ*_max_ 196.5, 240 nm; IR (KBr): *ν*_max_ = 3418, 2874, 1735, 1622, 1542, 1435, 1339, 1214, 1028 cm^−1^; ^1^H and ^13^C NMR data are listed in Table 1 and Table 2; (+)ESIMS/MS of the ion [M + Na]^+^ at *m/z* 1281: 1221 [(M + Na) – C_2_H_4_O_2_]^+^; 1149 [(M + Na)–C_5_H_8_O_4_]^+^; 1105 [(M + Na) – C_7_H_12_O_5_]^+^; 973 [(M + Na) – C_7_H_12_O_5_ – C_5_H_8_O_4_]^+^; 811 [(M + Na) – C_7_H_12_O_5_ – C_5_H_8_O_4_ – C_6_H_10_O_5_]^+^; 789 [carbohydrate chain + Na]^+^; 639 [(carbohydrate chain + Na) – C_5_H_8_O_4_ – H_2_O]^+^; 507 [(carbohydrate chain + Na)–2 × C_5_H_8_O_4_ – H_2_O]^+^; 361 [(carbohydrate chain+Na)–2 × C_5_H_8_O_4_ – H_2_O – C_6_H_10_O_4_]^+^; 217 [C_7_H_12_O_5_ + Na + H_2_O]^+^; 185 [C_6_H_10_O_5_ + Na]^+^; (+)HRESIMS *m/z* 1281.5879 [M + Na]^+^ (calcd for [C_61_H_94_O_27_Na]^+^, 1281.5875); (–)ESIMS/MS of the ion [M – H]^–^ at *m/z* 1257: 1125 [(M – H) – C_5_H_8_O_4_]^–^; 1081 [(M – H) – C_7_H_12_O_5_]^–^; 949 [(M – H) – C_7_H_12_O_5_ – C_5_H_8_O_4_]^–^; 919 [(M – H) – C_7_H_12_O_5_ – C_6_H_10_O_5_]^–^; 787 [(M – H) – C_7_H_12_O_5_ – C_5_H_8_O_4_ – C_6_H_10_O_5_]^–^; 641 [(M – H) – C_7_H_12_O_5_ – C_5_H_8_O_4_ – C_6_H_10_O_5_ – C_6_H_10_O_4_]^–^.

*Pacificusoside E (**2**),* C_54_H_82_O_22_, amorphous powder; ${\left[\alpha \right]}_{D}^{25}$ –71.7° (c 0.1, MeOH); IR (KBr): *ν*_max_ = 3414, 2873, 2830, 1736, 1619, 1598, 1522, 1418, 1340, 1215, 1162, 1028 cm^−1^; ^1^H and ^13^C NMR data are listed in Table 1 and Table 2; (+)ESIMS/MS of the ion [M + Na]^+^ at *m/z* 1105: 1045 [(M + Na) – C_2_H_4_O_2_]^+^; 973 [(M + Na) – C_5_H_8_O_4_]^+^; 943 [(M + Na) – C_6_H_10_O_5_]^+^; 811 [(M + Na) – C_6_H_10_O_5_ – C_5_H_8_O_4_]^+^; 613 [carbohydrate chain + Na]^+^; 463 [(carbohydrate chain + Na) – C_5_H_8_O_4_ – H_2_O]^+^; 331 [(carbohydrate chain + Na) – 2 × C_5_H_8_O_4_ – H_2_O]^+^; 185 [(carbohydrate chain + Na)–2 × C_5_H_8_O_4_ – H2O – C_6_H_10_O_4_]^+^; (+)HRESIMS *m/z* 1105.5183 [M + Na]^+^ (calcd for [C_54_H_82_O_22_Na]^+^, 1105.5190); (–)ESIMS/MS of the ion [M – H]^–^ at *m/z* 1081: 949 [(M – H) – C_5_H_8_O_4_]^–^; 919 [(M – H) – C_6_H_10_O_5_]^–^; 787 [(M – H) – C_6_H_10_O_5_ – C_5_H_8_O_4_]^–^; 641 [(M – H) – C_6_H_10_O_5_ – C_5_H_8_O_4_ – C_6_H_10_O_4_]^–^.

*Pacificusoside F (**3**),* C_55_H_83_O_25_SNa, amorphous powder; ${\left[\alpha \right]}_{D}^{25}$ –22.0° (c 0.05, MeOH); IR (KBr): *ν*_max_ = 3415, 2926, 2855, 1763, 1742, 1638, 1619, 1461, 1378, 1243, 11180, 1166, 1115, 1074, 817 cm^−1^; ^1^H and ^13^C NMR data are listed in Table 1 and Table 2; (+)ESIMS/MS of the ion [M + Na]^+^ at *m/z* 1221: 1177 [(M + Na) – CO_2_]^+^; 1161 [(M + Na) – C_2_H_4_O_2_]^+^; 1075 [(M + Na) – C_6_H_10_O_4_]^+^; 1015 [(M + Na) – C_6_H_10_O_4_ – C_2_H_4_O_2_]^+^; 971 [(M + Na) – C_6_H_10_O_4_ – C_2_H_4_O_2_ – CO_2_]^+^; 711 [(carbohydrate chain + Na)]^+^; 579 [(carbohydrate chain + Na) – C_5_H_8_O_4_]^+^; 433 [(carbohydrate chain + Na) – C_5_H_8_O_4_ – C_6_H_10_O_4_]^+^; 287 [(carbohydrate chain + Na) – C_5_H_8_O_4_ – 2 × C_6_H_10_O_4_]^+^; (–)ESIMS/MS of the ion [M – Na]^–^ at *m/z* 1175: 1131 [(M – Na) – CO_2_]^–^; 1115 [(M – Na) – C_2_H_4_O_2_]^–^; 1071 [(M – Na) – C_2_H_4_O_2_ – CO_2_]^–^; 1029 [(M – Na) – C_6_H_10_O_4_]^–^; 665 [(carbohydrate chain – Na)]^–^; 533 [(carbohydrate chain – Na) – C_5_H_8_O_4_]^–^; 387 [(carbohydrate chain – Na) – C_5_H_8_O_4_ – C_6_H_10_O_4_]^–^; 241 [(carbohydrate chain – Na) –C_5_H_8_O_4_–2 × C_6_H_10_O_4_]^–^; (–)HRESIMS *m/z* 1175.4944 [M – Na]^–^ (calcd for [C_55_H_83_O_25_S]^–^, 1175.4950).

*Pacificusoside G (**5**),* C_54_H_82_O_22_, amorphous powder; ${\left[\alpha \right]}_{D}^{25}$ –23.3° (c 0.17, MeOH); UV (MeOH) *λ*_max_ 196.5, 241 nm; IR (KBr): *ν*_max_ = 3419, 2927, 1740, 1618, 1556, 1456, 1384, 1245, 1160, 1071, 1028 cm^−1^; ^1^H and ^13^C NMR data are listed in Table 1 and Table 2; (+)ESIMS/MS of the ion [M + Na]^+^ at *m/z* 1105: 1045 [(M + Na) – C_2_H_4_O_2_]^+^; 973 [(M + Na) – C_5_H_8_O_4_]^+^; 943 [(M + Na) – C_6_H_10_O_5_]^+^; 811 [(M + Na) – C_6_H_10_O_5_ – C_5_H_8_O_4_]^+^; 665 [(M + Na) – C_6_H_10_O_5_ – C_5_H_8_O_4_ – C_6_H_10_O_4_]^+^; 613 [carbohydrate chain + Na]^+^; 533 [(M + Na) – C_6_H_10_O_5_ – 2 × C_5_H_8_O_4_ – C_6_H_10_O_4_]^+^; 463 [(carbohydrate chain + Na) – C_5_H_8_O_4_ – H_2_O]^+^; 331 [(carbohydrate chain + Na) – 2 × C_5_H_8_O_4_ – H_2_O]^+^; 185 [(carbohydrate chain + Na)–2 × C_5_H_8_O_4_ – H2O – C_6_H_10_O_4_]^+^; (+)HRESIMS *m/z* 1105.5195 [M + Na]^+^ (calcd for [C_54_H_82_O_22_Na]^+^, 1105.5190); (–)ESIMS/MS of the ion [M – H]^–^ at *m/z* 1081: 949 [(M – H) – C_5_H_8_O_4_]^–^; 919 [(M – H) – C_6_H_10_O_5_]^–^; 787 [(M – H) – C_6_H_10_O_5_ – C_5_H_8_O_4_]^–^; 641 [(M – H) – C_6_H_10_O_5_ – C_5_H_8_O_4_ – C_6_H_10_O_4_]^–^.

*Pacificusoside H (**6**),* C_55_H_83_O_25_SNa, amorphous powder; ${\left[\alpha \right]}_{D}^{25}$ –5.9° (c 0.35, MeOH); IR (KBr): *ν*_max_ = 3421, 2929, 1744, 1635, 1542, 1457, 1384, 1245, 1073, 1028, 819 cm^−1^; ^1^H and ^13^C NMR data are listed in Table 1 and Table 2; (+)ESIMS/MS of the ion [M + Na]^+^ at *m/z* 1221: 1177 [(M + Na) – CO_2_]^+^; 1161 [(M + Na) – C_2_H_4_O_2_]^+^; 1075 [(M + Na) – C_6_H_10_O_4_]^+^; 971 [(M + Na) – C_6_H_10_O_4_ – C_2_H_4_O_2_ – CO_2_]^+^; 711 [(carbohydrate chain + Na)]^+^; 707 [(M + Na) – C_6_H_10_O_4_ – C_2_H_4_O_2_ – CO_2_ – C_6_H_9_O_8_S]^+^; 579 [(carbohydrate chain + Na) – C_5_H_8_O_4_]^+^; 561 [(M + Na) – C_6_H_10_O_4_ – C_2_H_4_O_2_ – CO_2_ – C_6_H_9_O_8_S – C_6_H_10_O_4_]^+^; 433 [(carbohydrate chain + Na)–C_5_H_8_O_4_ – C_6_H_10_O_4_]^+^; (–)ESIMS/MS of the ion [M – Na]^–^ at *m/z* 1175: 1115 [(M – Na) – C_2_H_4_O_2_]^–^; 1071 [(M – Na) – C_2_H_4_O_2_ – CO_2_]^–^; 1029 [(M – Na) – C_6_H_10_O_4_]^–^; (–)HRESIMS *m/z* 1175.4937 [M – Na]^–^ (calcd for [C_55_H_83_O_25_S]^–^, 1175.4950).

*Pacificusoside I (**7**),* C_54_H_84_O_25_, amorphous powder; ${\left[\alpha \right]}_{D}^{25}$ –36.9° (c 0.12, MeOH); IR (KBr): *ν*_max_ = 3413, 2928, 2853, 1770, 1633, 1457, 1383, 1243, 1161, 1072, 1031 cm^−1^; ^1^H and ^13^C NMR data are listed in Table 1 and Table 2; (+)ESIMS/MS of the ion [M + Na]^+^ at *m/z* 1155: 1023 [(M + Na) – C_5_H_8_O_4_]^+^; 979 [(M + Na) – C_7_H_12_O_5_]^+^; 847 [(M + Na) – C_7_H_12_O_5_ – C_5_H_8_O_4_]^+^; 789 [carbohydrate chain + Na]^+^; 685 [(M + Na) – C_7_H_12_O_5_ – C_5_H_8_O_4_ – C_6_H_10_O_5_]^+^; 539 [(M + Na) – C_7_H_12_O_5_ – C_5_H_8_O_4_ – C_6_H_10_O_5_ – C_6_H_10_O_4_]^+^; (–)ESIMS/MS of the ion [M – H]^–^ at *m/z* 113: 999 [(M – H) – C_5_H_8_O_4_]^–^; 955 [(M – H) – C_7_H_12_O_5_]^–^; 823 [(M – H) – C_7_H_12_O_5_ – C_5_H_8_O_4_]^–^; 661 [(M – H) – C_7_H_12_O_5_ – C_5_H_8_O_4_ – C_6_H_10_O_5_]^–^; 515 [(M – H) – C_7_H_12_O_5_ – C_5_H_8_O_4_ – C_6_H_10_O_5_ – C_6_H_10_O_4_]^–^; 383 [(M – H)–carbohydrate chain]^–^; (–)HRESIMS *m/z* 1131.5221 [M – H]^–^ (calcd for [C_54_H_83_O_25_]^–^, 1131.5229).

*Pacificusoside J (**8**),* C_47_H_72_O_20_, amorphous powder; ${\left[\alpha \right]}_{D}^{25}$ –6.6° (c 0.08, MeOH); IR (KBr): *ν*_max_ = 3415, 2926, 1766, 1638, 1619, 1452, 1383, 1180, 1142, 1130, 1074, 1030 cm^−1^; ^1^H and ^13^C NMR data are listed in Table 1 and Table 2; (+)ESIMS/MS of the ion [M + Na]^+^ at *m/z* 979: 847 [(M + Na) – C_5_H_8_O_4_]^+^; 817 [(M + Na) – C_6_H_10_O_5_]^+^; 685 [(M + Na) – C_6_H_10_O_5_ – C_5_H_8_O_4_]^+^; 613 [carbohydrate chain + Na]^+^; 539 [(M + Na) – C_6_H_10_O_5_ – C_5_H_8_O_4_ – C_6_H_10_O_4_]^+^; 463 [(carbohydrate chain + Na) – C_5_H_8_O_4_ – H_2_O]^+^; 331 [(carbohydrate chain + Na) – 2 × C_5_H_8_O_4_ – H_2_O]^+^; 185 [(carbohydrate chain + Na) – 2 × C_5_H_8_O_4_ – H2O – C_6_H_10_O_4_]^+^; (+)HRESIMS *m/z* 979.4512 [M + Na]^+^ (calcd for [C_47_H_72_O_20_Na]^+^, 979.4509); (–)ESIMS/MS of the ion [M – H]^–^ at *m/z* 955: 823 [(M – H) – C_5_H_8_O_4_]^–^; 661 [(M – H) – C_6_H_10_O_5_ – C_5_H_8_O_4_]^–^; 151 [(M – H) – C_6_H_10_O_5_ – C_5_H_8_O_4_ – C_6_H_10_O_4_]^–^.

*Pacificusoside K (**9**),* C_48_H_73_O_23_SNa, amorphous powder; ${\left[\alpha \right]}_{D}^{25}$ –16.4° (c 0.14, MeOH); IR (KBr): *ν*_max_ = 3415, 2927, 2359, 2341, 1772, 1744, 1638, 1619, 1454, 1382, 1241, 1180, 1165, 1141, 1130, 1074, 815 cm^−1^; ^1^H and ^13^C NMR data are listed in Table 1 and Table 2; (+)ESIMS/MS of the ion [M + Na]^+^ at *m/z* 1095: 1051 [(M + Na) – CO_2_]^+^; 975 [(M + Na) – HNaSO_4_]^+^; 949 [(M + Na) – C_6_H_10_O_4_]^+^; 829 [(M + Na) – C_6_H_10_O_4_ – HNaSO_4_]^+^; 711 [(carbohydrate chain + Na)]^+^; 579 [(carbohydrate chain + Na) – C_5_H_8_O_4_]^+^; 433 [(carbohydrate chain + Na) – C_5_H_8_O_4_ – C_6_H_10_O_4_]^+^; 287 [(carbohydrate chain + Na) – C_5_H_8_O_4_ – 2 × C_6_H_10_O_4_]^+^; (–)ESIMS/MS of the ion [M – Na]^–^ at *m/z* 1049: 1005 [(M – Na) – CO_2_]^–^; 989 [(M – Na) – C_2_H_4_O_2_]^–^; 903 [(M – Na) – C_6_H_10_O_4_]^–^; 665 [(carbohydrate chain – Na)]^–^; 533 [(carbohydrate chain–Na)–C_5_H_8_O_4_]^–^; 387 [(carbohydrate chain–Na)–C_5_H_8_O_4_–C_6_H_10_O_4_]^–^; 241 [(carbohydrate chain–Na)–C_5_H_8_O_4_–2 × C_6_H_10_O_4_]^–^; (–)HRESIMS *m/z* 1049.4262 [M – Na]^–^ (calcd for [C_48_H_73_O_23_S]^–^, 1049.4269).

### 3.5. Acid Hydrolysis and Determination of Absolute Configurations of Monosaccharides

The acid hydrolysis of **1** (3.5 mg) was carried out in a solution of 2 M trifluoroacetic acid (TFA) (1 mL) in a sealed vial on a H_2_O bath at 100 °C for 2 h. The H_2_O layer was washed with CHCl_3_ (3 × 1.0 mL) and concentrated in vacuo. One drop of concentrated TFA and 0.5 mL of L-(–)-2-octanol (Aldrich) were added to the sugar mixture, and the sealed vial was heated on a glycerol bath at 130 °C for 6 h. The solution was evaporated in vacuo and treated with a mixture of pyridine/acetic anhydride (1:1, 0.6 mL) for 24 h at room temperature. The acetylated 2-octylglycosides were analyzed by GC using the corresponding authentic samples prepared by the same procedure. The following peaks were detected in the hydrolysate of **1**: D-quinovose (*t*_R_ 20.59, 20.84, 21.16, and 21.45 min), D-xylose (*t*_R_ 21.00, 21.16, and 21.56 min), D-glucose (*t*_R_ 24.53, 25.13, 25.36, and 25.65 min), and 3-OMe-d-glucose (*t*_R_ 23.30, 24.47, 24.53, and 24.85 min). The retention times of the authentic samples were as follows: D-quinovose (*t*_R_ 20.59, 20.82, 21.18, and 21.42 min), D-xylose (*t*_R_ 21.06, 21.17, and 21.54 min), D-glucose (*t*_R_ 24.51, 25.14, 25.38, and 25.66 min), 3-OMe-d-glucose (*t*_R_ 23.30, 24.48, 24.54, and 24.85 min), l-quinovose (*t*_R_ 20.42, 20.90, and 21.48 min), L-xylose (*t*_R_ 20.78, 21.28, and 21.58 min), l-glucose (*t*_R_ 24.66, 24.93, 25.12, and 25.35 min), and 3-OMe-l-glucose (*t*_R_ 23.75, 24.00, 24.16, and 25.13 min).

### 3.6. Cell Lines and Culture Conditions

American Type Culture Collection (Manassas, VA, USA) provided normal mouse epidermal cells JB6 Cl41 (ATCC^®^ no. CRL-2010™) and human melanoma cells SK-MEL-2 (ATCC^®^ no. HTB-68™), SK-MEL-28 (ATCC^®^ no. HTB-72™), and RPMI-7951 (ATCC^®^ no. HTB-66™). JB6 Cl41 cells were cultured in Minimal Essential Medium (MEM) and SK-MEL-2, SK-MEL-28, and RPMI-7951 cells were maintained in Dulbecco’s Modified Eagle Medium (DMEM), supplemented with 5% and 10% fetal bovine serum (FBS), respectively, and 100 mg/mL streptomycin, 100 U/mL penicillin (complete medium), in a humidified 5% CO_2_ incubator. At 90% confluence, cells were rinsed with PBS, detached from the tissue culture flask by 0.25% trypsin/0.5 mM EDTA, and 10% of the harvested cells were transferred to a new flask containing fresh complete medium. The passage number was carefully controlled, and the mycoplasma contamination was monitored on a regular basis.

### 3.7. Cell Viability Assay

MTS assay was used to measure the cell viability of tested normal and cancer cells. Cells were seeded into 96-well plates (“Jet Biofil”, Guangzhou, China) at density of 1.0 × 10^4^/200 µL for 24 h at 37 °C in a 5% CO_2_ incubator. Then, cell monolayer was treated either with DMSO (control) or various concentrations of the triterpene glycosides **1**–**9** from *S. pacificus* (0.1, 0.5, 2.5, 12.5, 62.5 µM) in fresh appropriate culture medium for 24 h. Subsequently, the cells were incubated with 15 µL of 3-(4,5-dimethylthiazol-2-yl)-5-(3-carboxymethoxyphenyl)-2-(4-sulfophenyl)-2H-tetrazolium (MTS reagent) (“Promega”, Madison, WI, USA) for 3 h, and the absorbance of each well was measured at 490/630 nm using Power Wave XS microplate reader (“BioTek”, Wynusky, VT, USA). The concentration at which the compounds exert half of their maximal inhibitory effect on cell viability (IC_50_) was calculated by the AAT-Bioquest^®^ online calculator [39]. The selectivity index (SI) was calculated as described previously [40] using the following formula SI = IC_50_ of the compounds in normal cell (JB6 Cl41)/IC_50_ of the same compounds in human melanoma cell lines (SK-MEL-2, SK-MEL-28, or RPMI-7951). Both IC_50_ and SI values are indicated in Table 3.

### 3.8. Hemolysis Assay

Human erythrocytes (O(I)+) were obtained from Vladivostok blood transfusion station. Erythrocytes were centrifuged with PBS (pH 7.4) for 5 min at 4 °C by 450× *g* on a LABOFUGE 400R (Heraeus, Hanau, Germany) centrifuge three times. Then, the residue of erythrocytes was resuspended in ice-cold PBS (pH 7.4) to a final optical density of 2.0 at 700 nm and kept on ice. For the hemolytic assay, 180 µL of erythrocyte suspension was mixed with 20 µL of Cucumarioside A_2_-2 (positive control) and investigated compounds at concentrations range of 0.1, 0.5, 1, 5, 10 µM in V-bottom 96-well plates. After 1 h of incubation at 37 °C, plates were centrifuged at 900× *g* for 10 min on an LMC-3000 (Biosan, Riga, Latvia) laboratory centrifuge. Then, we carefully selected 100 µL of supernatant and transferred it to new flat plates, respectively. Lysis of erythrocytes was determined by measuring the concentration of hemoglobin in the supernatant with a microplate photometer Multiskan FC (Thermo Fisher Scientific, Waltham, MA, USA) at 570 nm. The effective dose causing 50% of erythrocytes hemolysis (ED_50_) was calculated using the computer program SigmaPlot 10.0. All experiments were made in triplicate, *p* < 0.01. ED_50_ values are indicated in Table 4.

### 3.9. The Soft Agar Assay

#### 3.9.1. Neoplastic Cell Transformation of Normal Cells Induced by Carcinogenic Factors

JB6 Cl41 cells (2.4 × 10^4^) were exposed to epidermal growth factor (EGF) (1 ng/mL) or phorbol 12-myristate 13-acetate (TPA) (10 ng/mL) and treated either with DMSO (control) or the triterpene glycosides **1**–**9** from *S. pacificus* (0.1, 0.5, and 1 µM) in 1 mL of 0.3% basal medium Eagle (BME) agar containing 10% FBS, 2 mM L-glutamine, and 25 µg/mL gentamicin (agar mix). The cultures were maintained in a humidified atmosphere with 5% CO_2_ at 37 °C for 14 days, and the cell’s colonies were scored using a microscope, Motic AE 20 (XiangAn, Xiamen, China), and the ImageJ software bundled with 64-bit Java 1.8.0_112 (NIH, Bethesda, MD, USA). To determine the effect of investigated compounds on neoplastic cell transformation induced by ionizing radiation (X-ray or UVB), JB6 Cl41 cells (3 × 10^5^/dish) were seeded in 60 mm dishes and cultured for 24 h at 37 °C in 5% CO_2_ incubator. Then, the cells were exposed to X-rays (X-ray system XPERT 80 (KUB Technologies, Inc, Milford, CT, USA)) at dose of 0.5 Gy or UVB (wavelengths below 312 nm) (Bio-Link UV irradiation system, Vilber, France) at dose of 0.3 J/cm^2^ three times a week for 3 weeks. Irradiated JB6 Cl41 cells (2.4 × 10^4^) were treated either with DMSO (control) or the triterpene glycosides **1**–**9** from *S. pacificus* (0.1, 0.5, and 1 µM) in 1 mL of agar mix. The cultures were maintained in a humidified atmosphere with 5% CO_2_ at 37 °C for 14 days, and the cell’s colonies were scored using a microscope, Motic AE 20 (XiangAn, Xiamen 361101, China), and the ImageJ software bundled with 64-bit Java 1.8.0_112 (NIH, Bethesda, MD, USA).

#### 3.9.2. Colony Formation of Cancer Cells

SK-MEL-2 cells (2.4 × 10^4^) were treated either with DMSO (control) or the triterpene glycosides **1**–**9** from *S. pacificus* (0.1 µM) in 1 mL of agar mix. After 14 days, colonies of cancer cells were scored as described above.

### 3.10. Statistical Analysis

All of the assays were performed in at least three independent experiments. Results are expressed as the mean ± standard deviation (SD). The Student’s t-test was used to evaluate the data with the following significance levels: * *p* < 0.05, ** *p* < 0.01, *** *p* < 0.001.

## 4. Conclusions

Thus, nine triterpene glycosides, including eight new pacificusosides D–K, were isolated from the Far Eastern starfish *S. pacificus*, and their chemical structures were established. The glycosides with a tetrasaccharide carbohydrate chain containing a 6-*O*-SO_3_-glucopyranose residue, as in pacificusosides F, H, and K, were not earlier found in echinoderms. Pacificusosides E, G, and J have a very rare tetrasaccharide carbohydrate chain without 3-*O*-methyl-d-glucose or 3-*O*-methyl-d-xylose as a terminal monosaccharide unit.

The isolation of a series of new triterpene glycosides from starfish is a rare case. Pacificusosides D–K have a structural similarity to the triterpene glycosides obtained from the sea cucumber *E. fraudatrix* earlier. Most of these glycosides contain 3-*O*-methyl-d-xylose or 3-*O*-methyl-d-glucose as a terminal monosaccharide unit. These monosaccharide residues are characteristic for the triterpene glycosides from sea cucumber *E. fraudatrix* [34]. Previously, we suggested that the triterpene glycosides from the starfish *S. pacificus* are food markers because they can be obtained by starfish through diet [28]. The discovery of a series of nine triterpene glycosides confirmed our assumptions that the starfish *S. pacificus* feeds mainly on sea cucumbers *E. fraudatrix* or related species. At the same time, these glycosides from *S. pacificus* contain some structural features, differing them from the triterpene glycosides from these sea cucumbers, which suggests the participation of the enzymes of this starfish in the metabolism of dietary compounds.

The anticancer activity against human melanoma cells and the prevention of the EGF-, TPA-, X-ray-, and UVB-induced neoplastic cell transformation of JB6 Cl41 cells of triterpene glycosides were investigated for the first time. It was found that compounds **1**, **3**, **4**, **6**, and **9** possessed the highest cytotoxic effects against SK-MEL-2 cell lines with IC_50_ less than 1 µM with high cancer selectivity (more than nine). Compounds **1**, **3**, **4**, **6**, and **9** at a non-toxic concentration of 0.1 μM significantly inhibited EGF- or TPA-induced neoplastic transformation compared with positive control. Moreover, the triterpene glycosides **1** and **4** at 0.1 µM were shown to almost completely suppress X-ray- or UVB-induced cell transformation. It should be noted that the most active triterpene glycosides, **1** and **4**, possessed cancer preventive and anticancer activities at concentrations much lower than their effective dose of hemolysis.

The investigated compounds (**1**–**9**) consist of three types of triterpene aglycons and three types of oligosaccharide chains, and analysis of their activity allows for revealing the structure–activity relationship. This indicates that the biological activity of triterpene glycosides depends both on the structure of the triterpene aglycon and on the structure of the carbohydrate chain. Indeed, the most active compounds, **1** and **4,** have *trans*- and *cis*-Δ^22,24^-lanostane side chains in triterpene aglycon and a pentasaccharide chain with terminal 3-*O*-methyl-d-glucose. The least active compounds, **2**, **5**, and **7,** in these rows have a tetrasaccharide chain without a terminal 3-*O*-methyl-monosaccharide (**2** and **5**) or non-holostane type of aglycon (**7**), respectively.

As a result, our studies of triterpene glycosides from *S. pacificus* not only revealed the compound leaders (**1** and **4**), which have potent cancer-preventive and anticancer activities, but also brought to light their structure–activity relationships.

## Figures and Tables

**Table 1:** ^1^H (700.13 MHz) and ^13^C (176.04 MHz) NMR data of aglycon parts of compounds **1**–**3** and **5**–**9** (35 °C, C_5_D_5_N, *J* in Hz) ^a^.

Position	1–3		5, 6		7–9	
	*δ*_H_	*δ*_C_	*δ*_H_	*δ*_C_	*δ*_H_	*δ*_C_
1	1.51 m	36.0	1.49 m	36.1	1.49 m	35.9
2	2.22 m 1.97 m	27.1	2.20 m 1.95 m	27.0	2.20 dd (13.4, 4.0) 1.90 m	27.1
3	3.34 dd (12.0, 4.1)	89.2	3.36 dd (11.6, 3.8)	88.9	3.33 dd (11.7, 4.5)	88.8
4		39.5		39.4		39.3
5	1.09 m	48.0	1.07 dd (9.5, 5.7)	47.8	1.01 dd (11.7, 3.5)	47.5
6	2.09 m 2.05 m	23.2	2.07 m	23.2	2.06 m 1.98 m	23.2
7	5.71 m	120.5	5.67 m	120.5	5.65 brd (7.1)	122.6
8		145.5		145.8		147.4
9	3.52 m	47.2	3.49 brd (14.1)	47.3	3.02 brd (14.3)	46.4
10		35.5		35.5		35.5
11	1.85 m 1.59 m	22.4	1.84 m 1.52 m	22.5	2.00 m 1.47 m	21.7
12	2.18 m 2.01 m	30.9	2.17 m 2.03 m	30.7	2.37 ddd (14.2, 10.0, 8.1) 1.88 m	20.0
13		58.9		58.3		56.7
14		47.6		48.0		46.0
15	2.45 dd (12.4, 7.7) 1.84 m	43.0	2.50 dd (12.7, 7.9) 1.78 dd (12.7, 7.9)	43.6	2.17 d (13.7) 1.97 dd (13.7, 2.4)	43.8
16	5.94 q (8.2)	72.9	6.01 q (7.9)	72.6	4.75 brd (2.4)	80.4
17	2.83 d (8.8)	56.1	3.11 d (7.9)	57.4	2.95 s	59.0
18		179.2		179.1		180.6
19	1.24 s	23.9	1.24 s	23.8	1.04 s	23.8
20		83.1		83.8		139.9
21	1.57 s	30.2	1.71 s	29.0	1.76 s	23.0
22	5.92 d (15.7)	134.0	5.79 d (12.2)	132.1	5.07 s 4.98 s	113.9
23	6.56 dd (15.7, 11.0)	122.4	6.13 t (12.2)	120.3		
24	5.86 brd (11.0)	125.2	6.49 d (12.2)	121.1		
25		134.6		136.8		
26	1.61 s	17.9	1.74 s	17.5		
27	1.69 s	25.6	1.70 s	26.0		
30	1.14 s	17.2	1.17 s	17.3	1.09 s	17.0
31	1.34 s	28.6	1.33 s	28.7	1.31 s	28.5
32	1.18 s	32.1	1.12 s	32.3	1.36 s	33.9
CO		170.3		169.6		
CH_3_-CO	1.98 s	21.2	1.98 s	21.2		

^a^ Assignments from 700 MHz ^1^H-^1^H COSY, HSQC, HMBC (8 Hz), and ROESY (270 msec) data.

**Table 2:** ^1^H (700.13 MHz), ^13^C (176.04 MHz), and HMBC NMR data of oligosaccharide chains of **1**–**3** and **5**–**9** (35 °C, C_5_D_5_N, *J* in Hz)^a^.

Position	1, 7			2, 5, 8			3, 6, 9		
	*δ*_H_	*δ*_C_	HMBC	*δ*_H_	*δ*_C_	HMBC	*δ*_H_	*δ*_C_	HMBC
	Xyl-I			Xyl-I			Xyl (= Xyl-I)		
1	4.87 d (7.0)	105.1	C3-agl	4.87 d (7.2)	105.1	C3-agl	4.76 d (7.3)	105.5	C3-agl
2	3.93 dd (8.7, 7.0)	83.2	C1, C3-Xyl-I, C1-Qui	3.94 dd (8.8, 7.2)	83.3	C1, C3-Xyl-I, C1-Qui	4.06 m	83.0	
3	4.20 t (8.7)	77.8	C2-Xyl-I	4.21 t (8.8)	77.8	C2, C4-Xyl-I	4.11 m	77.3	C4-Xyl-I
4	4.13 m	70.4		4.13 m	70.2		4.13 m	70.9	
5	4.34 dd (11.3, 5.2) 3.71 dd (11.3, 9.7)	66.5	C1, C4-Xyl-I	4.33 dd (11.4, 5.3) 3.72 dd (11.4, 9.1)	66.5	C1, C3, C4-Xyl-I C1-Xyl-I	4.28 m 3.61 m	66.5	C4-Xyl-I C1-Xyl-I
	Qui			Qui			Qui		
1	5.20 d (7.8)	103.0	C2-Xyl-I	5.21 d (7.2)	103.0	C2-Xyl-I	5.06 d (7.6)	104.9	C2-Xyl-I
2	4.13 m	82.6	C1, C3-Qui	4.15 m	82.7	C1, C3-Qui	3.96 dd (9.4, 7.6)	76.1	C3-Qui
3	4.10 t (9.0)	75.7	C2-Qui	4.13 m	75.7	C2-Qui	4.01 dd (9.4, 8.6)	74.7	C2-Qui
4	3.60 t (9.0)	86.6	C3, C5, C6-Qui, C1-Glc-I	3.62 m	86.6	C3, C5-Qui	3.54 t (8.6)	88.3	C6-Qui, C1-Glc
5	3.67 m	71.0		3.68 m	71.0		3.76 m	71.5	
6	1.70 d (6.7)	17.9	C4, C5-Qui	1.71 d (6.4)	18.0	C4, C5-Qui	1.69 d (6.1)	17.9	C4, C5-Qui
	Glc (=Glc-I)			Glc			6-OSO_3_-Glc (=Glc)		
1	4.92 d (7.8)	104.7	C4-Qui	4.96 d (8.0)	105.3	C4-Qui	4.85 d (8.1)	104.9	C4-Qui
2	4.00 dd (9.0, 7.8)	73.5	C3-Glc-I	4.01 dd (9.0, 8.0)	74.7	C1, C3-Glc	3.98 t (8.8)	73.7	
3	4.22 t (9.0)	88.0	C2, C4-Glc-I, C1-Glc-II	4.23 t (9.0)	78.1	C2, C4-Glc	4.18 t (8.8)	86.5	C4-Glc, C1-Xyl-II
4	4.05 t (9.0)	69.6	C3, C5, C6-Glc-I	4.18 t (9.0)	71.5	C3, C6-Glc	3.81 t (9.1)	70.1	C3, C5, C6-Glc
5	3.98 m	77.7		4.06 m	78.2		4.29 m	75.2	C4-Glc
6	4.45 dd (11.9, 2.2) 4.18 dd (11.9, 6.5)	61.9		4.55 dd (11.4, 2.1) 4.29 dd (11.4, 6.2)	62.3		5.25 dd (10.4, 2.7) 4.82 dd (10.4, 9.4)	67.6	C5-Glc
	3-OMe-Glc (=Glc-II)			Xyl-II			3-OMe-Xyl (=Xyl-II)		
1	5.27 d (8.0)	105.5	C3-Glc-I	5.39 d (6.9)	105.9	C2-Qui	5.21 d (7.5)	105.9	C3-Glc
2	3.99 dd (9.0, 8.0)	74.9	C1-Glc-II	4.07 m	75.6	C1, C3-Xyl-II	3.92 t (8.3)	74.5	C1, C2-Xyl-II
3	3.71 t (9.0)	87.8	OMe, C2-Glc-II	4.11 m	77.0	C2, C4-Xyl-II	3.58 t (8.9)	87.6	C2, C4-Xyl-II, OMe
4	4.14 t (9.0)	70.2	C5, C6-Glc-II	4.13 m	70.4		4.07 m	69.9	C5-Xyl-II
5	3.96 m	78.2		4.34 dd (11.4, 4.5) 3.66 dd (11.4, 9.2)	66.9	C1, C3, C4-Xyl-II C3, C4-Xyl-II	4.20 dd (11.3, 5.6) 3.61 dd (11.3, 10.7)	66.9	C1, C3, C4-Xyl-II C1, C3, C4-Xyl-II
6	4.46 dd (11.5, 2.4) 4.27 dd (11.5, 4.9)	62.1	C5-Glc-II						
3-OMe	3.87 s	60.5	C3-Glc-II				3.85 s	60.4	C3-Xyl-II
	Xyl-II								
1	5.39 d (7.0)	105.8	C2-Qui						
2	4.05 dd (8.2, 7.0)	75.5	C1, C3-Xyl-II						
3	4.10 t (8.2)	77.0	C2, C4-Xyl-II						
4	4.13 m	70.5							
5	4.33 dd (11.5, 5.1) 3.66 dd (11.5, 9.7)	66.9	C1, C3, C4-Xyl-II C1, C3, C4-Xyl-II						

^a^ Assignments from 700 MHz ^1^H-^1^H COSY, HSQC, HMBC (8 Hz), and ROESY (270) data.

**Table 3:** Cytotoxicity and selectivity index of triterpene glycosides **1**–**9** from *S. pacificus ^a^*.

Compound	JB6 Cl41	SK-MEL-2		SK-MEL-28		RPMI-7951	
	IC_50_, µM	IC_50_, µM	SI	IC_50_, µM	SI	IC_50_, µM	SI
**1**	6.4 ± 0.05	0.7 ± 0.07	9.1	8.4 ± 0.07	0.8	26.7 ± 0.1	0.24
**2**	31.5 ± 4.1	37.6 ± 0.2	0.8	29.0 ± 0.02	1.0	36.4 ± 0.2	0.87
**3**	6.0 ± 0.1	0.68 ± 0.06	8.8	14.8 ± 0.03	0.4	27.9 ± 0.09	0.22
**4**	6.1 ± 0.4	0.67 ± 0.04	9.1	8.3 ± 0.1	1.4	29.2 ± 0.04	0.2
**5**	8.7 ± 0.2	37.3 ± 0.01	0.2	25.3 ± 0.09	0.3	38.0 ± 0.3	0.23
**6**	6.6 ± 0.3	0.69 ± 0.03	9.5	8.2 ± 0.2	0.8	32.4 ± 0.5	0.2
**7**	8.7 ± 0.07	5.6 ± 0.1	1.4	23.0 ± 0.07	0.3	31.4 ± 0.1	0.25
**8**	32.8 ± 2.8	>50	n.d.	42.0 ± 0.4	0.8	>50	n.d.
**9**	7.4 ± 0.8	0.75 ± 0.03	9.9	23.3 ± 0.6	0.32	32.4 ± 0.08	0.23

*^a^* IC_50_—the concentration of compounds that caused a 50% reduction in cell viability of tested normal and cancer cells. Values are indicated as mean ± standard deviation. SI—the selectivity index was calculated by the following equation: SI = IC_50_ value against non-cancerous cells/IC_50_ value against cancer cells. n.d.—not detected.

**Table 4:** Hemolytic activity of triterpene glycosides **1**–**9** from *S. pacificus ^a^*.

Compound	Human erythrocytes (O(I)+)
	ED_50_, µM
Cucumarioside A_2_-2	0.95 ± 0.04
**1**	2.03 ± 0.19
**2**	4.08 ± 0.36
**3**	0.72 ± 0.05
**4**	2.48 ± 0.05
**5**	7.02 ± 0.46
**6**	1.68 ± 0.14
**7**	4.07 ± 0.10
**8**	6.16 ± 0.05
**9**	2.85 ± 0.14

*^a^* ED_50_—The effective dose causing 50% of erythrocytes hemolysis was calculated using the computer program SigmaPlot 10.0. All experiments were made in triplicate, *p* < 0.01.

## Data Availability

The data presented in this study are available on request from the corresponding authors.

## Supplementary Materials

The following supporting information can be downloaded at: https://www.mdpi.com/article/10.3390/md20030216/s1. Figure S1: HRESIMS spectrum of pacificusoside D (**1**); Figure S2: IR spectrum of pacificusoside D (**1**) in KBr; Figure S3: ^1^H-NMR spectrum of pacificusoside D (**1**) in C_5_D_5_N; Figures S4–S6: Expansion №1, №2, and №3 of 1H-NMR spectrum of pacificusoside D (1) in C5D5N Figure S7: ^13^C-NMR spectrum of pacificusoside D (**1**) in C_5_D_5_N; Figure S5: ^1^H-^1^H COSY spectrum of pacificusoside D (**1**) in C_5_D_5_N; Figure S6: HSQC spectrum of pacificusoside D (**1**) in C_5_D_5_N; Figure S7: HMBC spectrum of pacificusoside D (**1**) in C_5_D_5_N; Figures S8–S10: Expansion №1, №2, and №3 of 13C-NMR spectrum of pacificusoside D (1) in C5D5N; Figure S11: ^1^H-^1^H COSY spectrum of pacificusoside D (**1**) in C5D5N; Figure S12: HSQC spectrum of pacificusoside D (1) in C5D5N; Figure S13: HMBC spectrum of pacificusoside D (1) in C5D5N; Figure S14: ROESY spectrum of pacificusoside D (**1**) in C_5_D_5_N; Figure S15: UV spectrum of pacificusoside D (**1**) in MeOH; Figure S16: HRESIMS spectrum of pacificusoside E (**2**); Figure S17: IR spectrum of pacificusoside E (**2**) in KBr; Figure S18: ^1^H-NMR spectrum of pacificusoside E (**2**) in C_5_D_5_N; Figure S19: ^13^C-NMR spectrum of pacificusoside E (**2**) in C_5_D_5_N; Figure S20: ^1^H-^1^H COSY spectrum of pacificusoside E (**2**) in C_5_D_5_N; Figure S21: HSQC spectrum of pacificusoside E (**2**) in C_5_D_5_N; Figure S22: HMBC spectrum of pacificusoside E (**2**) in C_5_D_5_N; Figure S23: ROESY spectrum of pacificusoside E (**2**) in C_5_D_5_N; Figure S24: HRESIMS spectrum of pacificusoside F (**3**); Figure S25: IR spectrum of pacificusoside F (**3**) in KBr; Figure S26: ^1^H-NMR spectrum of pacificusoside F (**3**) in C_5_D_5_N; Figure S27: ^13^C-NMR spectrum of pacificusoside F (**3**) in C_5_D_5_N; Figure S28: ^1^H-^1^H COSY spectrum of pacificusoside F (**3**) in C_5_D_5_N; Figure S29: HSQC spectrum of pacificusoside F (**3**) in C_5_D_5_N; Figure S30: HMBC spectrum of pacificusoside F (**3**) in C_5_D_5_N; Figure S31: ROESY spectrum of pacificusoside F (**3**) in C_5_D_5_N; Figure S32: HRESIMS spectrum of pacificusoside G (**5**); Figure S33: IR spectrum of pacificusoside G (**5**) in KBr; Figure S34: ^1^H-NMR spectrum of pacificusoside G (**5**) in C_5_D_5_N; Figures S35–S37: Expansion №1, №2, №3 of ^1^H-NMR spectrum of pacificusoside G (**5**) in C5D5N; Figure S38: ^13^C-NMR spectrum of pacificusoside G (**5**) in C_5_D_5_N; Figures S39–S41: Expansion №1, №2, №3 of ^13^C-NMR spectrum of pacificusoside G (**5**) in C5D5N; Figure S42: UV spectrum of pacificusoside G (**5**) in MeOH; Figure S43: ^1^H-^1^H COSY spectrum of pacificusoside G (**5**) in C_5_D_5_N; Figure S44: HSQC spectrum of pacificusoside G (**5**) in C_5_D_5_N; Figure S45: HMBC spectrum of pacificusoside G (**5**) in C_5_D_5_N; Figure S46: ROESY spectrum of pacificusoside G (**5**) in C_5_D_5_N; Figure S47: HRESIMS spectrum of pacificusoside H (**6**); Figure S48: IR spectrum of pacificusoside H (**6**) in KBr; Figure S49: ^1^H-NMR spectrum of pacificusoside H (**6**) in C_5_D_5_N; Figure S50: ^13^C-NMR spectrum of pacificusoside H (**6**) in C_5_D_5_N; Figure S51: ^1^H-^1^H COSY spectrum of pacificusoside H (**6**) in C_5_D_5_N; Figure S52: HSQC spectrum of pacificusoside H (**6**) in C_5_D_5_N; Figure S53: HMBC spectrum of pacificusoside H (**6**) in C_5_D_5_N; Figure S54: ROESY spectrum of pacificusoside H (**6**) in C_5_D_5_N; Figure S55: HRESIMS spectrum of pacificusoside I (**7**); Figure S56: IR spectrum of pacificusoside I (**7**) in KBr; Figure S57: ^1^H-NMR spectrum of pacificusoside I (**7**) in C_5_D_5_N; Figure S58: ^13^C-NMR spectrum of pacificusoside I (**7**) in C_5_D_5_N; Figure S59: ^1^H-^1^H COSY spectrum of pacificusoside I (**7**) in C_5_D_5_N; Figure S60: HSQC spectrum of pacificusoside I (**7**) in C_5_D_5_N; Figure S61: HMBC spectrum of pacificusoside I (**7**) in C_5_D_5_N; Figure S62: ROESY spectrum of pacificusoside I (**7**) in C_5_D_5_N; Figure S63: HRESIMS spectrum of pacificusoside J (**8**); Figure S64: IR spectrum of pacificusoside J (**8**) in KBr; Figure S65: ^1^H-NMR spectrum of pacificusoside J (**8**) in C_5_D_5_N; Figure S66: ^13^C-NMR spectrum of pacificusoside J (**8**) in C_5_D_5_N; Figure S67: ^1^H-^1^H COSY spectrum of pacificusoside J (**8**) in C_5_D_5_N; Figure S68: HSQC spectrum of pacificusoside J (**8**) in C_5_D_5_N; Figure S69: HMBC spectrum of pacificusoside J (**8**) in C_5_D_5_N; Figure S70: ROESY spectrum of pacificusoside J (**8**) in C_5_D_5_N; Figure S71: HRESIMS spectrum of pacificusoside K (**9**); Figure S72: IR spectrum of pacificusoside K (**9**) in KBr; Figure S73: ^1^H-NMR spectrum of pacificusoside K (**9**) in C_5_D_5_N; Figures S74–S76: Expansion №1, №2, №3 of ^1^H-NMR spectrum of pacificusoside K (**9**) in C5D5N. Figure S77: ^13^C-NMR spectrum of pacificusoside K (**9**) in C_5_D_5_N; Figures S78–S80: Expansion №1, №2, №3 of ^13^C-NMR spectrum of pacificusoside K (**9**) in C5D5N; Figure S81: ^1^H-^1^H COSY spectrum of pacificusoside K (**9**) in C_5_D_5_N; Figure S82: HSQC spectrum of pacificusoside K (**9**) in C_5_D_5_N; Figure S83: HMBC spectrum of pacificusoside K (**9**) in C_5_D_5_N; Figure S84: ROESY spectrum of pacificusoside K (**9**) in C_5_D_5_N.
