# Supplementary material for: In Vitro Anticancer and Cancer-Preventive Activity of New Triterpene Glycosides from the Far Eastern Starfish Solaster pacificus
Source: Mar Drugs. 2022 Mar 20;20(3):216. doi: 10.3390/md20030216 (PMC8951750; doi:10.3390/md20030216)

Supplementary Materials

In Vitro Anticancer and Cancer-Preventive Activity of New Triterpene Glycosides from the Far Eastern Starfish *Solaster pacificus*

Timofey V. Malyarenko[1,2*], Olesya S. Malyarenko[1], Alla A. Kicha[1], Anatoly I. Kalinovsky[1], Pavel S. Dmitrenok[1], and Natalia V. Ivanchina[1*]

[1] G.B. Elyakov Pacific Institute of Bioorganic Chemistry, Far Eastern Branch of the Russian Academy of Sciences, Pr. 100-let Vladivostoku 159, 690022 Vladivostok, Russia; malyarenko.os@gmail.com (O.S.M.); kicha@piboc.dvo.ru (A.A.K.); kaaniw@piboc.dvo.ru (A.I.K.); paveldmt@piboc.dvo.ru (P.S.D.)

[2] Department of Bioorganic Chemistry and Biotechnology, School of Natural Sciences, Far Eastern Federal University, Russky Island, Ajax Bay, 10, 690922, Vladivostok, Russia

* Correspondence: malyarenko-tv@mail.ru (T.V.M.); ivanchina@piboc.dvo.ru (N.V.I.). Tel.: +7-423-2312-360; Fax: +7-423-2314-050

List

Figure S1. HRESIMS spectrum of pacificusoside D (**1**).

Figure S2. IR spectrum of pacificusoside D (**1**) in KBr.

Figure S3. ^{1}H-NMR spectrum of pacificusoside D (**1**) in C_5D_5N.

Figure S4. Expansion №1 of ^{1}H-NMR spectrum of pacificusoside D (**1**) in C_5D_5N.

Figure S5. Expansion №2 of ^{1}H-NMR spectrum of pacificusoside D (**1**) in C_5D_5N.

Figure S6. Expansion №3 of ^{1}H-NMR spectrum of pacificusoside D (**1**) in C_5D_5N.

Figure S7. ^{13}C-NMR spectrum of pacificusoside D (**1**) in C_5D_5N.

Figure S8. Expansion №1 of ^{13}C-NMR spectrum of pacificusoside D (**1**) in C_5D_5N.

Figure S9. Expansion №2 of ^{13}C-NMR spectrum of pacificusoside D (**1**) in C_5D_5N.

Figure S10. Expansion №3 of ^{13}C-NMR spectrum of pacificusoside D (**1**) in C_5D_5N.

Figure S11. ^{1}H-^{1}H COSY spectrum of pacificusoside D (**1**) in C_5D_5N.

Figure S12. HSQC spectrum of pacificusoside D (**1**) in C_5D_5N.

Figure S13. HMBC spectrum of pacificusoside D (**1**) in C_5D_5N.

Figure S14. ROESY spectrum of pacificusoside D (**1**) in C_5D_5N.

Figure S15. UV spectrum of pacificusoside D (**1**) in MeOH.

Figure S16. HRESIMS spectrum of pacificusoside E (**2**).

Figure S17. IR spectrum of pacificusoside E (**2**) in KBr.

Figure S18. ^{1}H-NMR spectrum of pacificusoside E (**2**) in C_5D_5N.

Figure S19. ^{13}C-NMR spectrum of pacificusoside E (**2**) in C_5D_5N.

Figure S20. ^{1}H-^{1}H COSY spectrum of pacificusoside E (**2**) in C_5D_5N.

Figure S21. HSQC spectrum of pacificusoside E (**2**) in C_5D_5N.

Figure S22. HMBC spectrum of pacificusoside E (**2**) in C_5D_5N.

Figure S23. ROESY spectrum of pacificusoside E (**2**) in C_5D_5N.

Figure S24. HRESIMS spectrum of pacificusoside F (**3**).

Figure S25. IR spectrum of pacificusoside F (**3**) in KBr.

Figure S26. ^{1}H-NMR spectrum of pacificusoside F (**3**) in C_5D_5N.

Figure S27. ^{13}C-NMR spectrum of pacificusoside F (**3**) in C_5D_5N.

Figure S28. ^{1}H-^{1}H COSY spectrum of pacificusoside F (**3**) in C_5D_5N.

Figure S29. HSQC spectrum of pacificusoside F (**3**) in C_5D_5N.

Figure S30. HMBC spectrum of pacificusoside F (**3**) in C_5D_5N.

Figure S31. ROESY spectrum of pacificusoside F (**3**) in C_5D_5N.

Figure S32. HRESIMS spectrum of pacificusoside G (**5**).

Figure S33. IR spectrum of pacificusoside G (**5**) in KBr.

Figure S34. ^{1}H-NMR spectrum of pacificusoside G (**5**) in C_5D_5N.

Figure S35. Expansion №1 of ^{1}H-NMR spectrum of pacificusoside G (**5**) in C_5D_5N.

Figure S36. Expansion №2 of ^{1}H-NMR spectrum of pacificusoside G (**5**) in C_5D_5N.

Figure S37. Expansion №3 of ^{1}H-NMR spectrum of pacificusoside G (**5**) in C_5D_5N.

Figure S38. ^{13}C-NMR spectrum of pacificusoside G (**5**) in C_5D_5N.

Figure S39. Expansion №1 of ^{13}C-NMR spectrum of pacificusoside G (**5**) in C_5D_5N.

Figure S40. Expansion №2 of ^{13}C-NMR spectrum of pacificusoside G (**5**) in C_5D_5N.

Figure S41. Expansion №3 of ^{13}C-NMR spectrum of pacificusoside G (**5**) in C_5D_5N.

Figure S42. UV spectrum of pacificusoside G (**5**) in MeOH.

Figure S43. ^{1}H-^{1}H COSY spectrum of pacificusoside G (**5**) in C_5D_5N.

Figure S44. HSQC spectrum of pacificusoside G (**5**) in C_5D_5N.

Figure S45. HMBC spectrum of pacificusoside G (**5**) in C_5D_5N.

Figure S46. ROESY spectrum of pacificusoside G (**5**) in C_5D_5N.

Figure S47. HRESIMS spectrum of pacificusoside H (**6**).

Figure S48. IR spectrum of pacificusoside H (**6**) in KBr.

Figure S49. ^1H-NMR spectrum of pacificusoside H (**6**) in C_5D_5N.

Figure S50. ^{13}C-NMR spectrum of pacificusoside H (**6**) in C_5D_5N.

Figure S51. ^1H-^1H COSY spectrum of pacificusoside H (**6**) in C_5D_5N.

Figure S52. HSQC spectrum of pacificusoside H (**6**) in C_5D_5N.

Figure S53. HMBC spectrum of pacificusoside H (**6**) in C_5D_5N.

Figure S54. ROESY spectrum of pacificusoside H (**6**) in C_5D_5N.

Figure S55. HRESIMS spectrum of pacificusoside I (**7**).

Figure S56. IR spectrum of pacificusoside I (**7**) in KBr.

Figure S57. ^1H-NMR spectrum of pacificusoside I (**7**) in C_5D_5N.

Figure S58. ^{13}C-NMR spectrum of pacificusoside I (**7**) in C_5D_5N.

Figure S59. ^1H-^1H COSY spectrum of pacificusoside I (**7**) in C_5D_5N.

Figure S60. HSQC spectrum of pacificusoside I (**7**) in C_5D_5N.

Figure S61. HMBC spectrum of pacificusoside I (**7**) in C_5D_5N.

Figure S62. ROESY spectrum of pacificusoside I (**7**) in C_5D_5N.

Figure S63. HRESIMS spectrum of pacificusoside J (**8**).

Figure S64. IR spectrum of pacificusoside J (**8**) in KBr.

Figure S65. ^1H-NMR spectrum of pacificusoside J (**8**) in C_5D_5N.

Figure S66. ^{13}C-NMR spectrum of pacificusoside J (**8**) in C_5D_5N.

Figure S67. ^{1}H-^{1}H COSY spectrum of pacificusoside J (**8**) in C_5D_5N.

Figure S68. HSQC spectrum of pacificusoside J (**8**) in C_5D_5N.

Figure S69. HMBC spectrum of pacificusoside J (**8**) in C_5D_5N.

Figure S70. ROESY spectrum of pacificusoside J (**8**) in C_5D_5N.

Figure S71. HRESIMS spectrum of pacificusoside K (**9**).

Figure S72. IR spectrum of pacificusoside K (**9**) in KBr.

Figure S73. ^{1}H-NMR spectrum of pacificusoside K (**9**) in C_5D_5N.

Figure S74. Expansion №1 of ^{1}H-NMR spectrum of pacificusoside K (**9**) in C_5D_5N.

Figure S75. Expansion №2 of ^{1}H-NMR spectrum of pacificusoside K (**9**) in C_5D_5N.

Figure S76. Expansion №3 of ^{1}H-NMR spectrum of pacificusoside K (**9**) in C_5D_5N.

Figure S77. ^{13}C-NMR spectrum of pacificusoside K (**9**) in C_5D_5N.

Figure S78. Expansion №1 of ^{13}C-NMR spectrum of pacificusoside K (**9**) in C_5D_5N.

Figure S79. Expansion №2 of ^{13}C-NMR spectrum of pacificusoside K (**9**) in C_5D_5N.

Figure S80. Expansion №3 of ^{13}C-NMR spectrum of pacificusoside K (**9**) in C_5D_5N.

Figure S81. ^{1}H-^{1}H COSY spectrum of pacificusoside K (**9**) in C_5D_5N.

Figure S82. HSQC spectrum of pacificusoside K (**9**) in C_5D_5N.

Figure S83. HMBC spectrum of pacificusoside K (**9**) in C_5D_5N.

Figure S84. ROESY spectrum of pacificusoside K (**9**) in C_5D_5N.

Figure S1. HRESIMS spectrum of pacificusoside D (**1**).

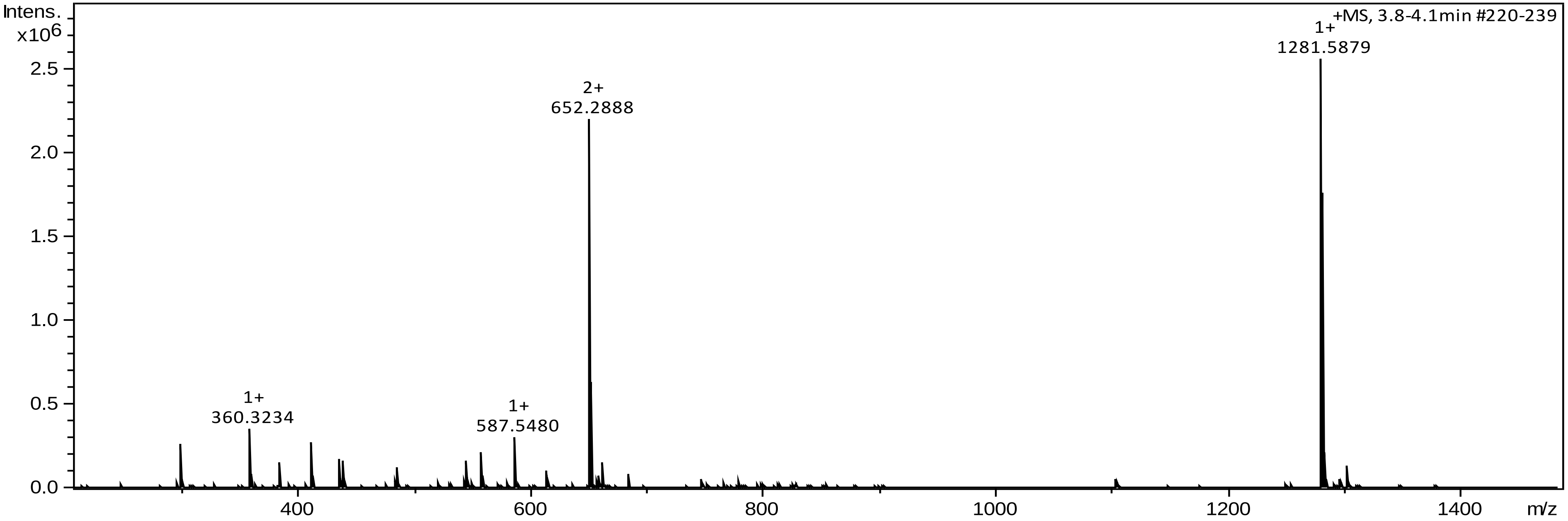

Figure S2. IR spectrum of pacificusoside D (**1**) in KBr.

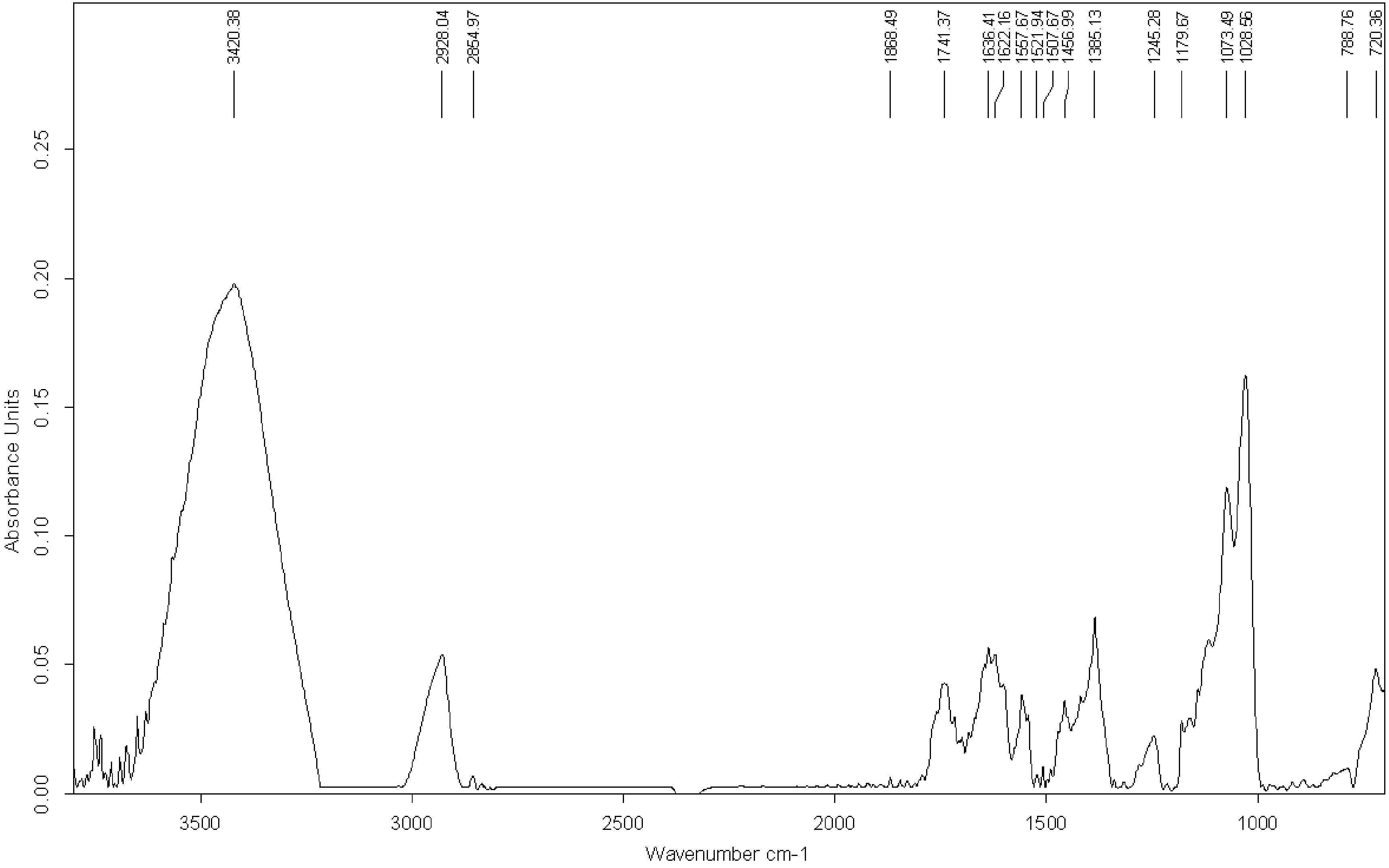

Figure S3. ^{1}H-NMR spectrum of pacificusoside D (**1**) in C_5D_5N.

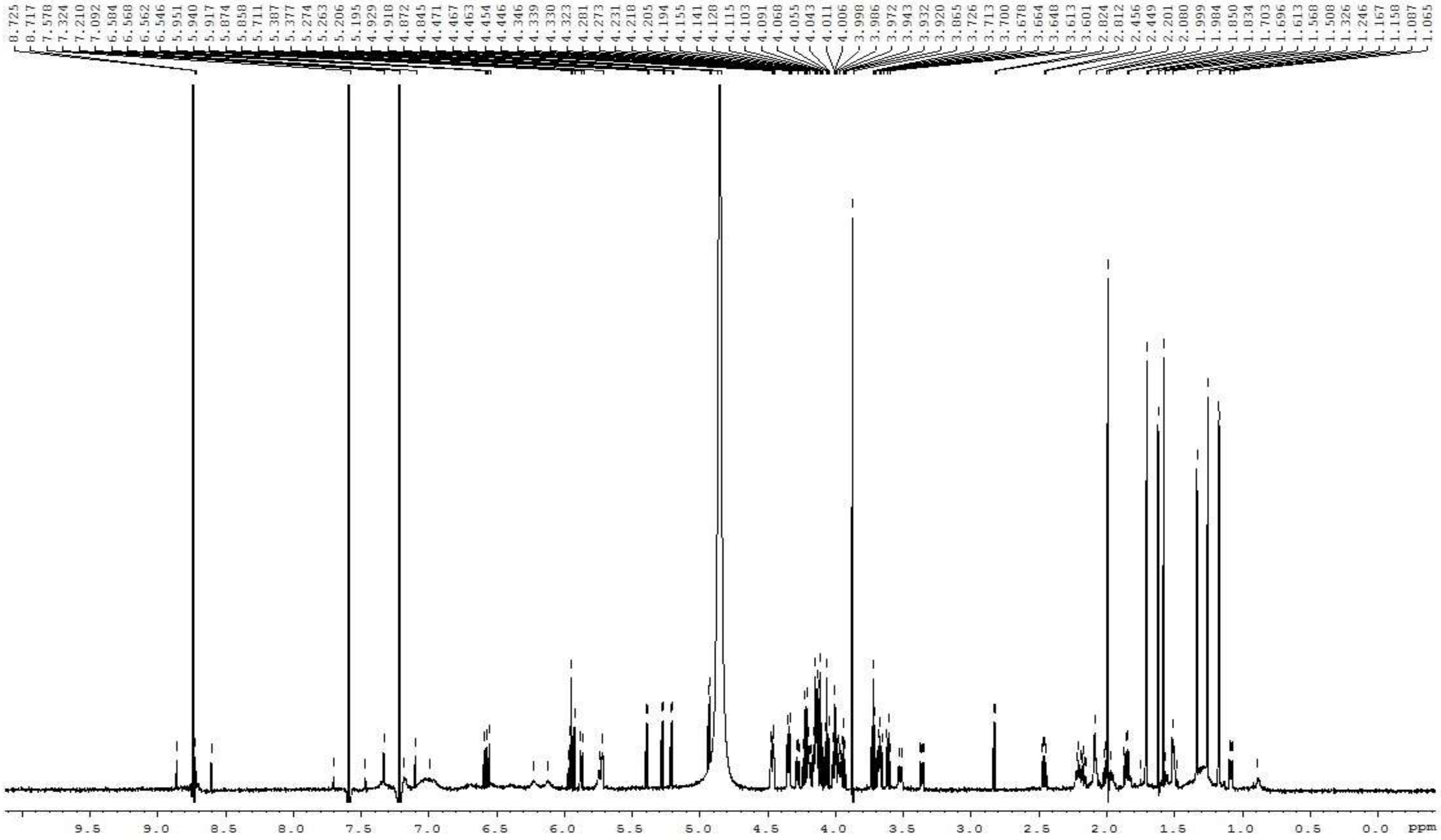

Figure S4. Expansion №1 of ^{1}H-NMR spectrum of pacificusoside D (**1**) in C_5D_5N.

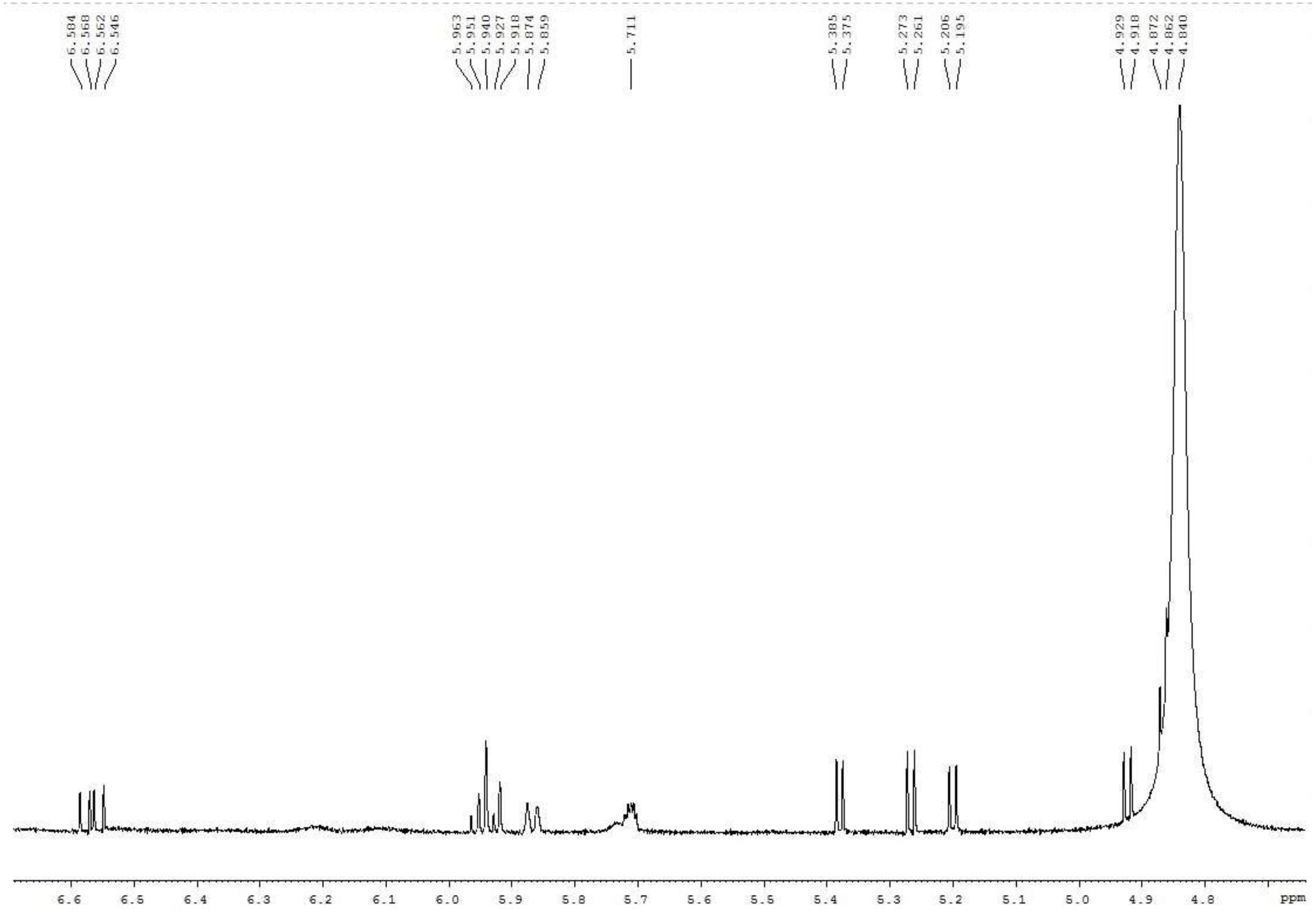

Figure S5. Expansion №2 of ^{1}H-NMR spectrum of pacificusoside D (**1**) in C_5D_5N.

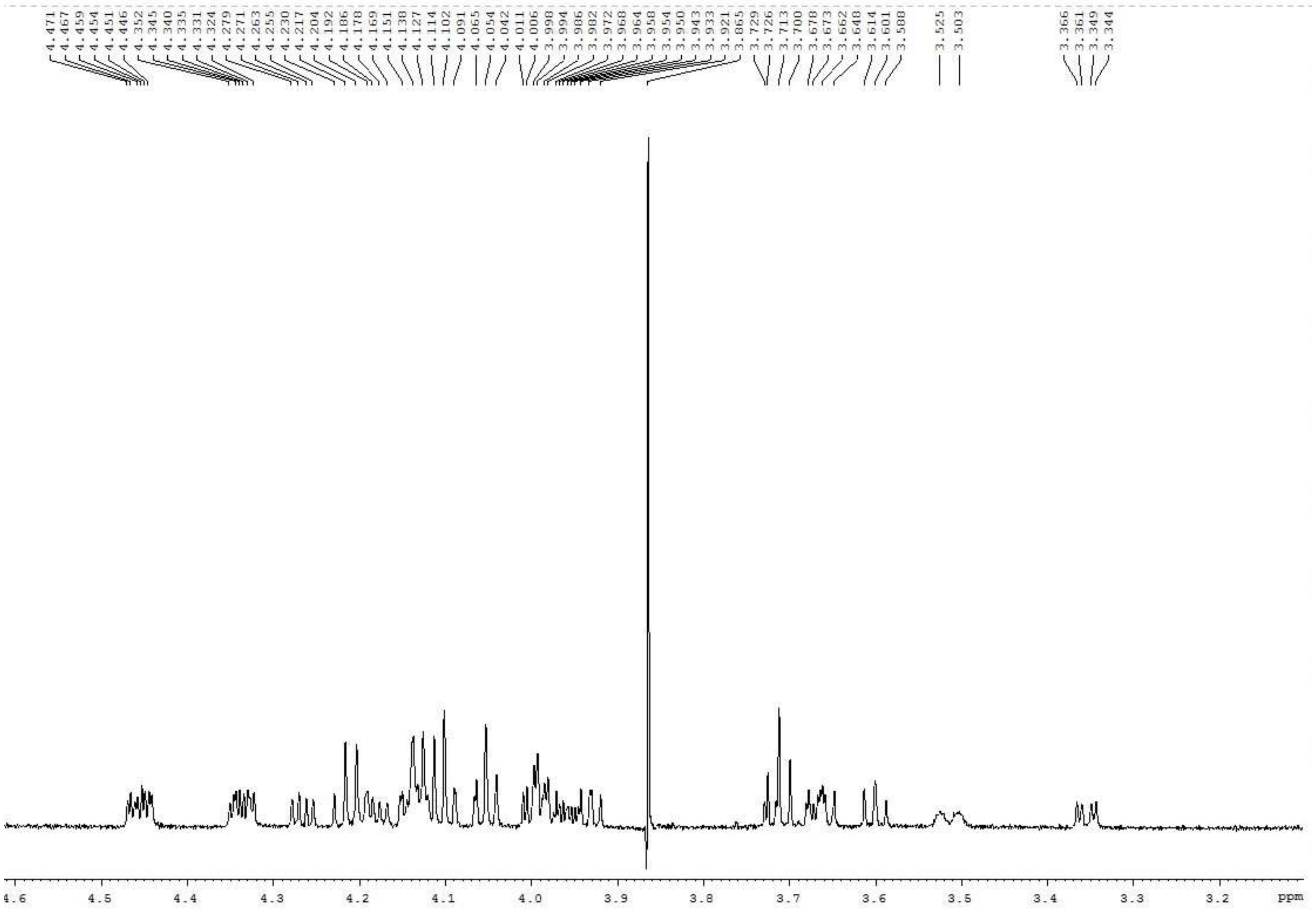

Figure S6. Expansion №3 of ^{1}H-NMR spectrum of pacificusoside D (**1**) in C_5D_5N.

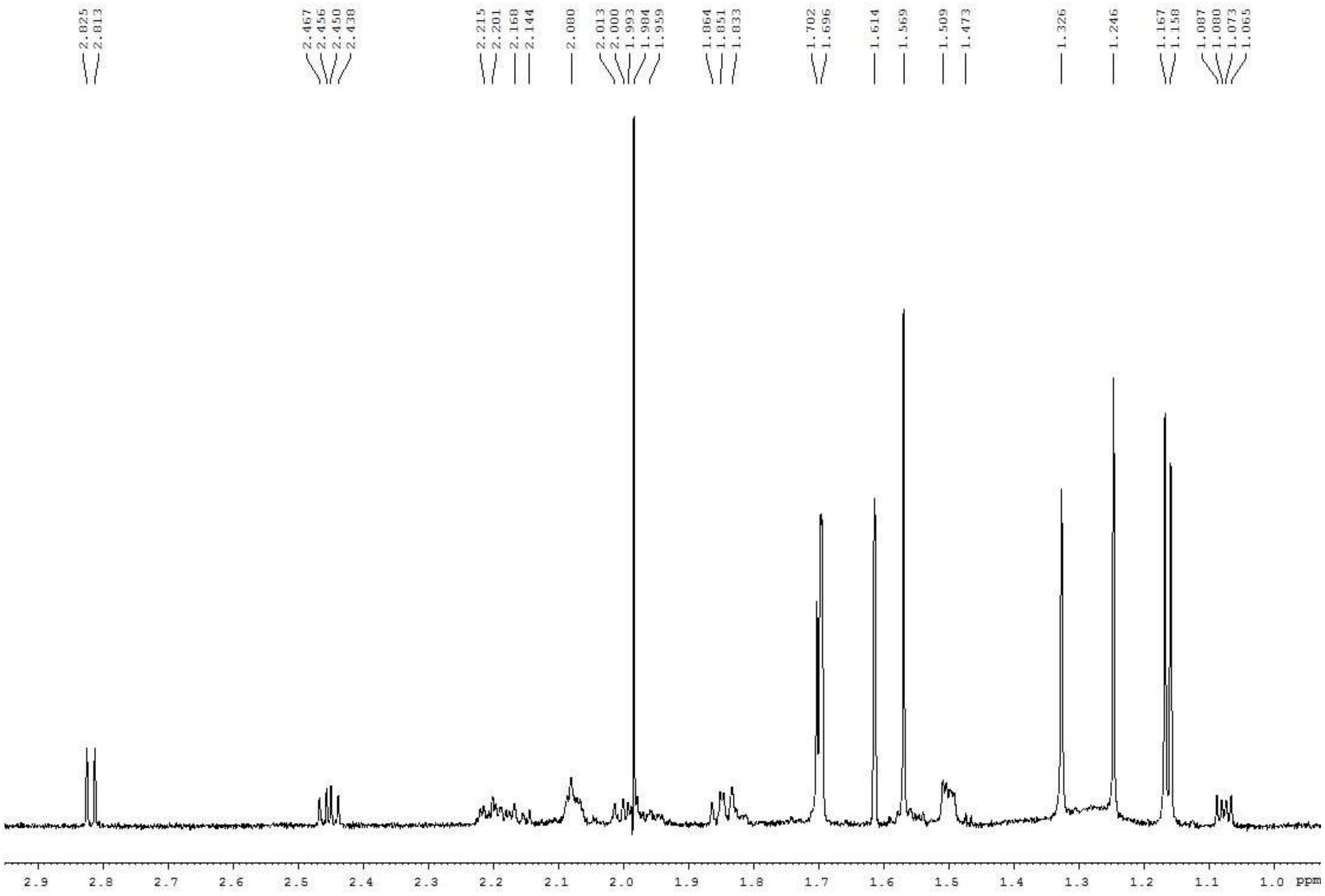

Figure S7. ^{13}C-NMR spectrum of pacificusoside D (**1**) in C_5D_5N.

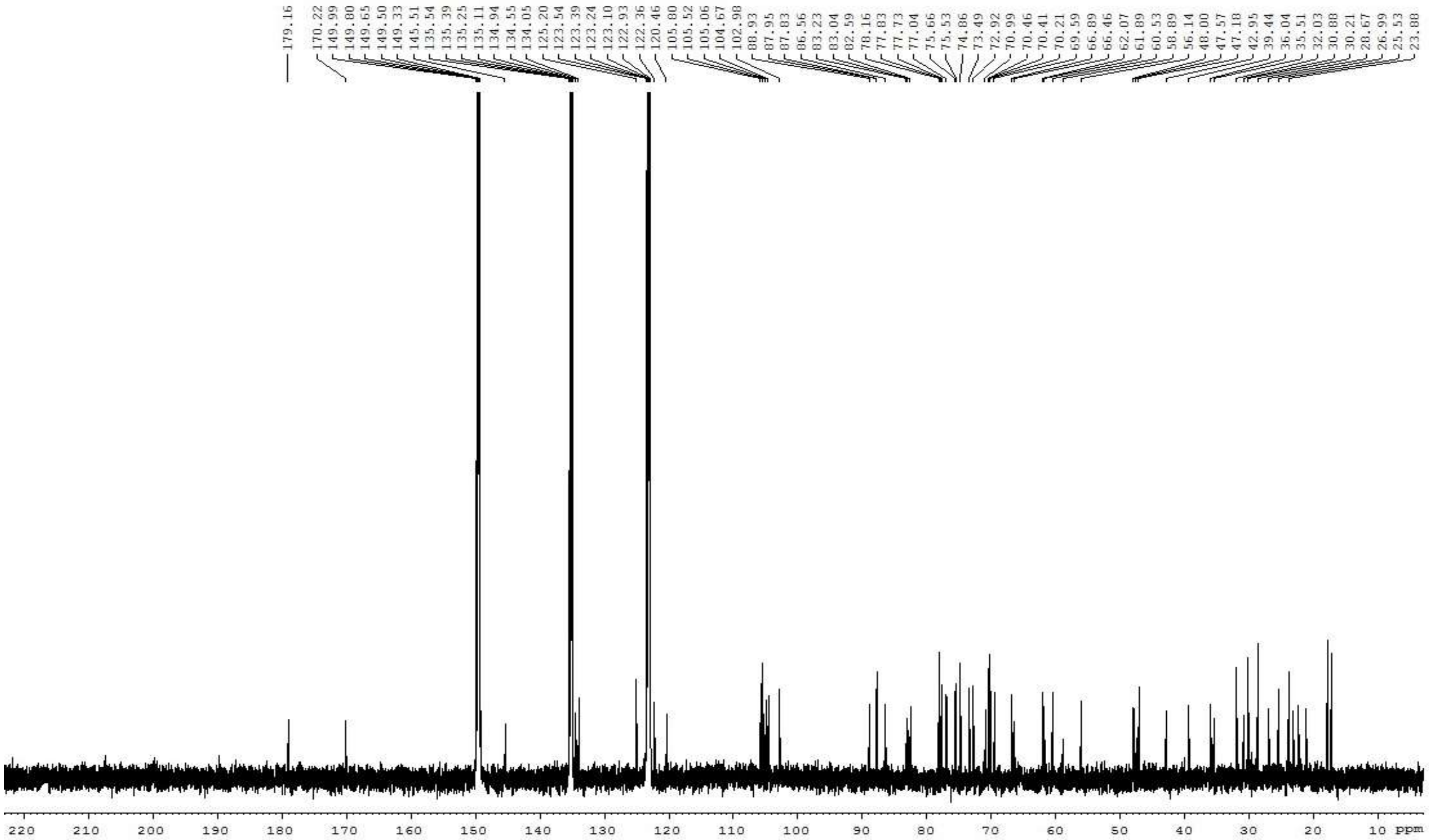

Figure S8. Expansion №1 of ^{13}C-NMR spectrum of pacificusoside D (**1**) in C_5D_5N.

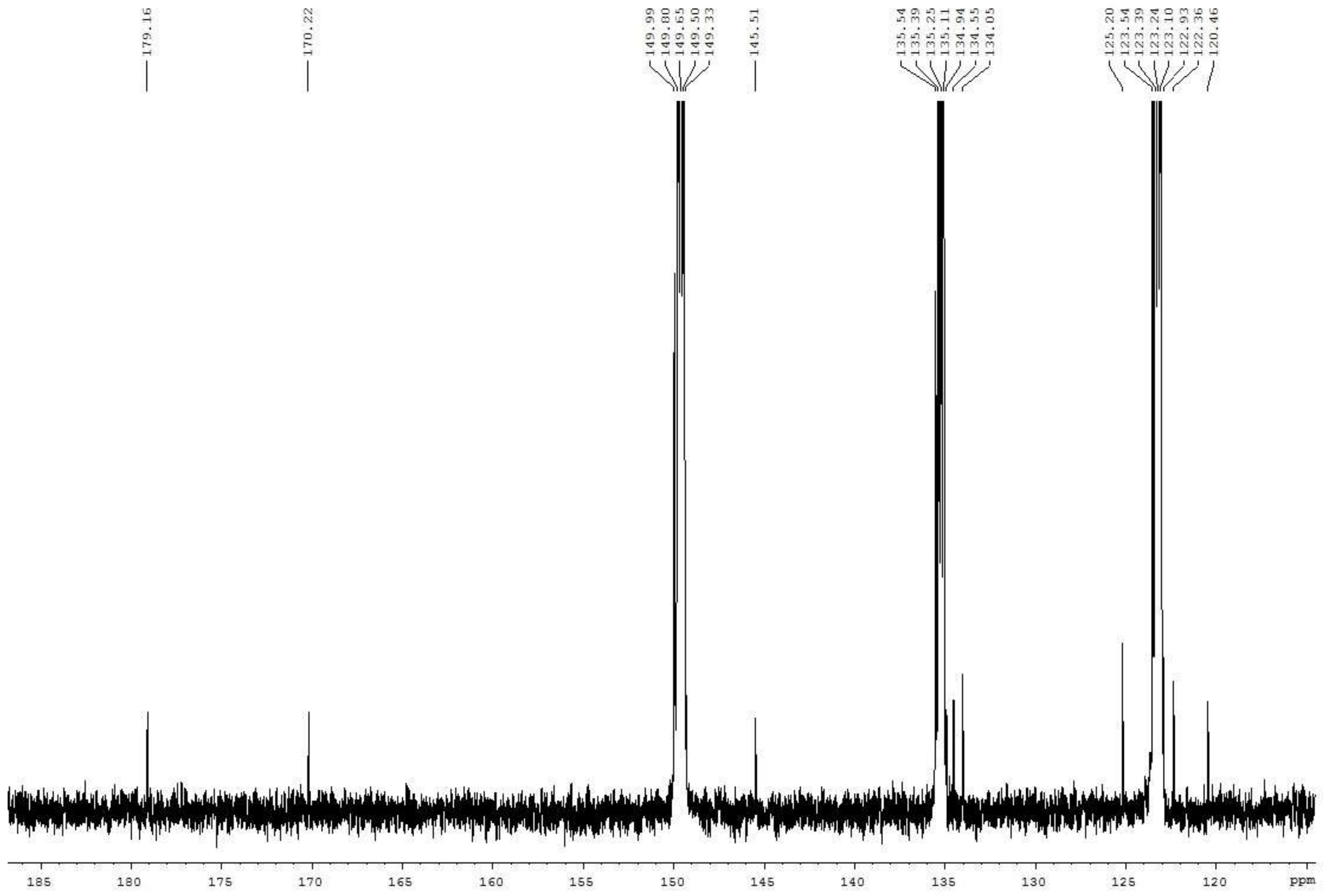

Figure S9. Expansion №2 of ^{13}C-NMR spectrum of pacificusoside D (**1**) in C_5D_5N.

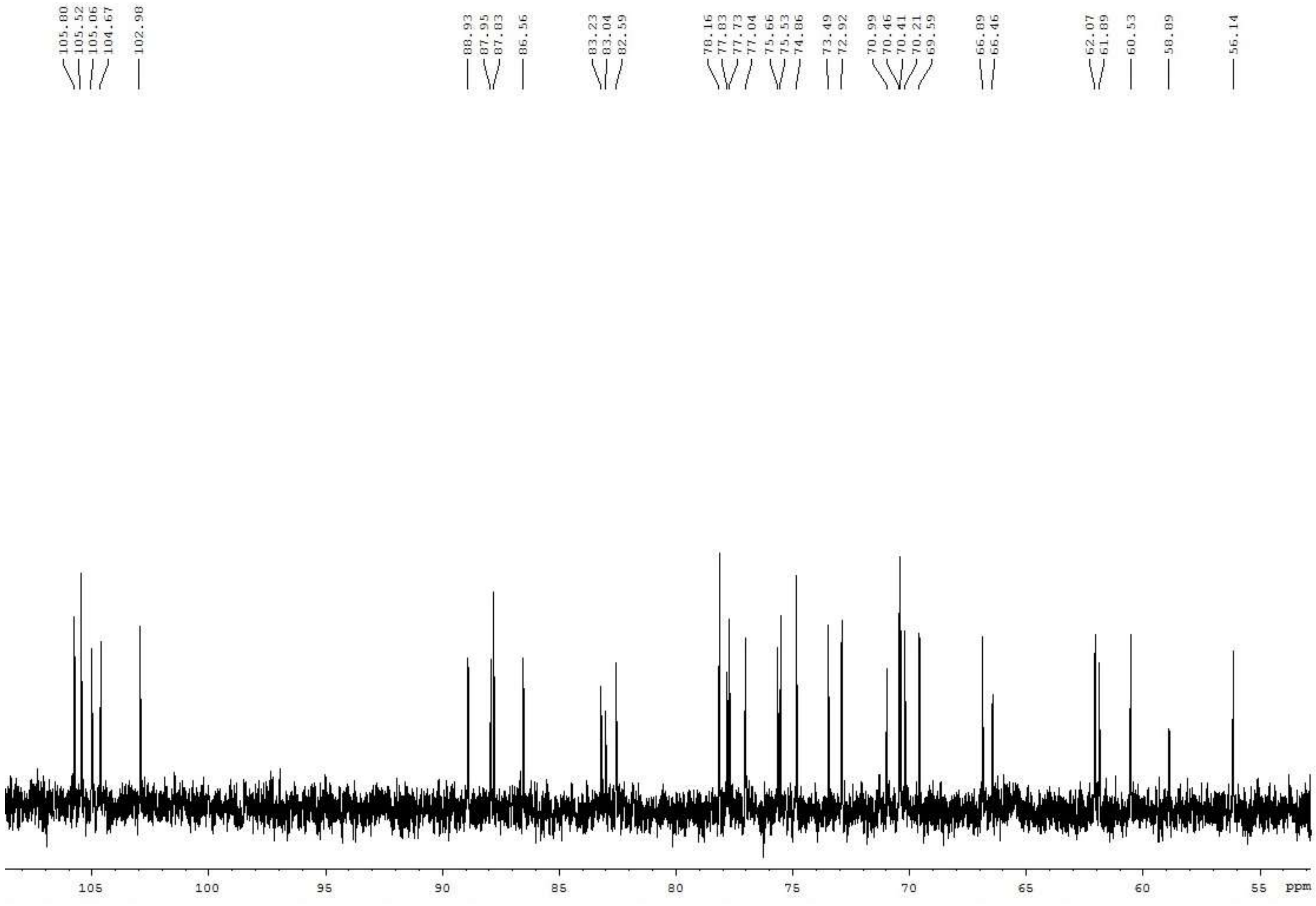

Figure S10. Expansion №3 of ^{13}C-NMR spectrum of pacificusoside D (**1**) in C_5D_5N.

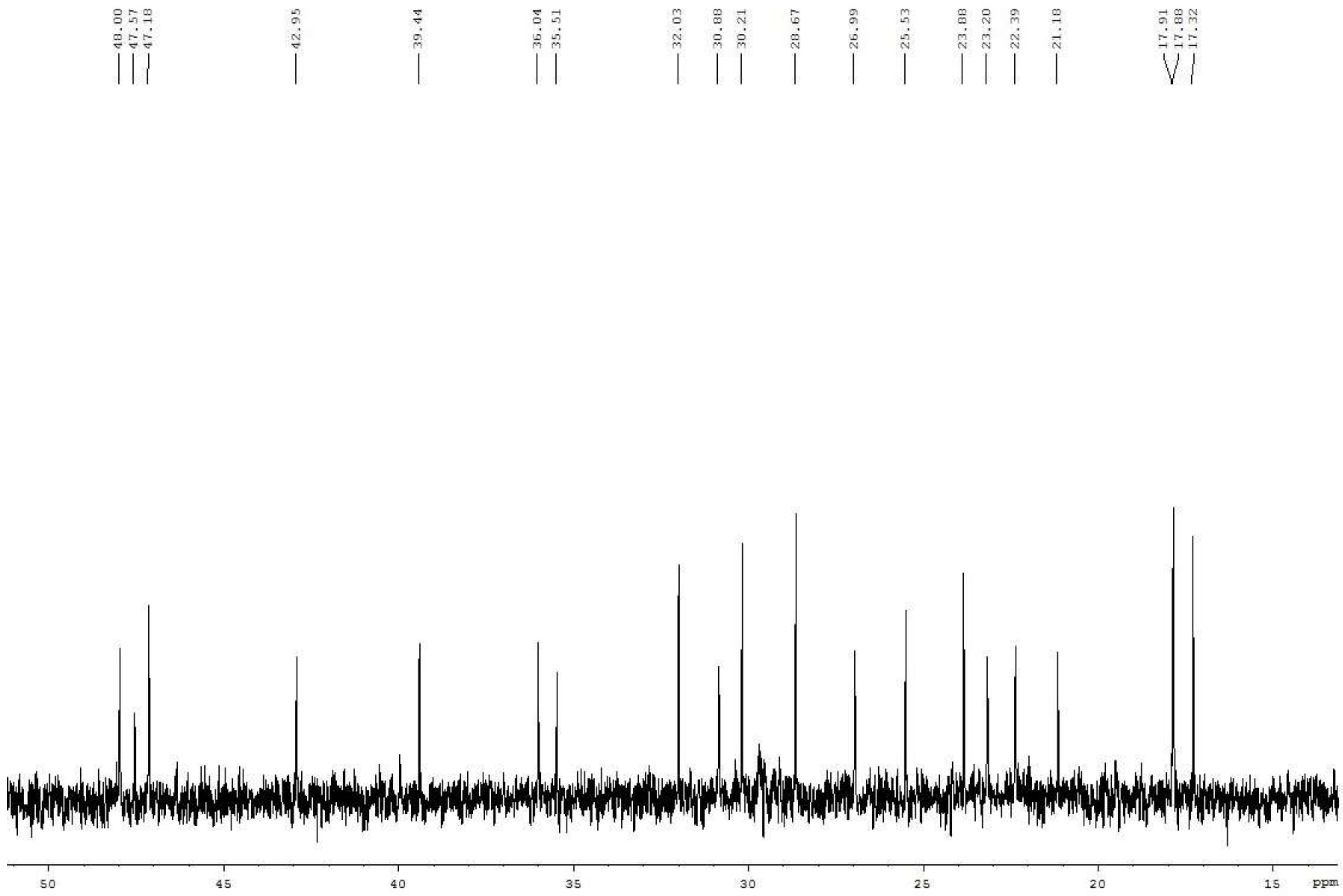

Figure S11. ^1H-^1H COSY spectrum of pacificusoside D (**1**) in C_5D_5N.

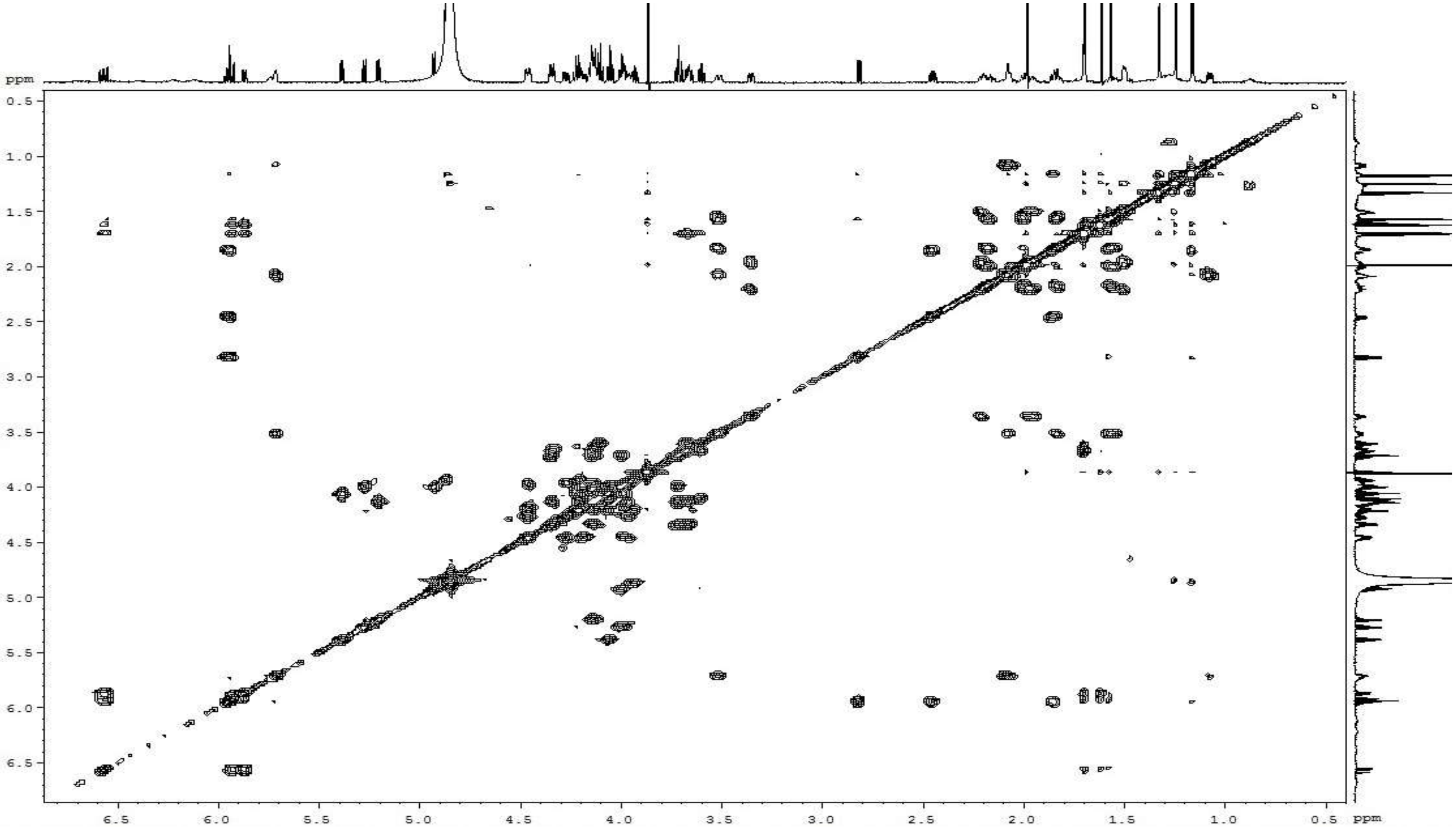

Figure S12. HSQC spectrum of pacificusoside D (**1**) in C_5D_5N.

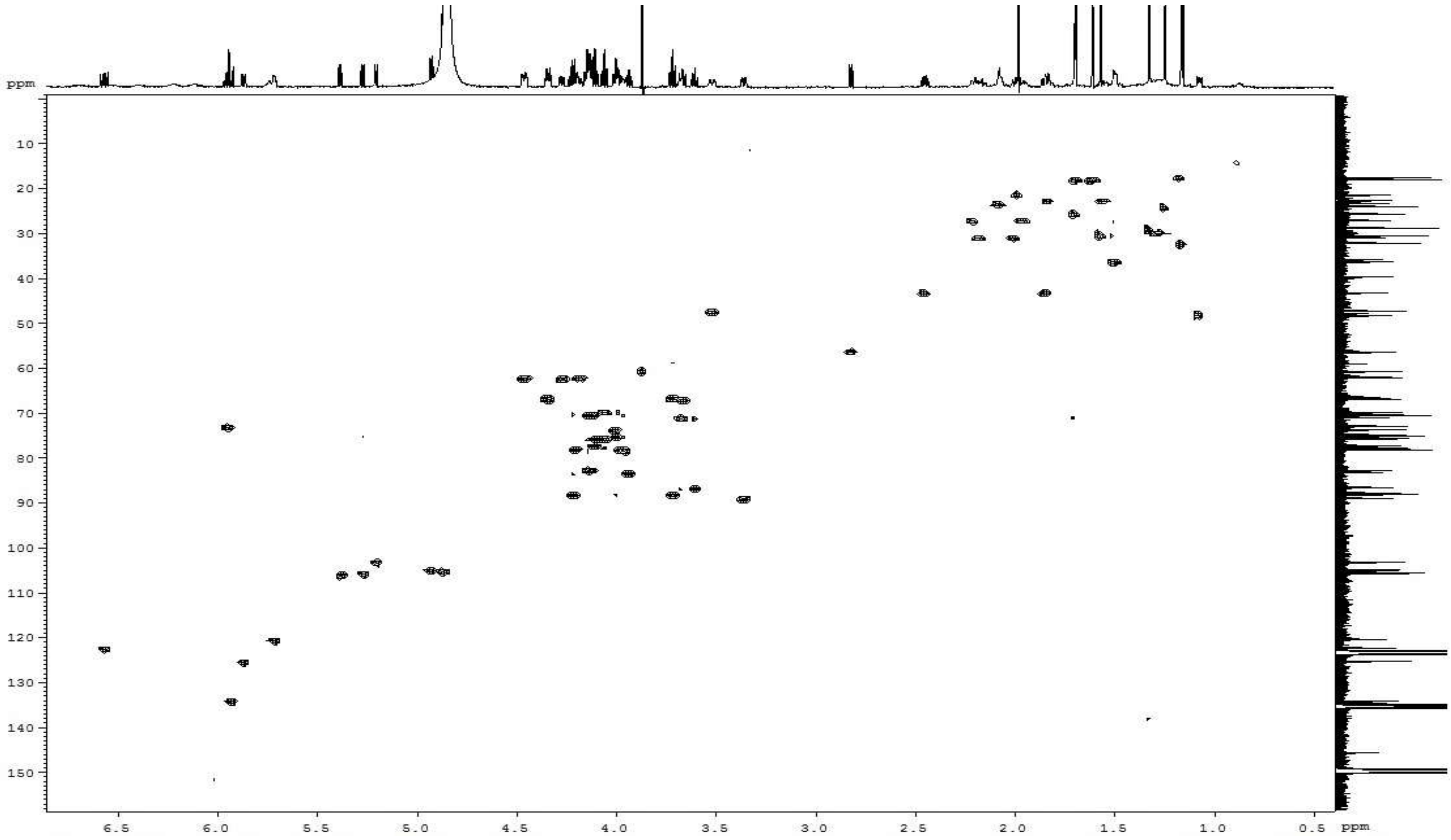

Figure S13. HMBC spectrum of pacificusoside D (**1**) in C_5D_5N.

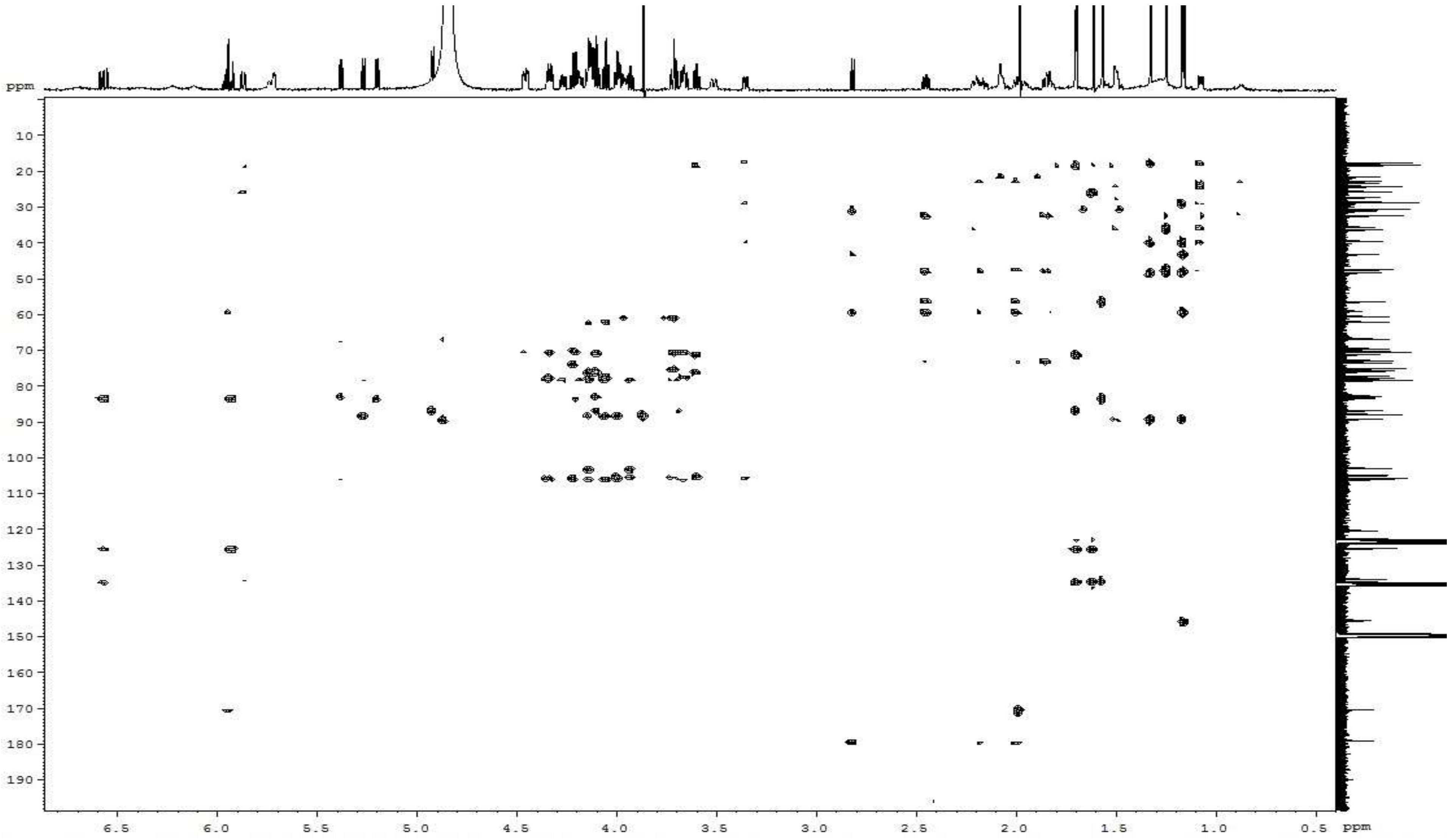

Figure S14. ROESY spectrum of pacificusoside D (**1**) in C_5D_5N.

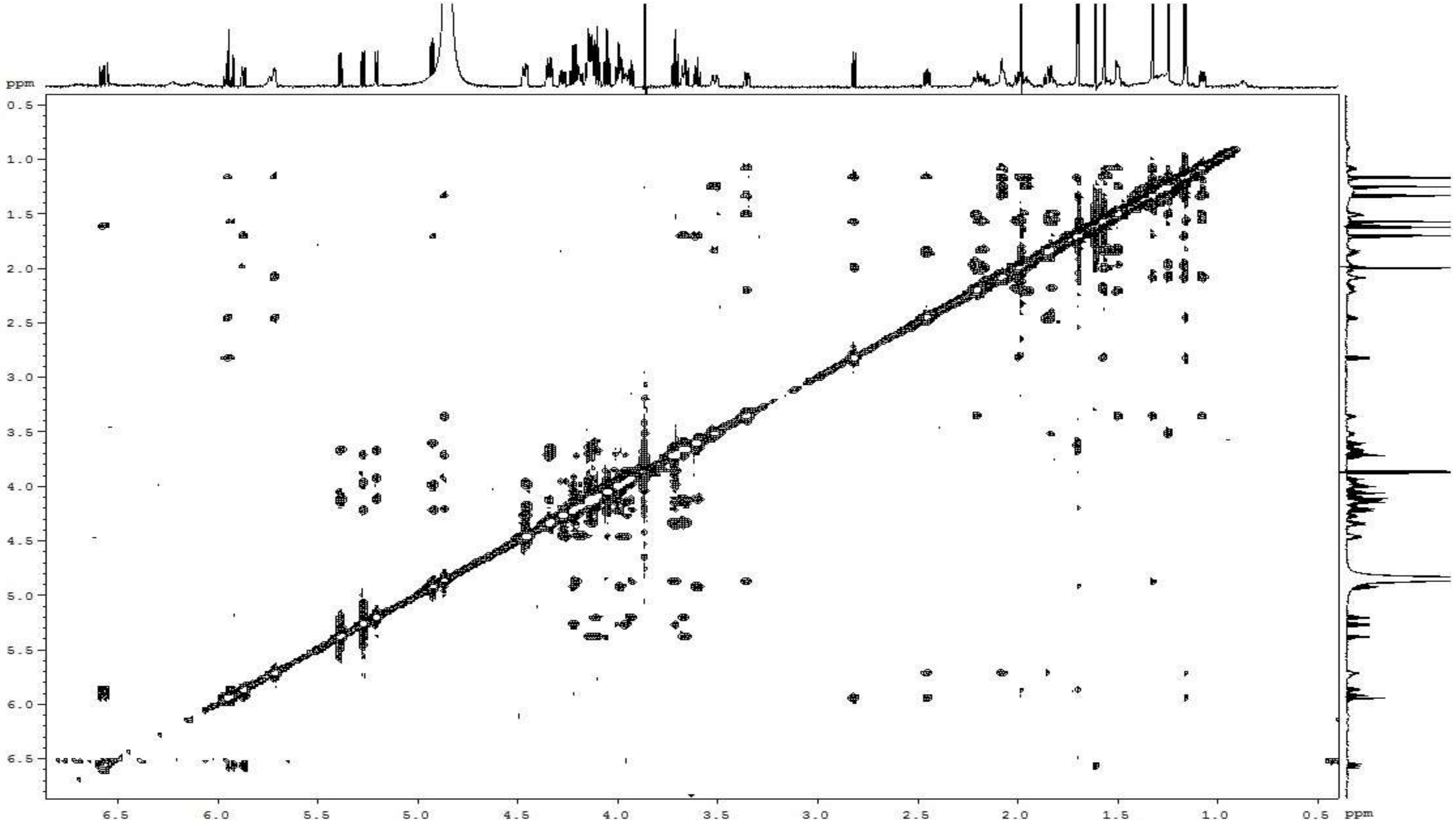

Figure S15. UV spectrum of pacificusoside D (**1**) in MeOH.

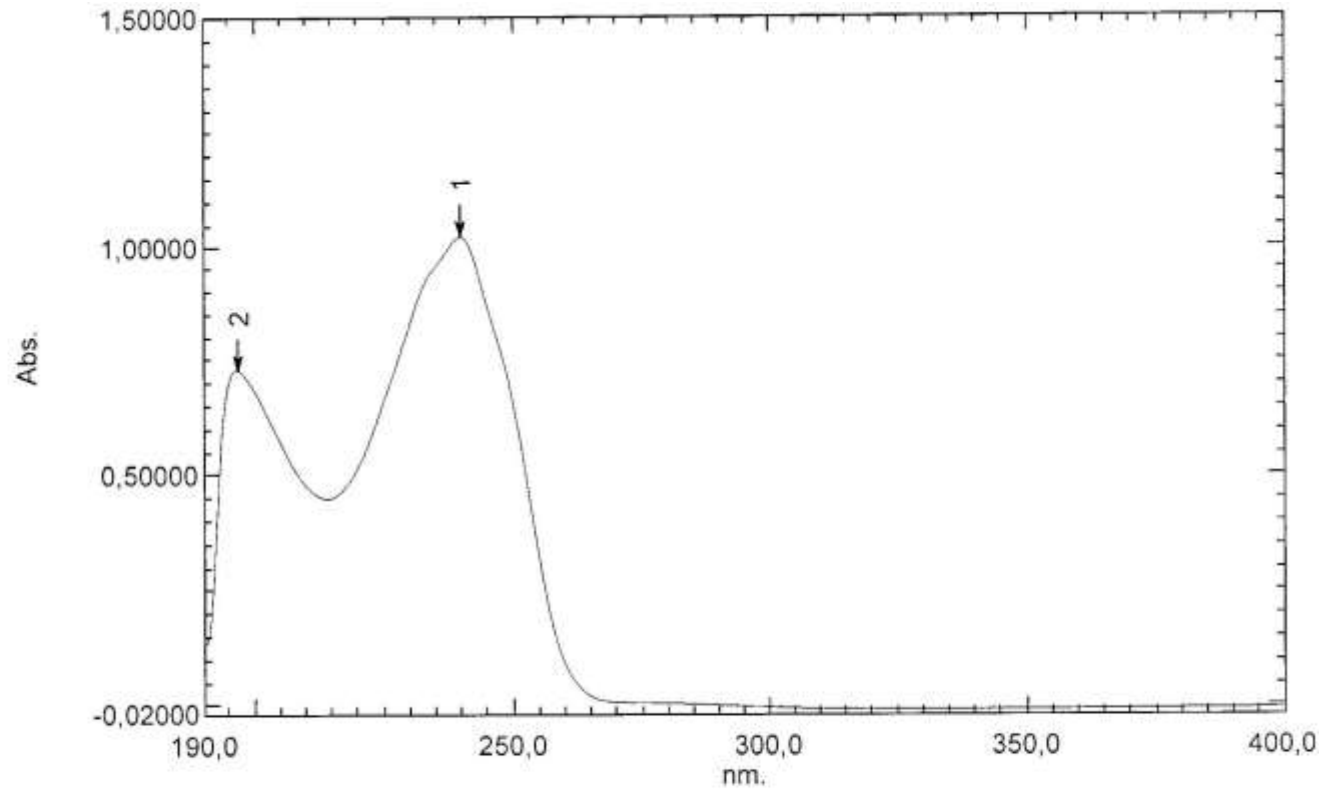

Figure S16. HRESIMS spectrum of pacificusoside E (**2**).

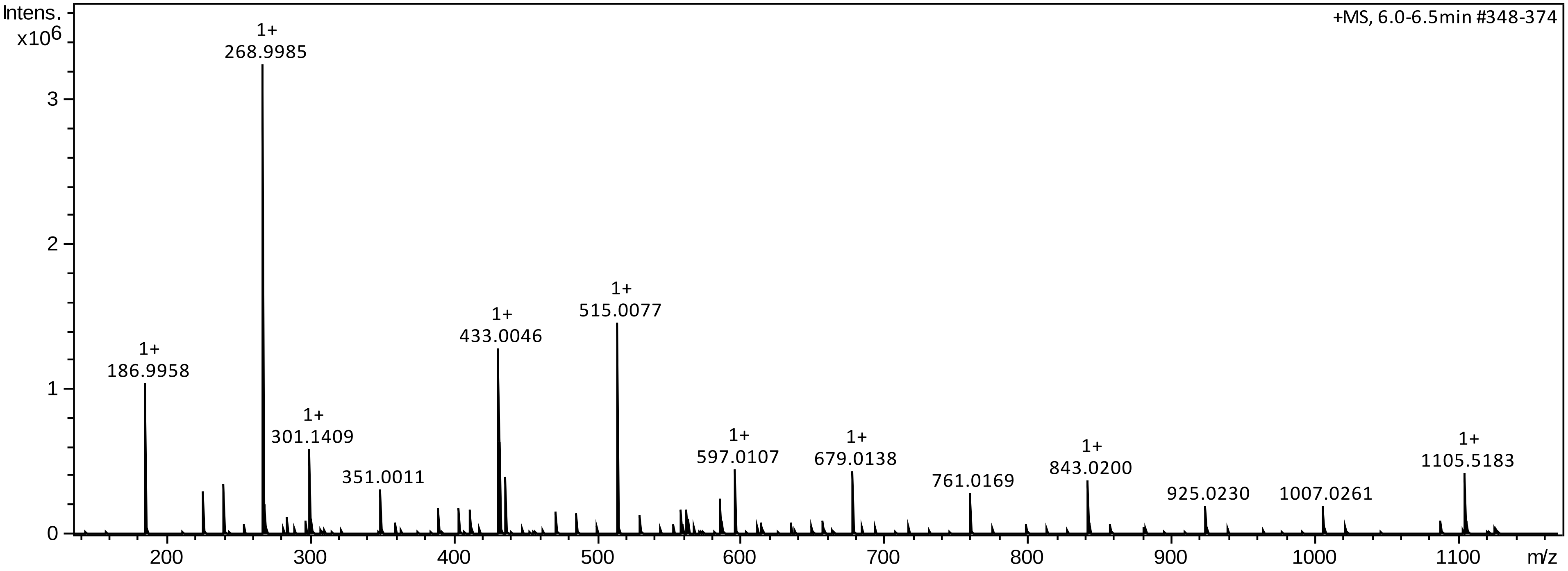

Figure S17. IR spectrum of pacificusoside E (**2**) in KBr.

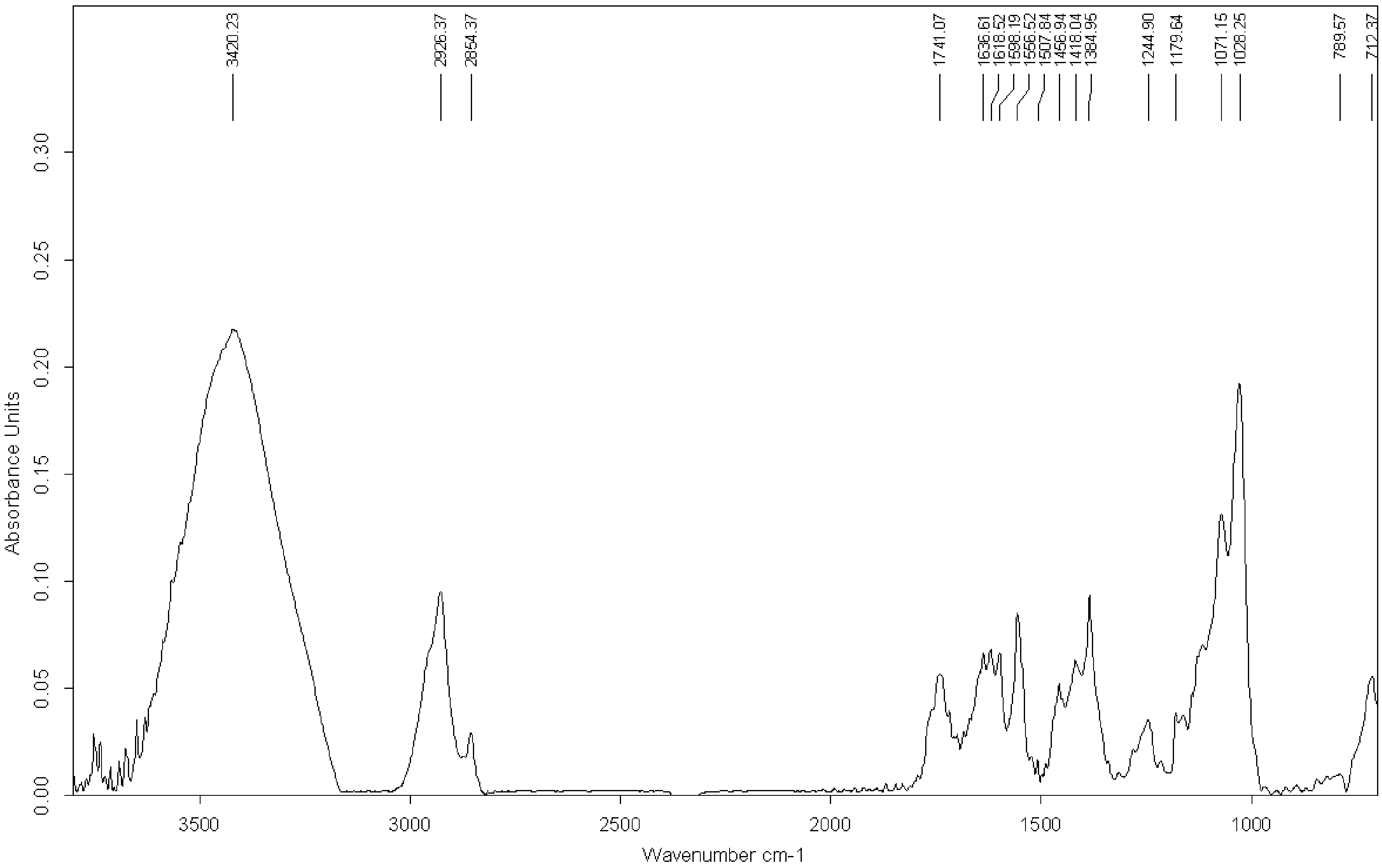

Figure S18. ^{1}H-NMR spectrum of pacificusoside E (**2**) in C_5D_5N.

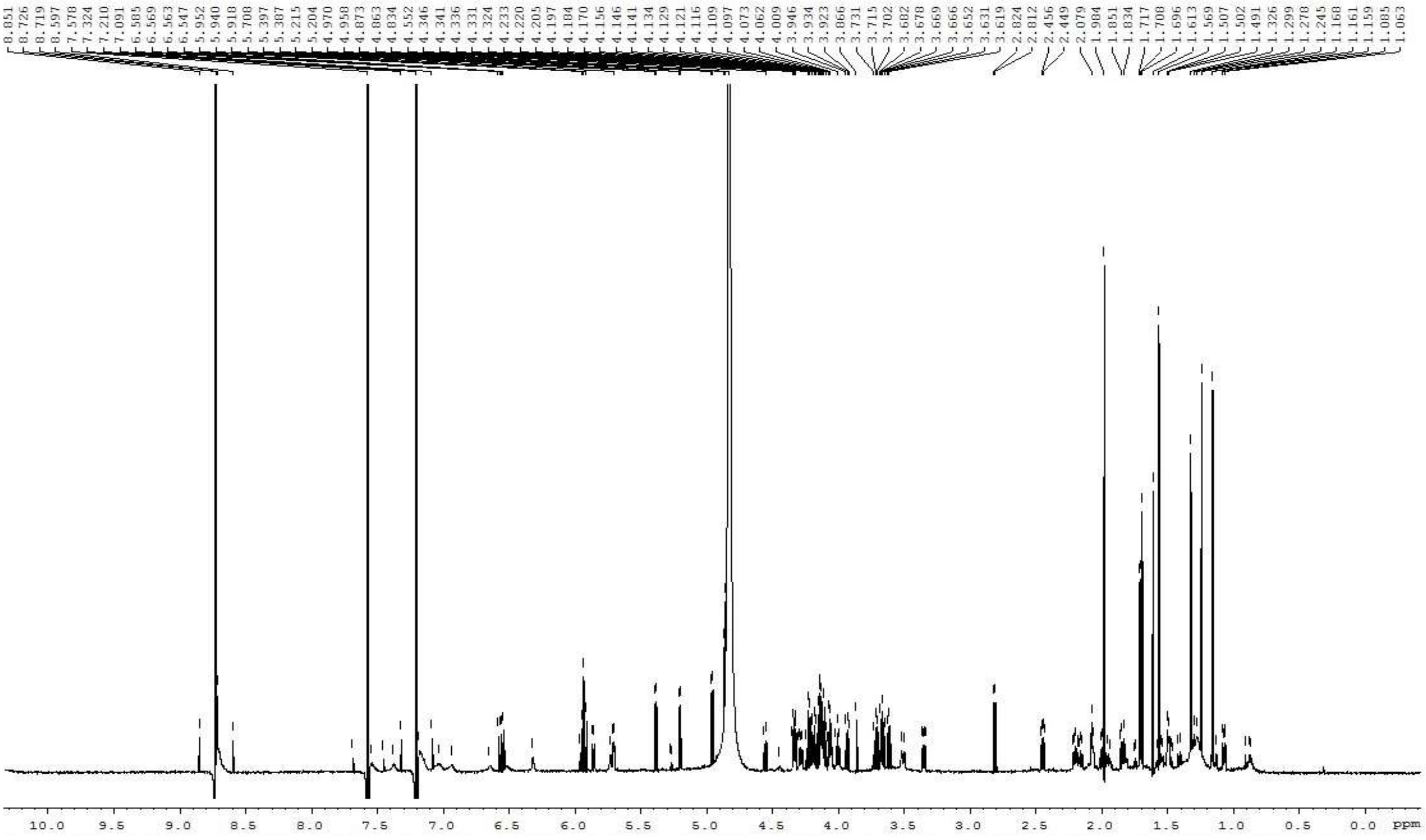

Figure S19. ^{13}C-NMR spectrum of pacificusoside E (**2**) in C_5D_5N.

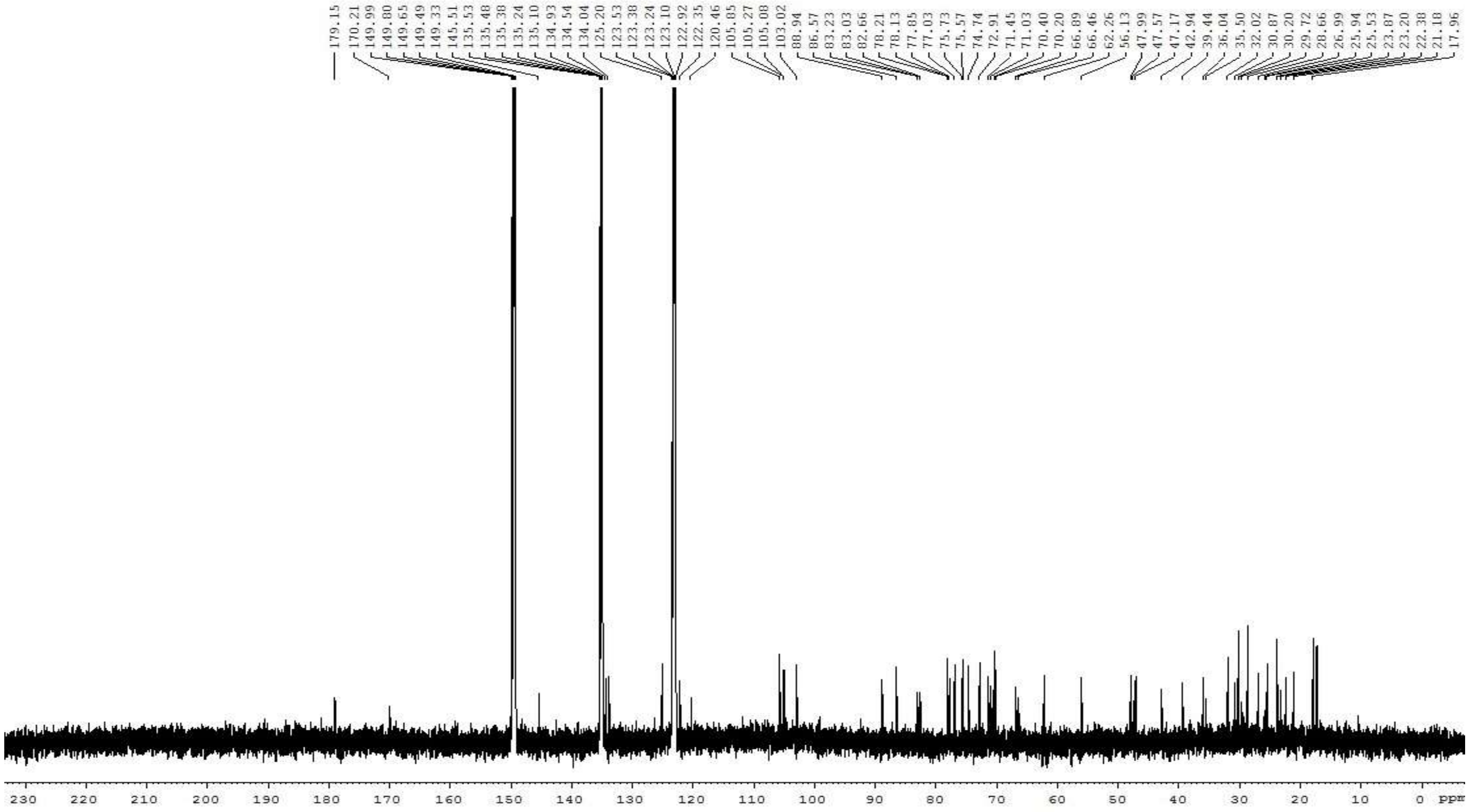

Figure S20. ^1H-^1H COSY spectrum of pacificusoside E (**2**) in C_5D_5N.

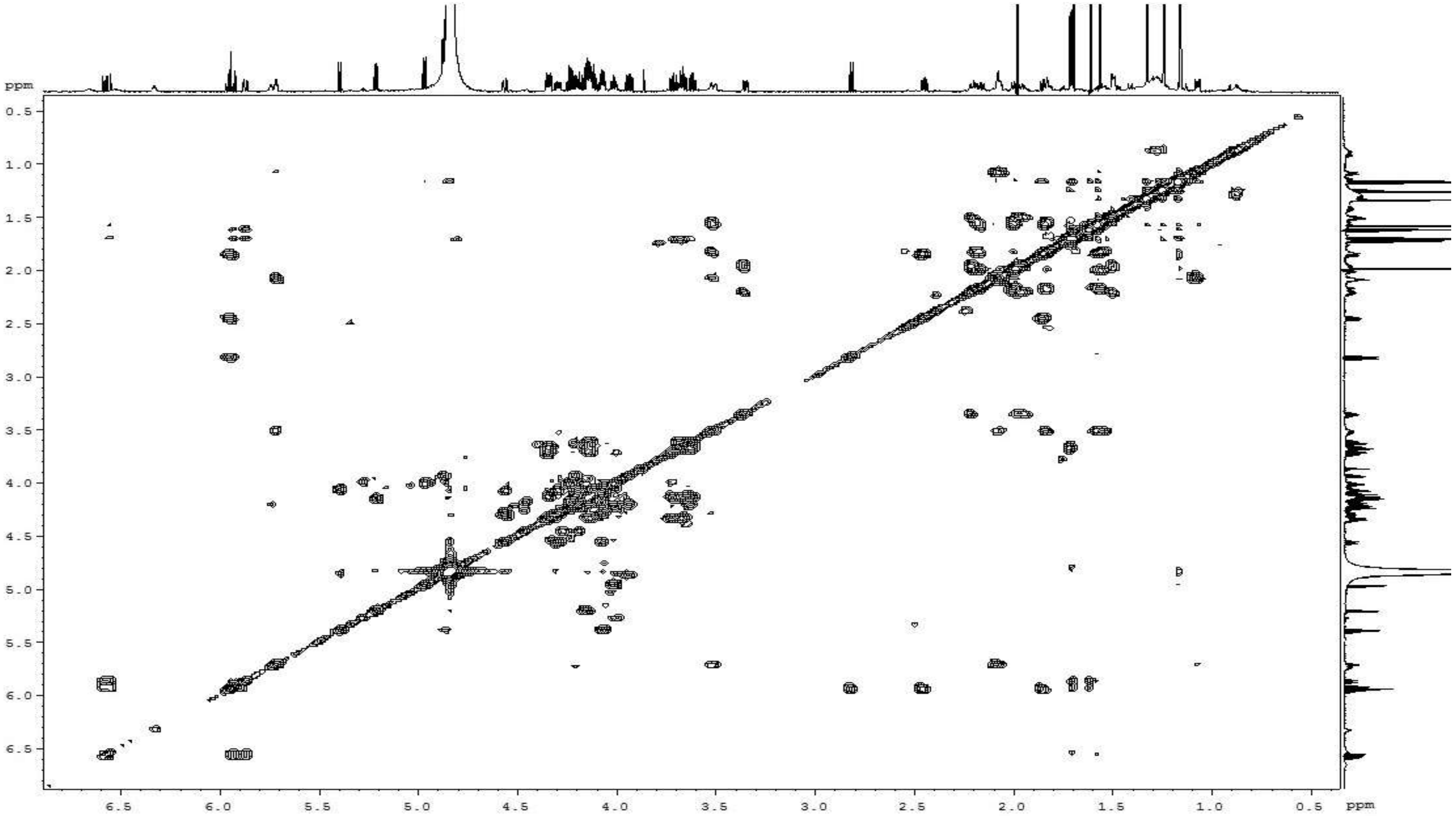

Figure S21. HSQC spectrum of pacificusoside E (**2**) in C_5D_5N.

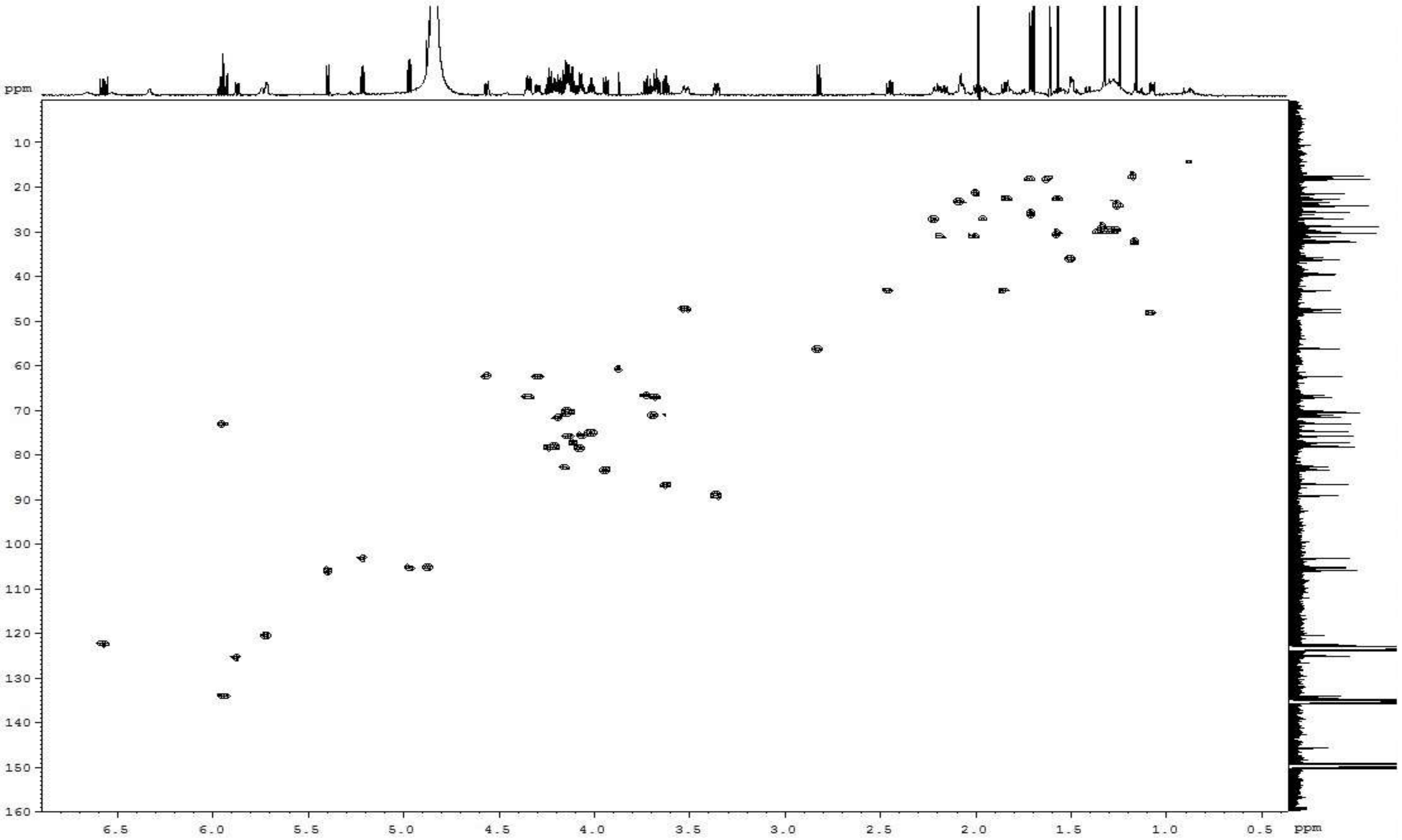

Figure S22. HMBC spectrum of pacificusoside E (**2**) in C_5D_5N.

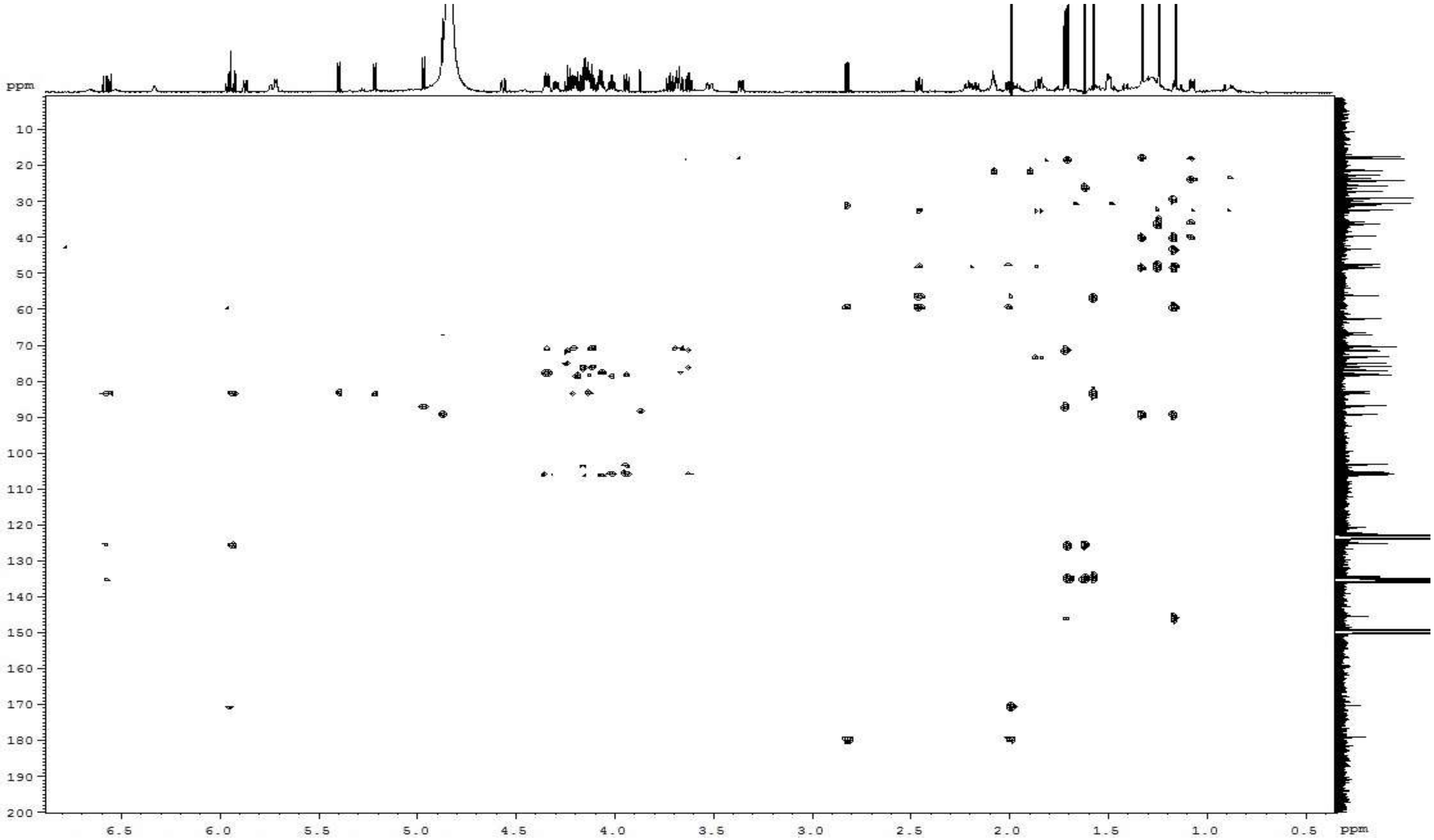

Figure S23. ROESY spectrum of pacificusoside E (**2**) in C_5D_5N.

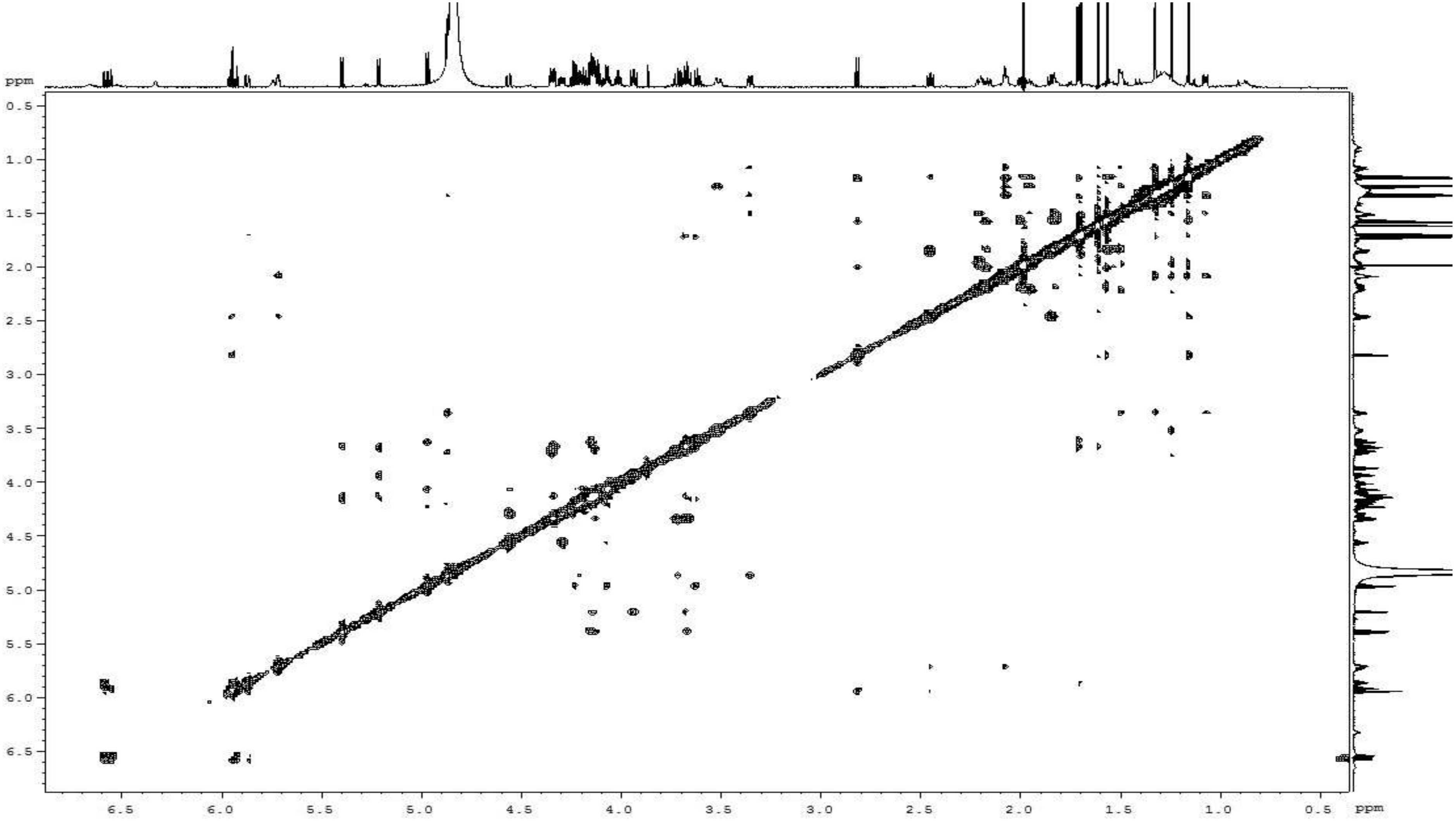

Figure S24. HRESIMS spectrum of pacificusoside F (**3**).

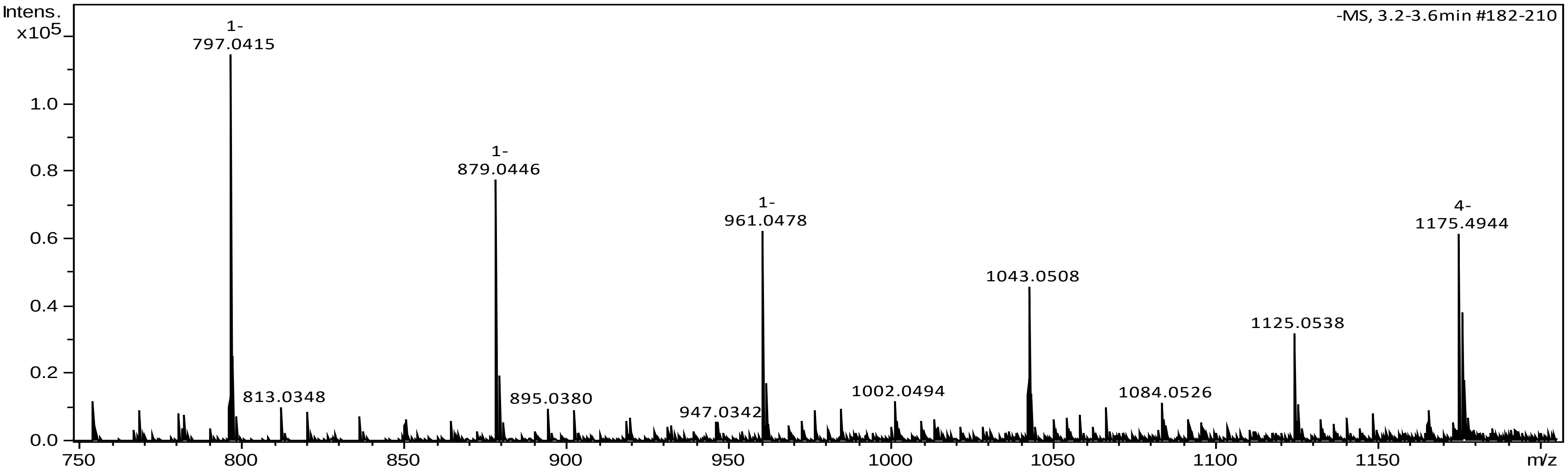

Figure S25. IR spectrum of pacificusoside F (**3**) in KBr.

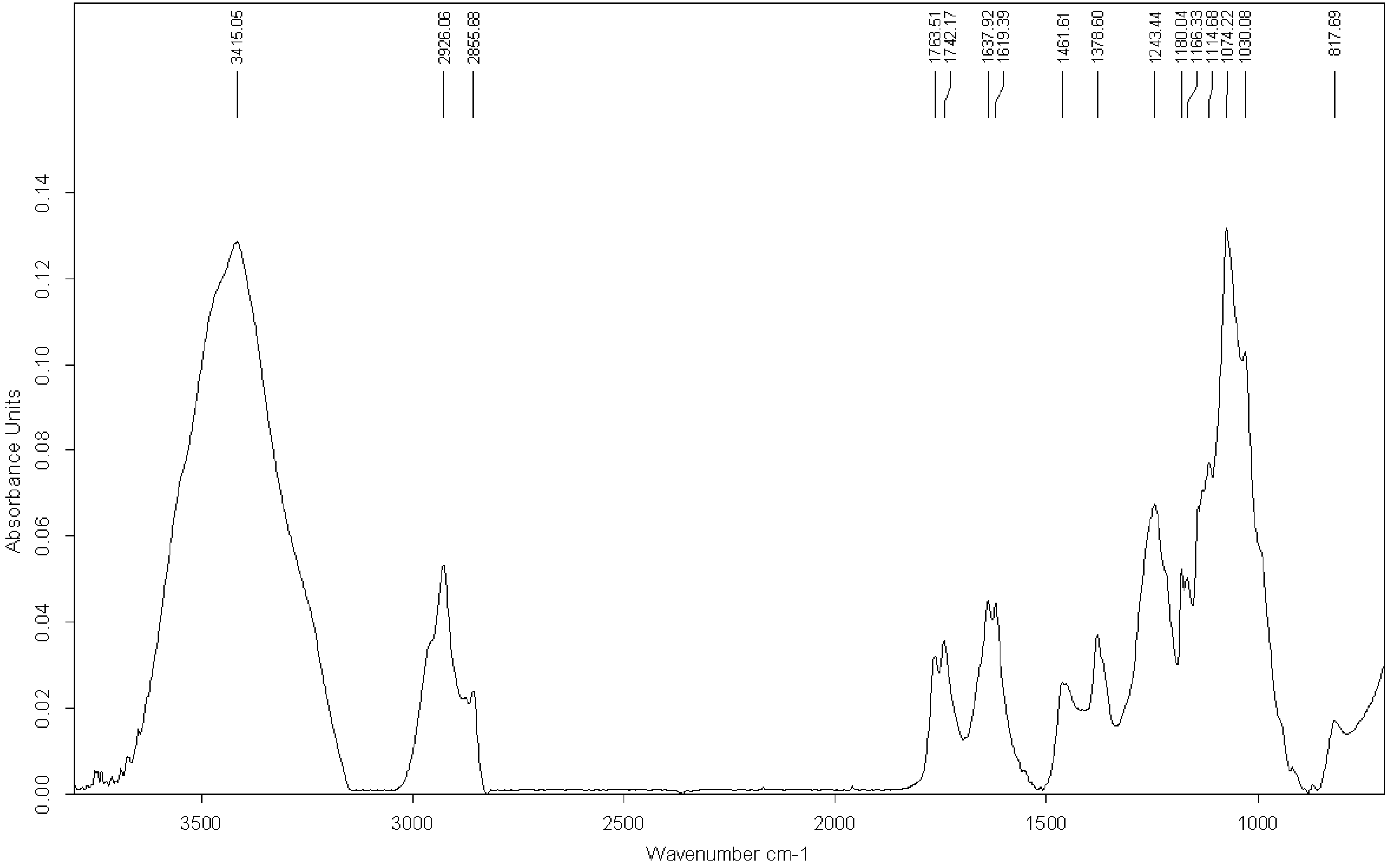

Figure S26. ^{1}H-NMR spectrum of pacificusoside F (**3**) in C_5D_5N.

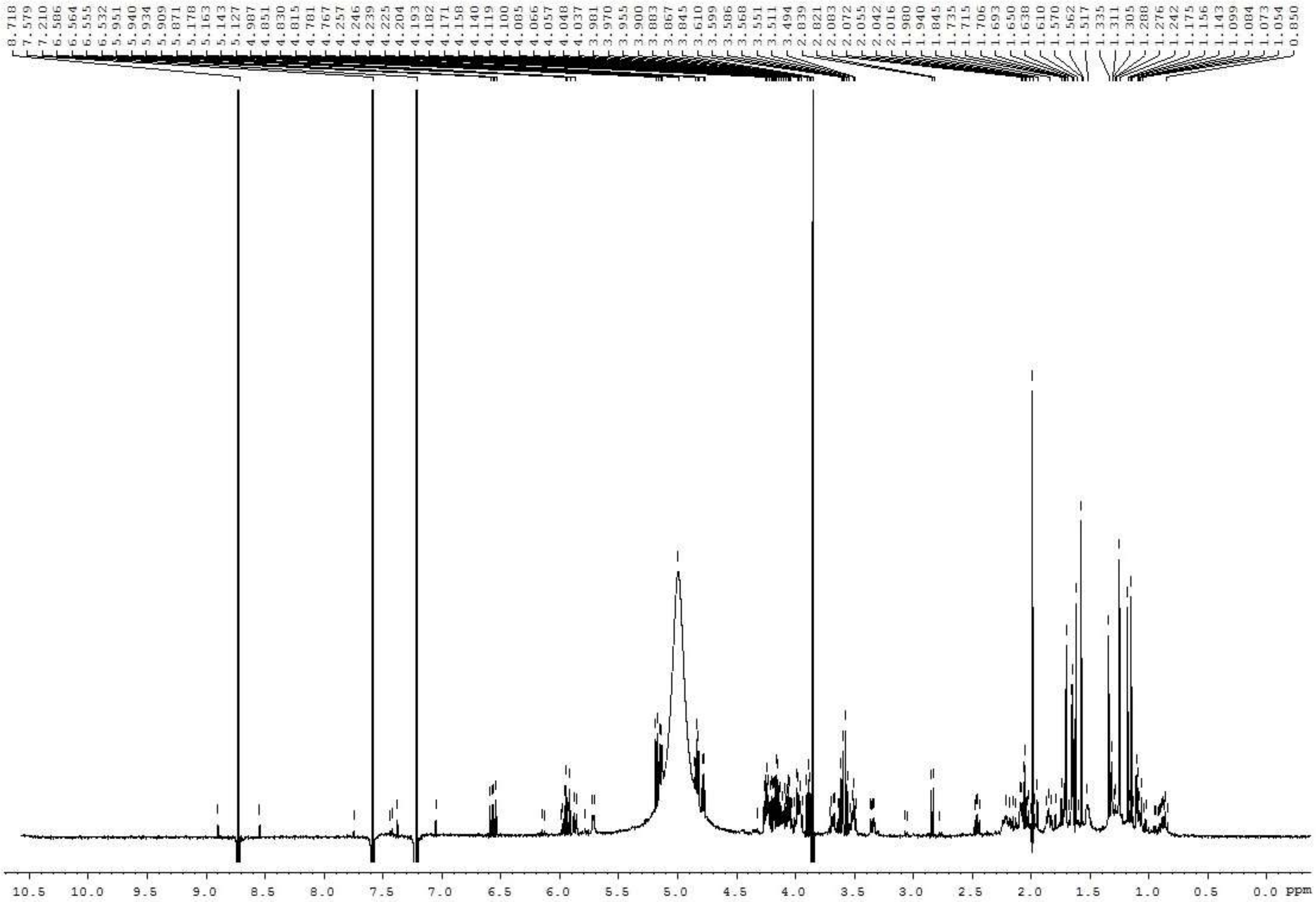

Figure S27. ^{13}C-NMR spectrum of pacificusoside F (**3**) in C_5D_5N.

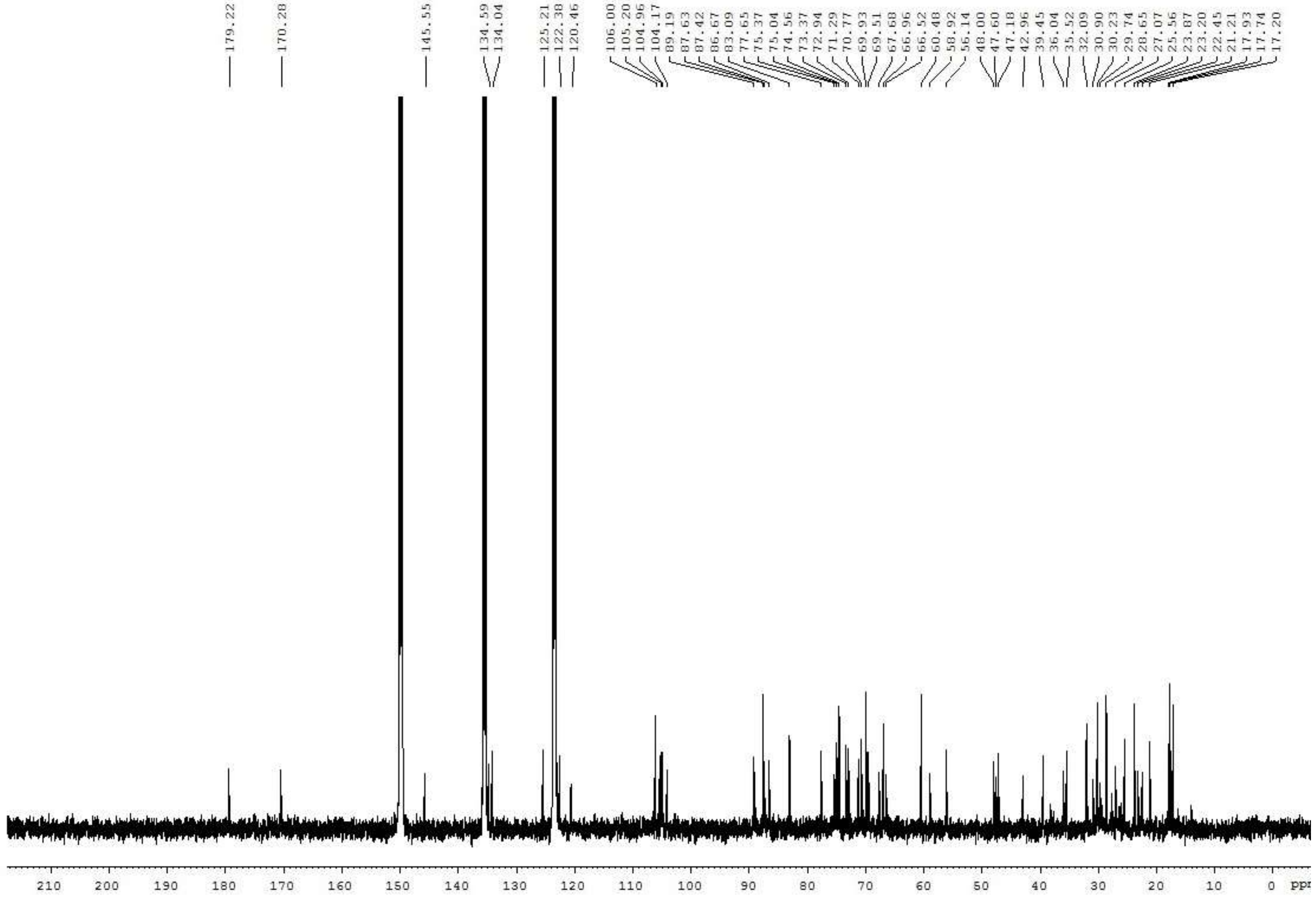

Figure S28. ^{1}H-^{1}H COSY spectrum of pacificusoside F (**3**) in C_5D_5N.

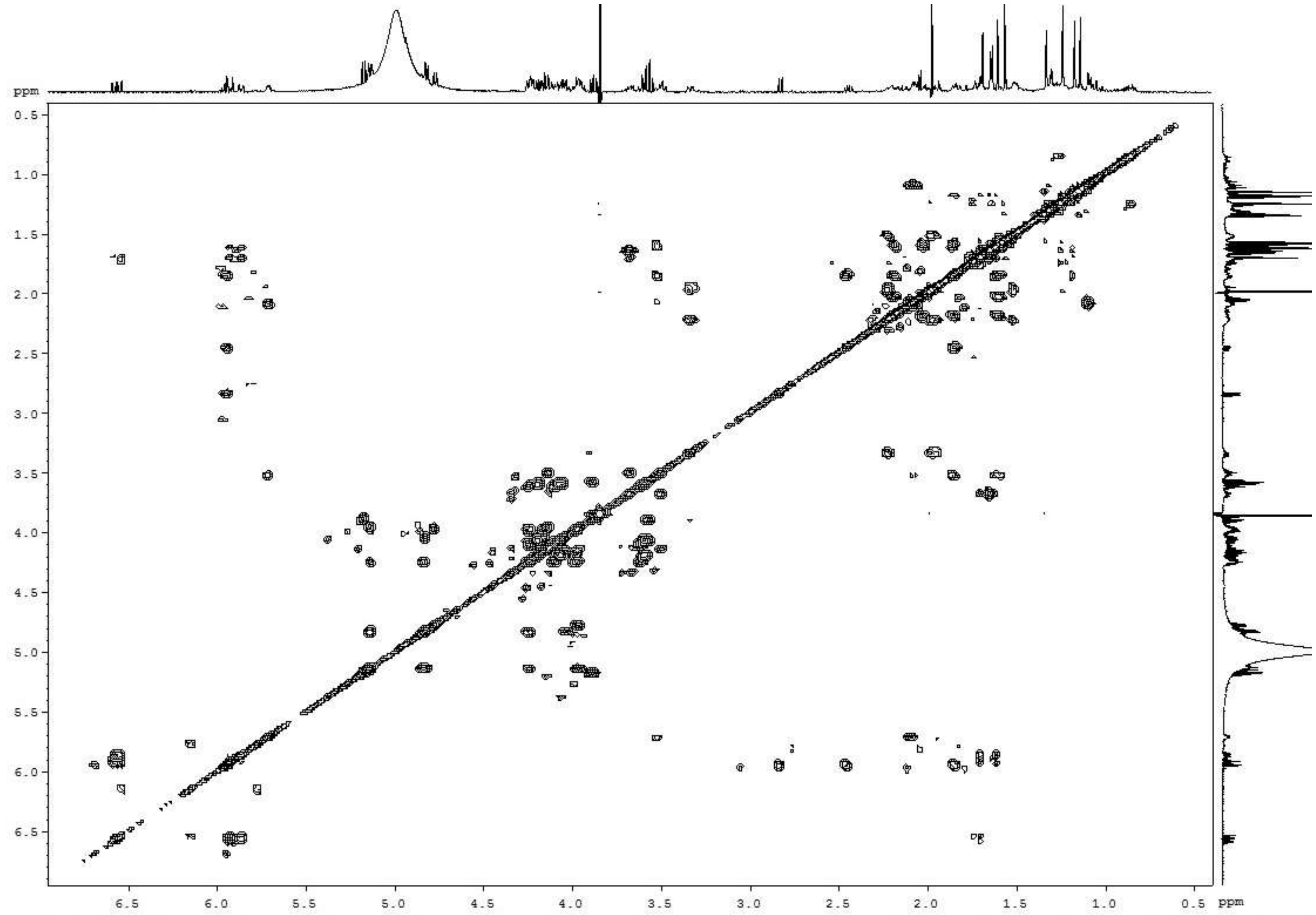

Figure S29. HSQC spectrum of pacificusoside F (**3**) in C_5D_5N.

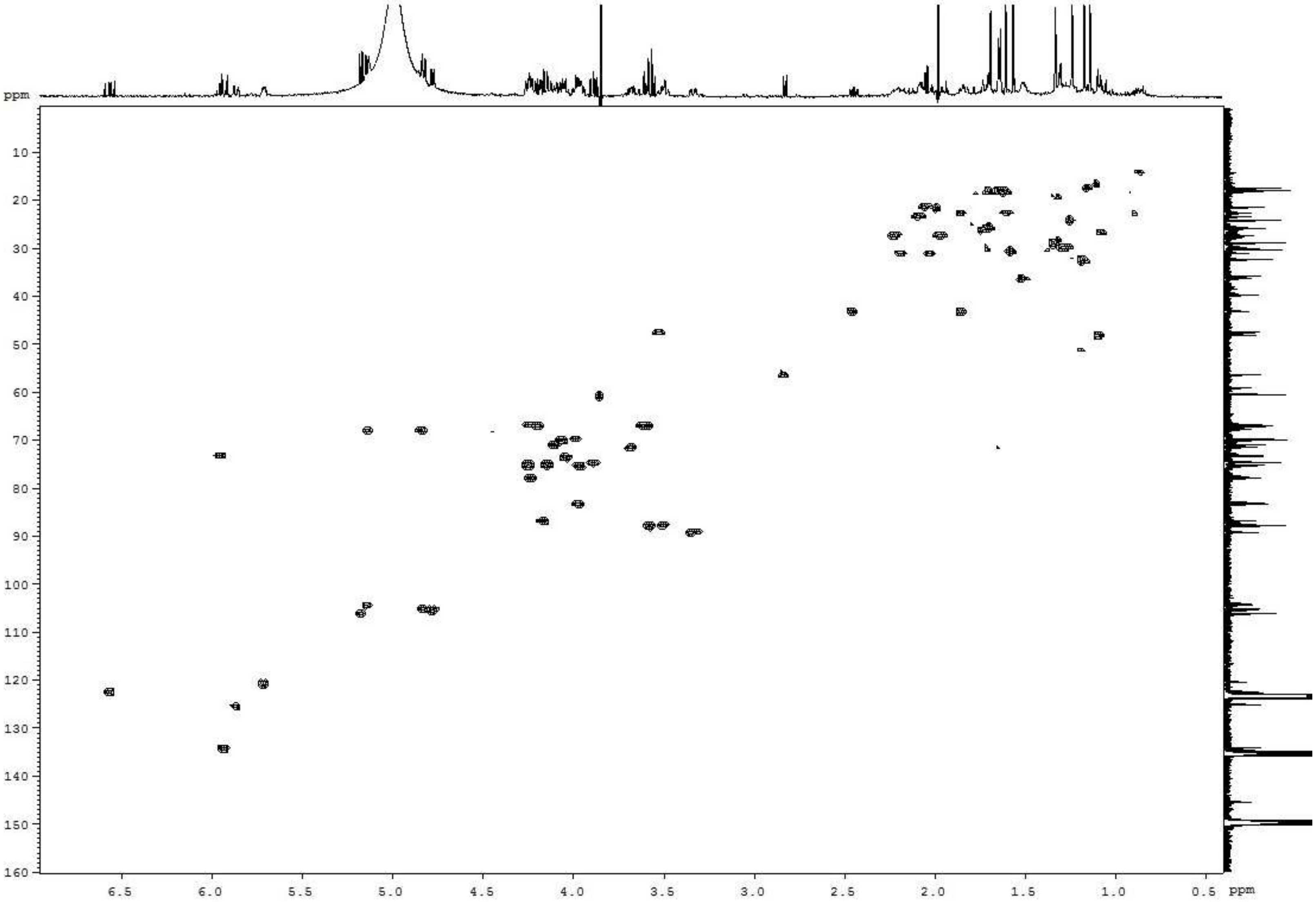

Figure S30. HMBC spectrum of pacificusoside F (**3**) in C_5D_5N.

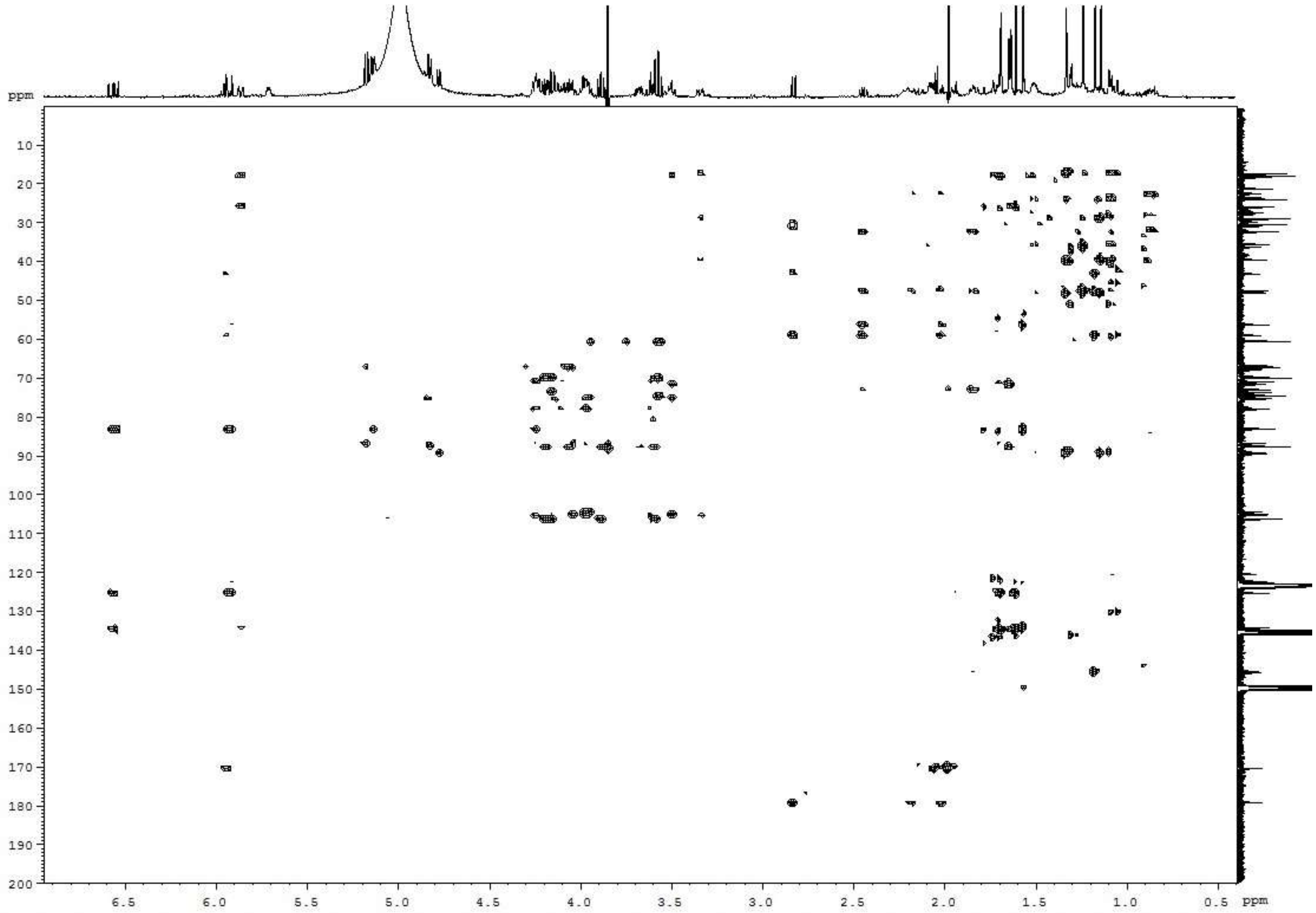

Figure S31. ROESY spectrum of pacificusoside F (**3**) in C_5D_5N.

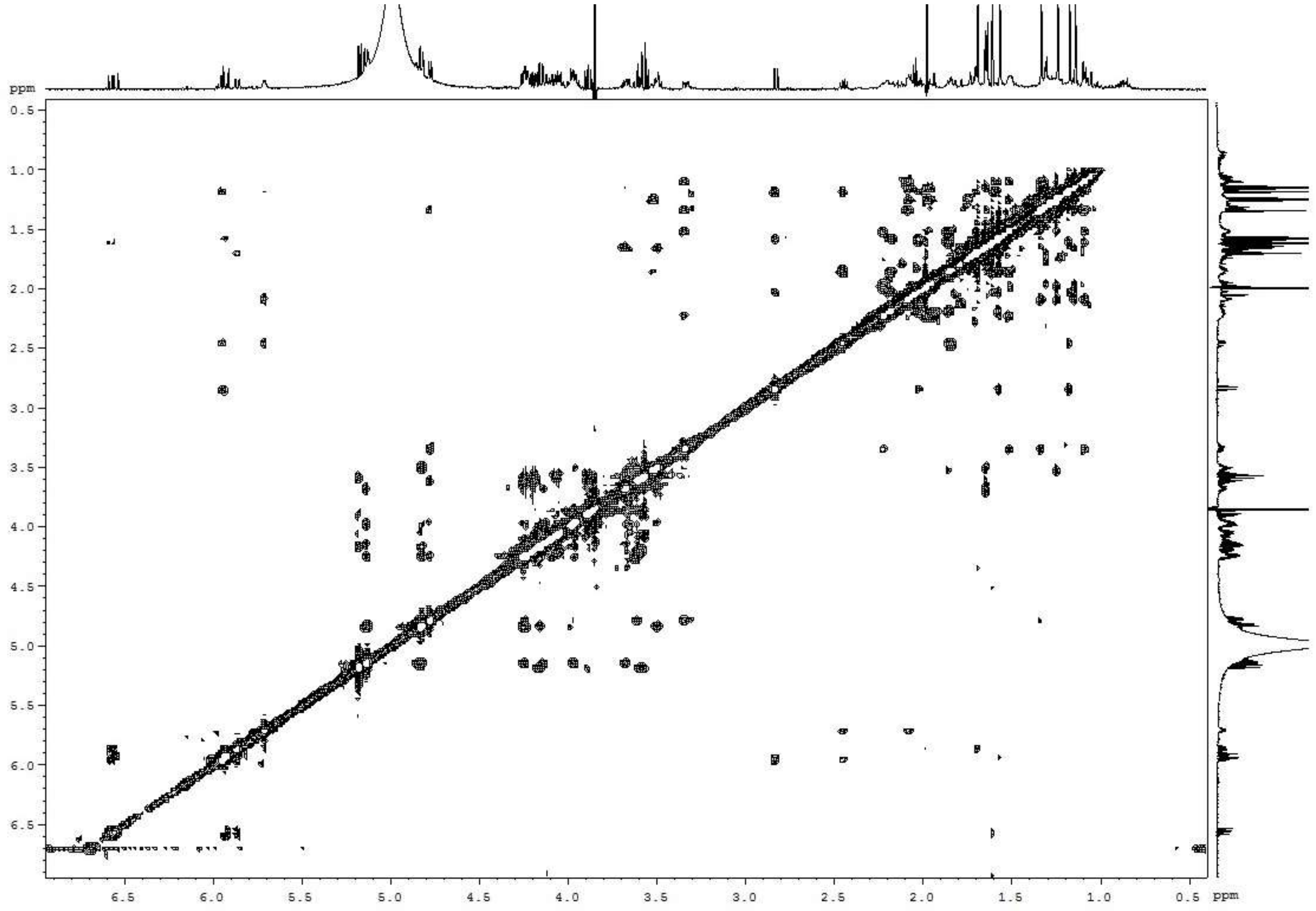

Figure S32. HRESIMS spectrum of pacificusoside G (**5**).

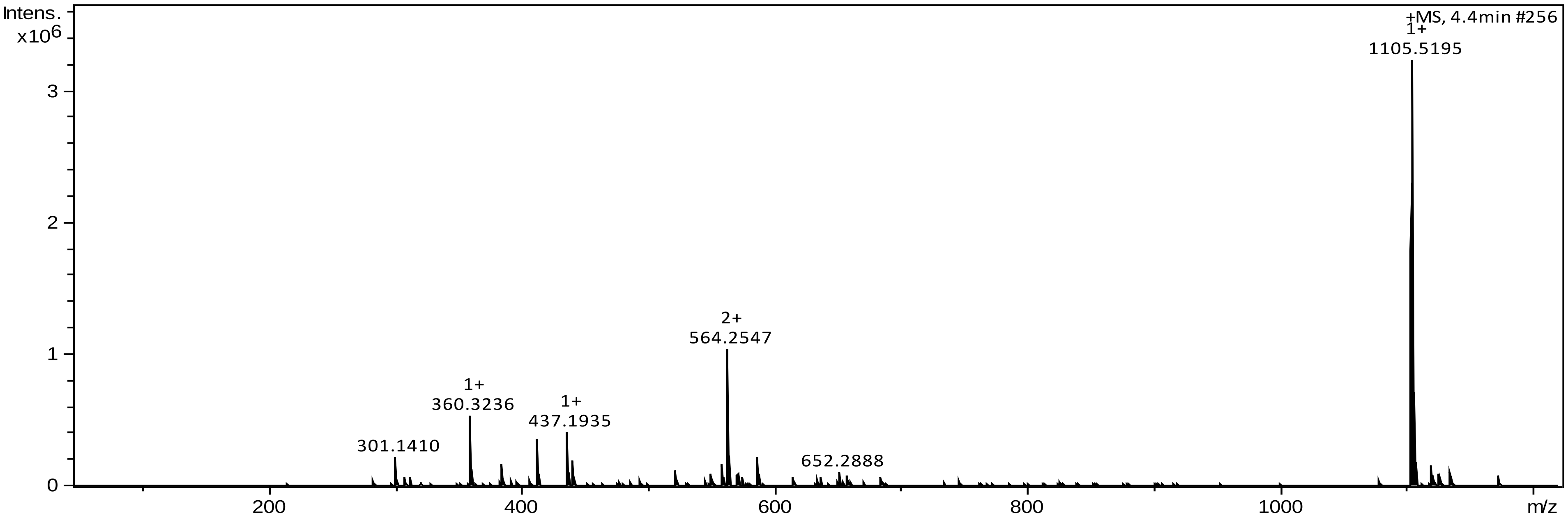

Figure S33. IR spectrum of pacificusoside G (**5**) in KBr.

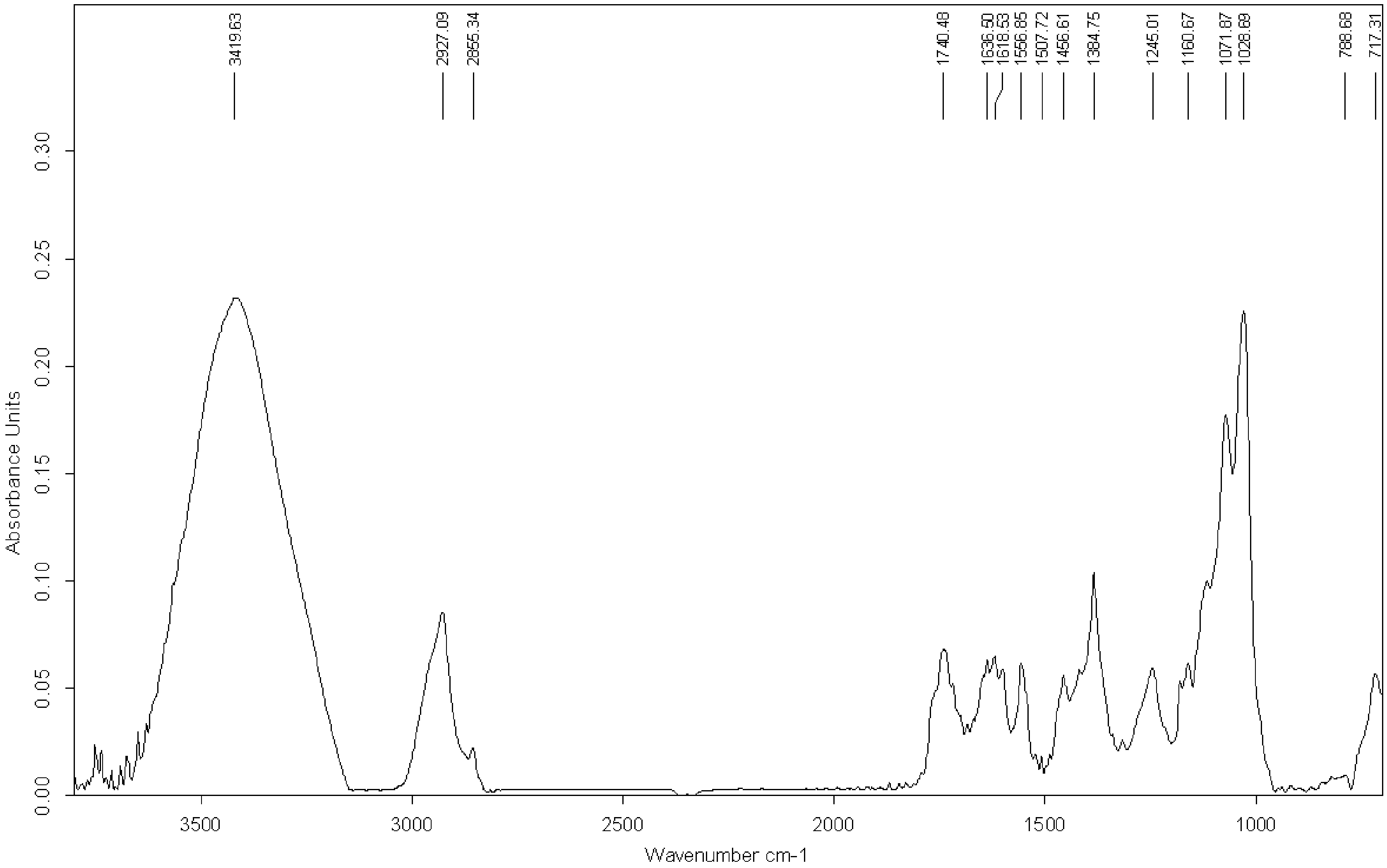

Figure S34. ^{1}H-NMR spectrum of pacificusoside G (**5**) in C_5D_5N.

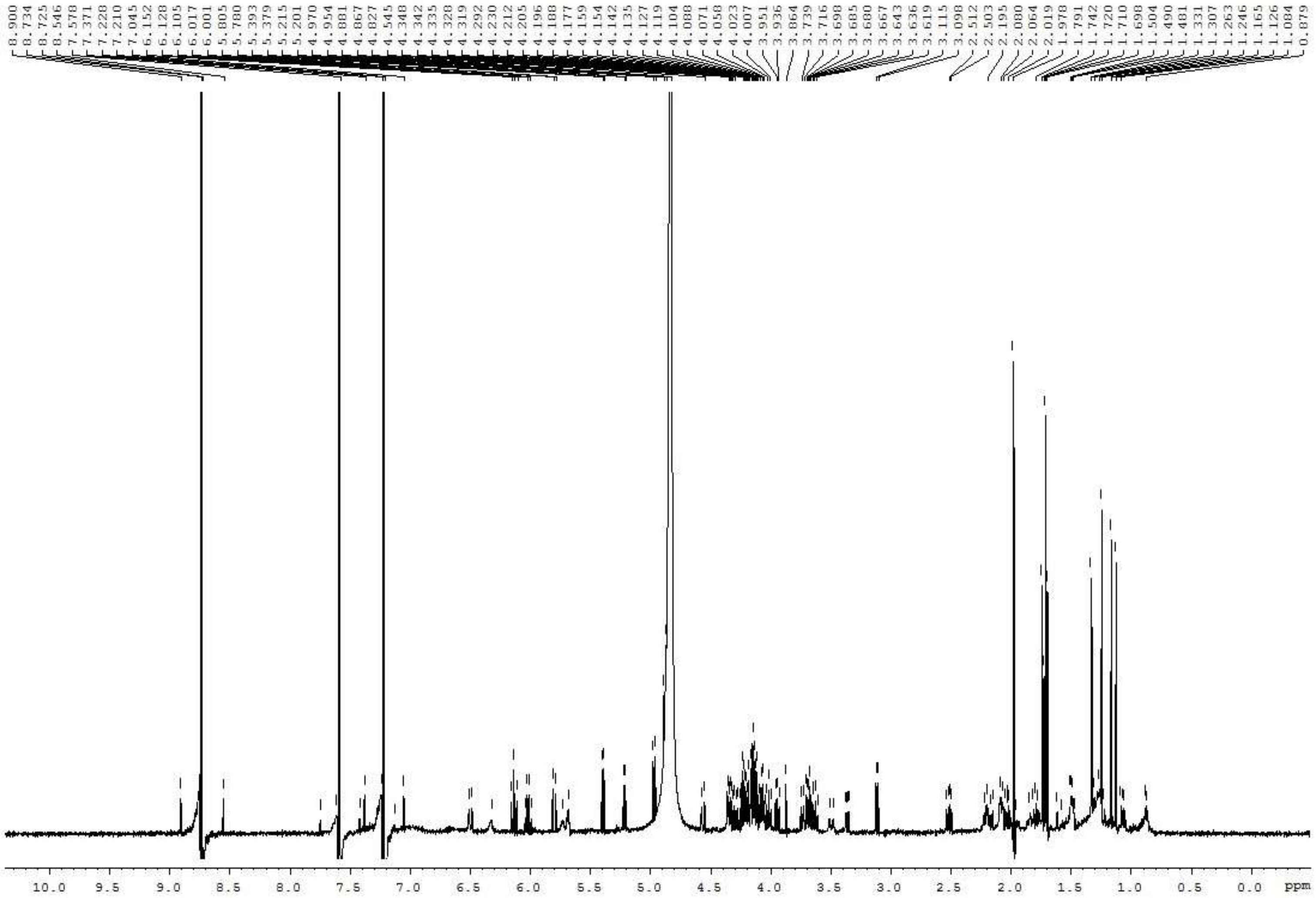

Figure S35. Expansion №1 of ^{1}H-NMR spectrum of pacificusoside G (**5**) in C_5D_5N.

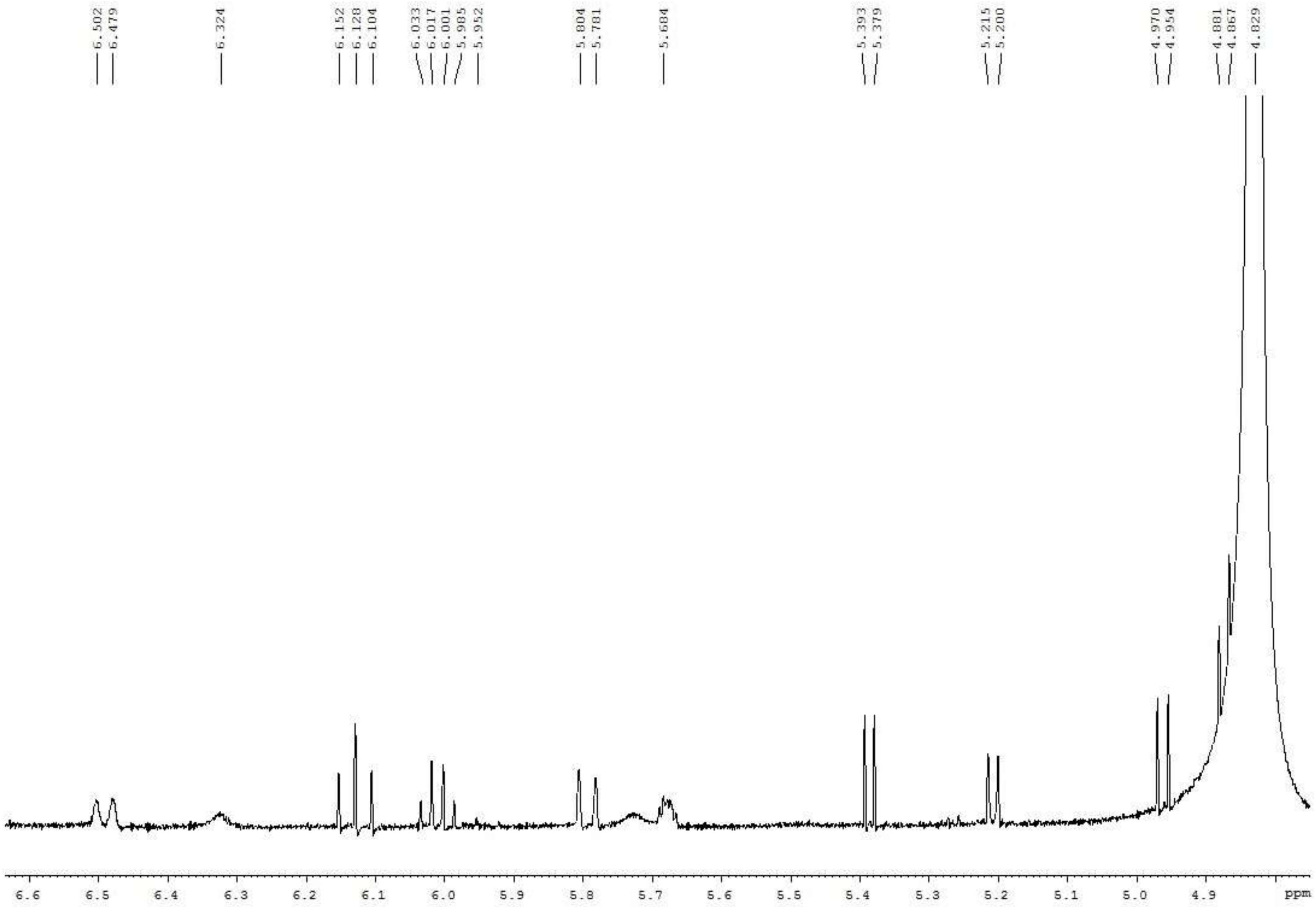

Figure S36. Expansion №2 of ^{1}H-NMR spectrum of pacificusoside G (**5**) in C_5D_5N.

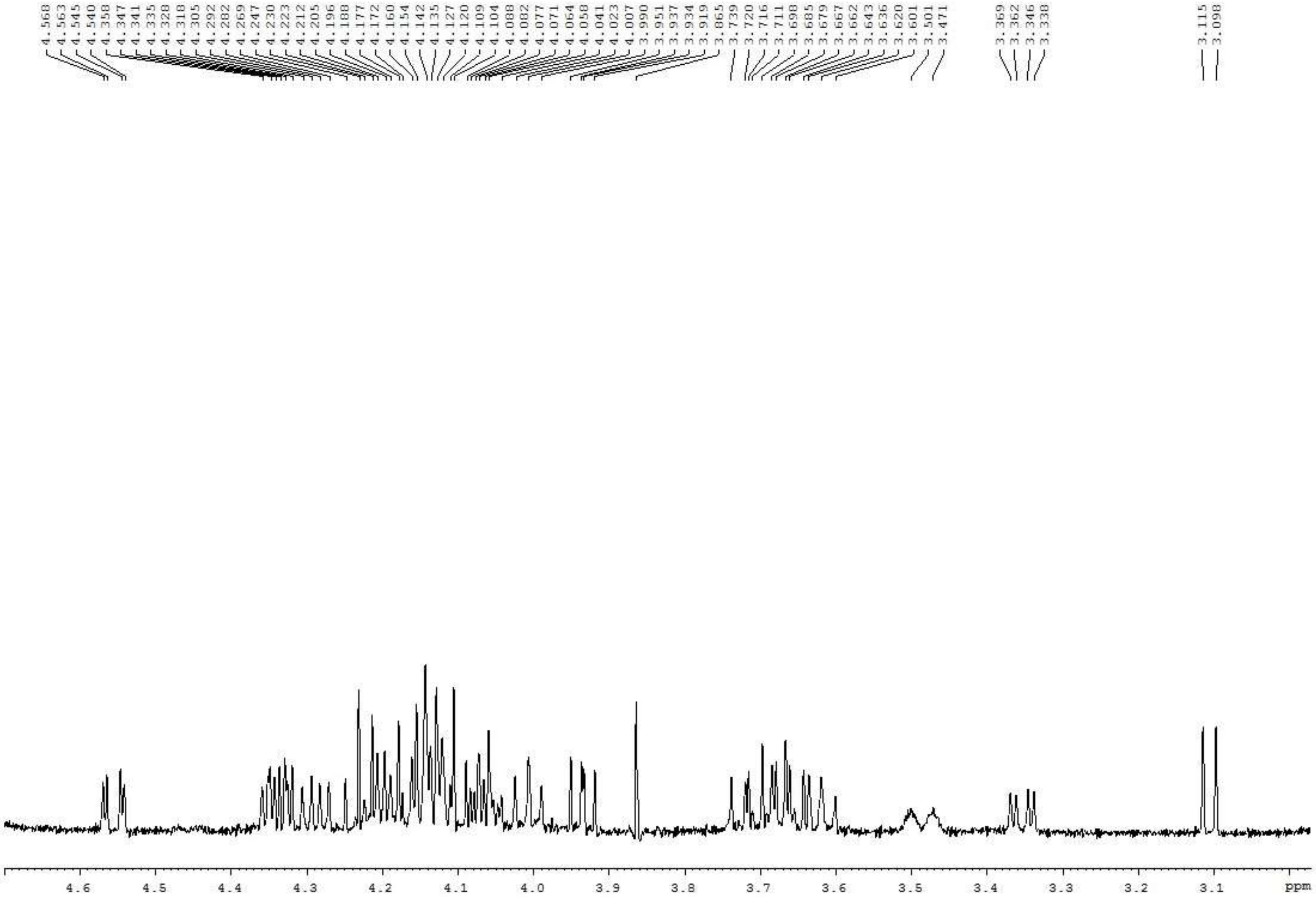

Figure S37. Expansion №3 of ^{1}H-NMR spectrum of pacificusoside G (**5**) in C_5D_5N.

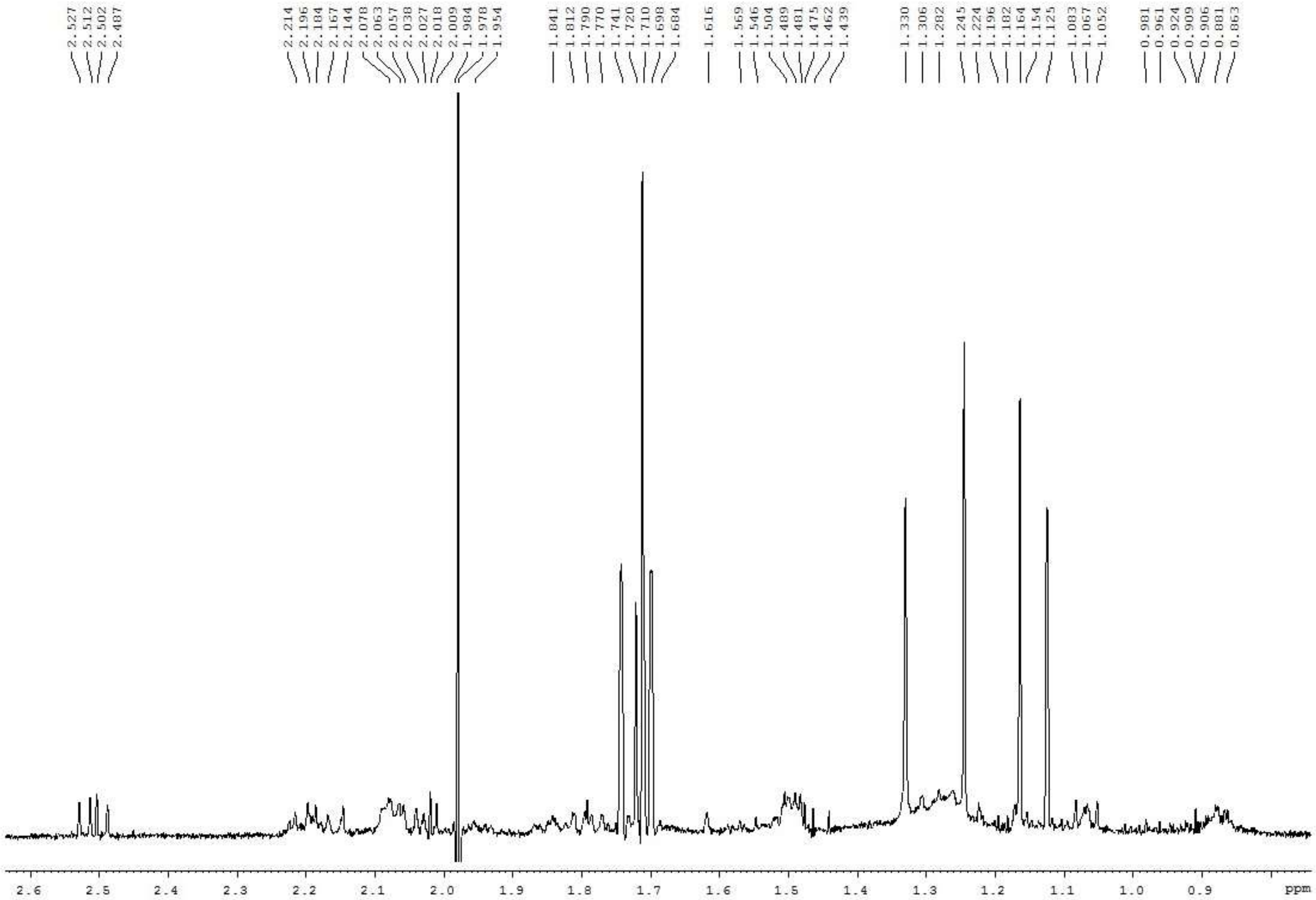

Figure S38. ^{13}C-NMR spectrum of pacificusoside G (**5**) in C_5D_5N.

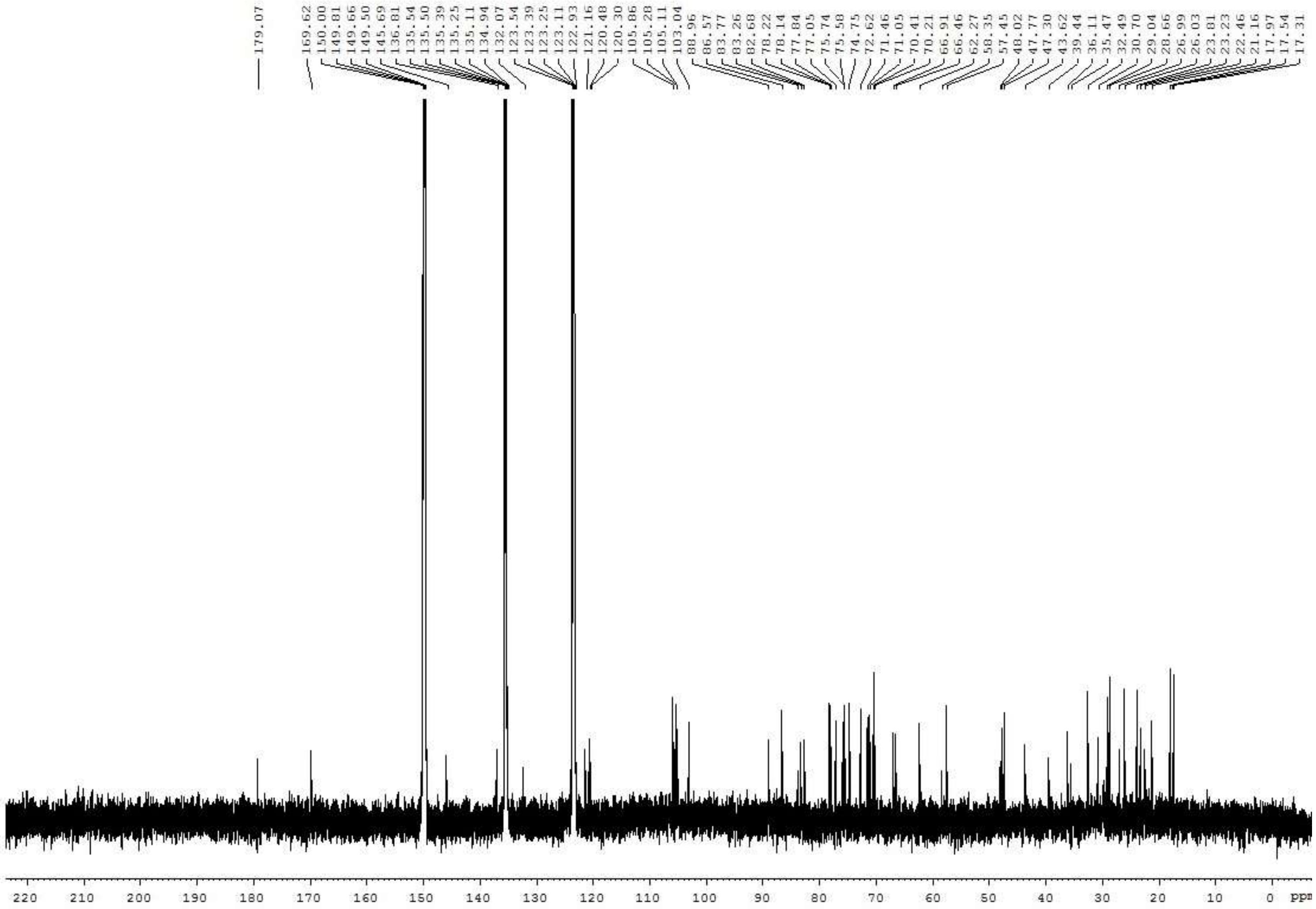

Figure S39. Expansion №1 of ^{13}C-NMR spectrum of pacificusoside G (**5**) in C_5D_5N.

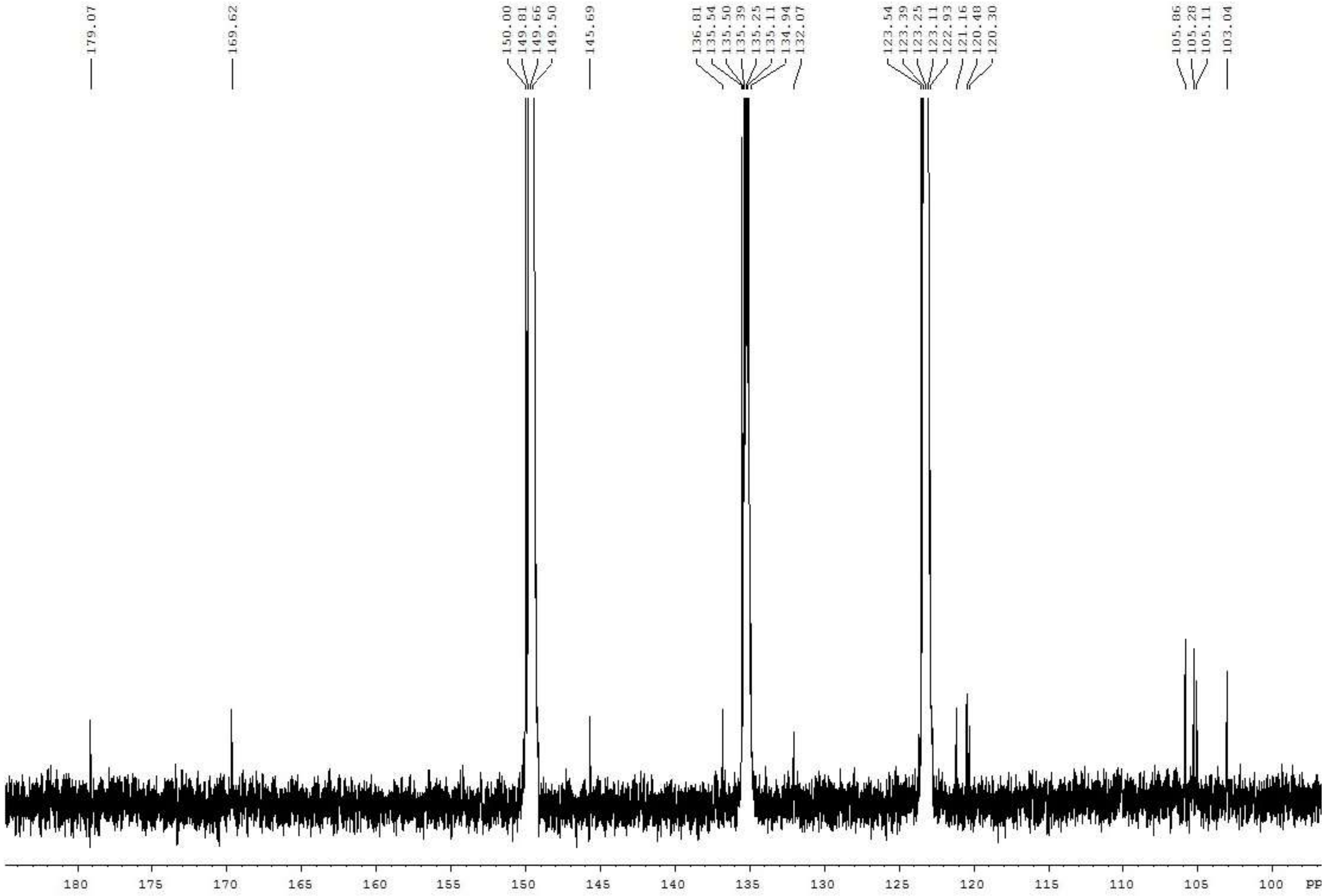

Figure S40. Expansion №2 of ^{13}C-NMR spectrum of pacificusoside G (**5**) in C_5D_5N.

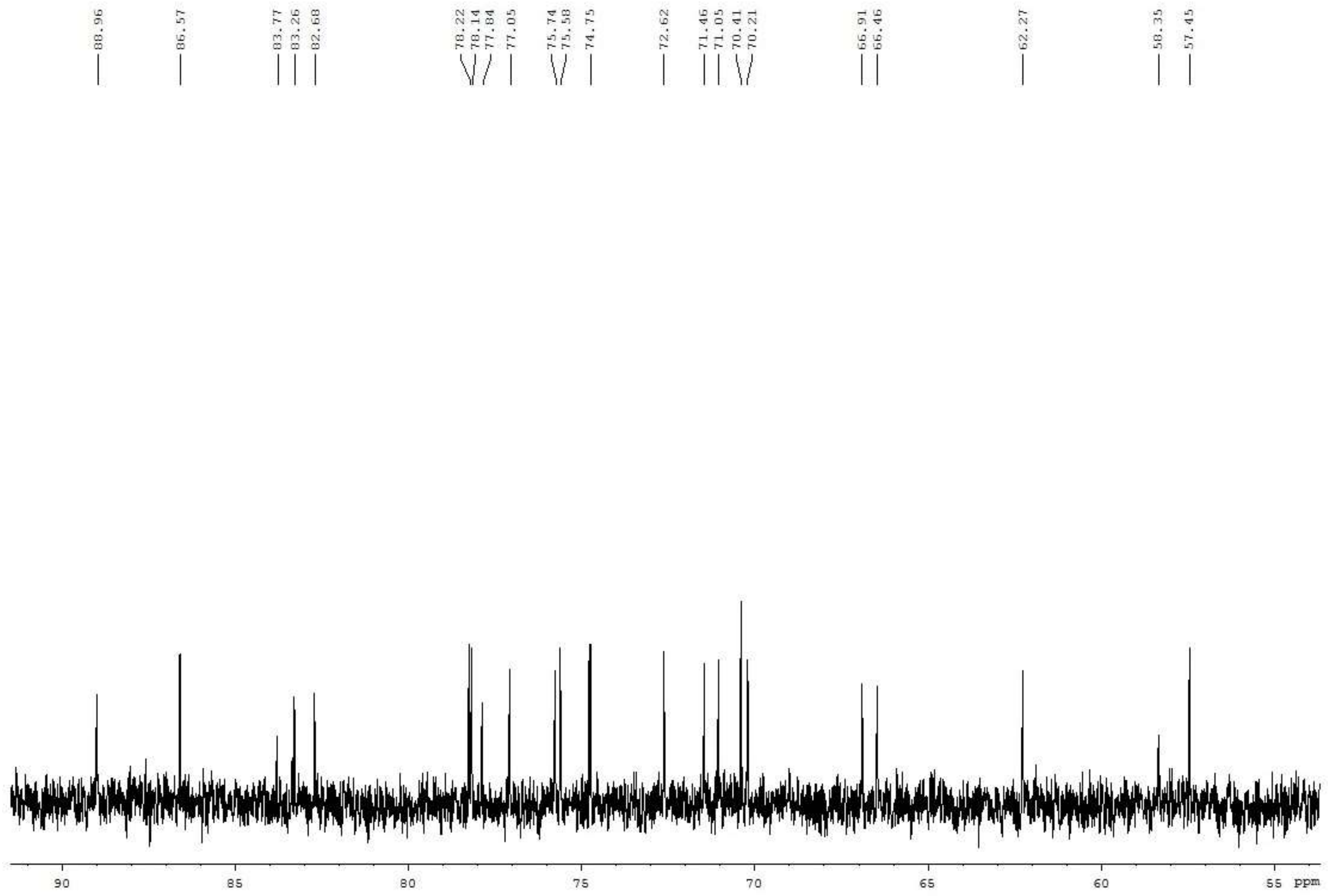

Figure S41. Expansion №3 of ^{13}C-NMR spectrum of pacificusoside G (**5**) in C_5D_5N.

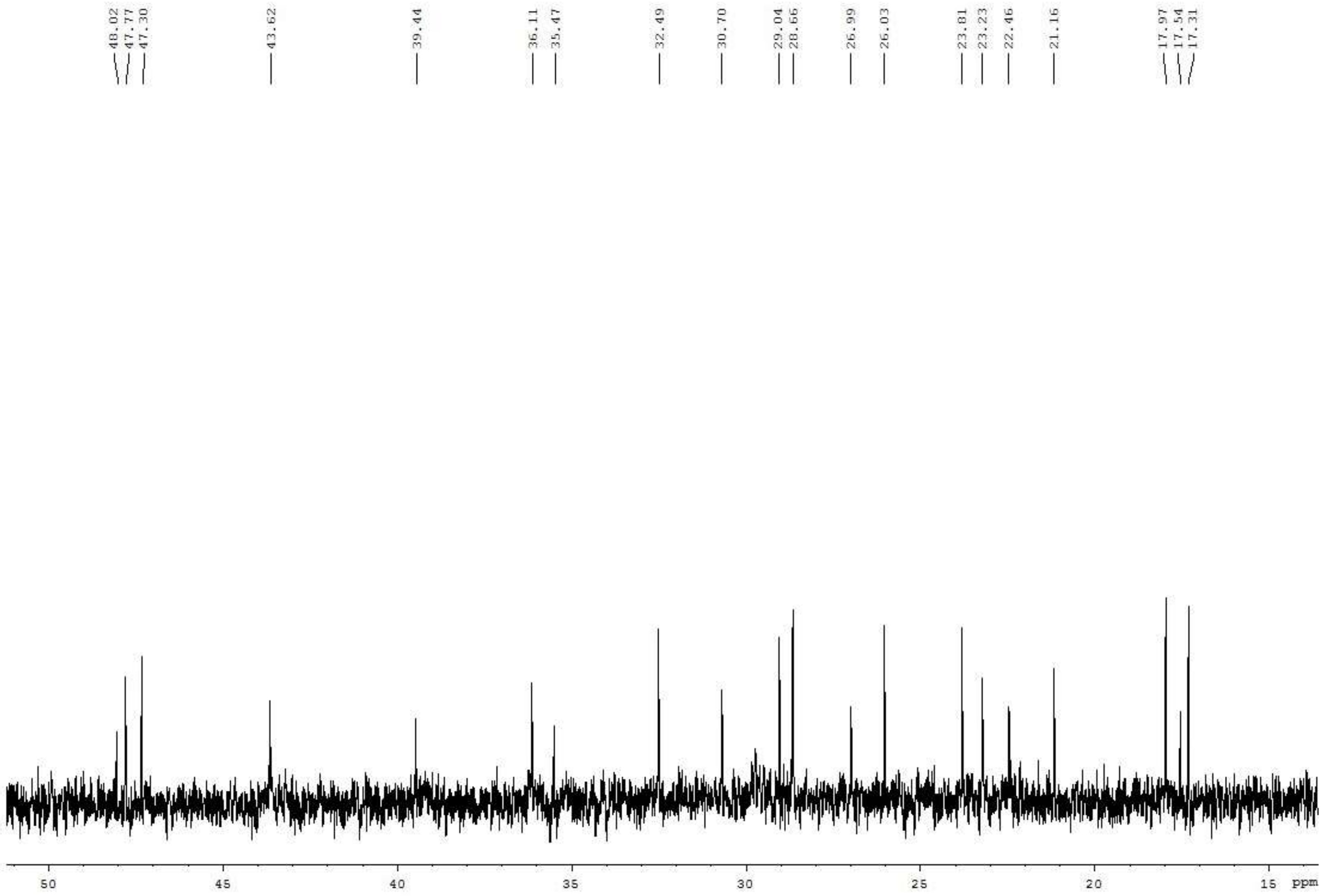

Figure S42. UV spectrum of pacificusoside G (**5**) in MeOH.

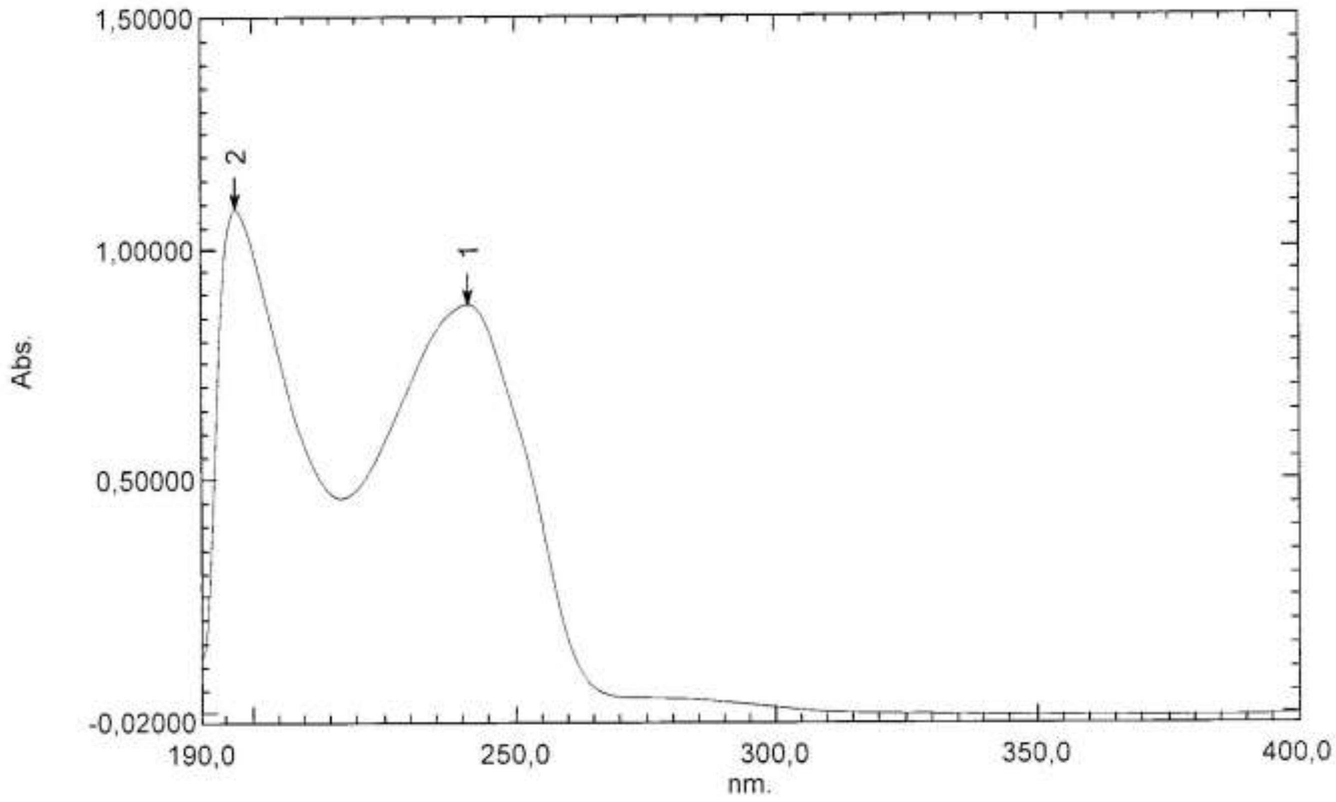

Figure S43. ^{1}H-^{1}H COSY spectrum of pacificusoside G (**5**) in C_5D_5N.

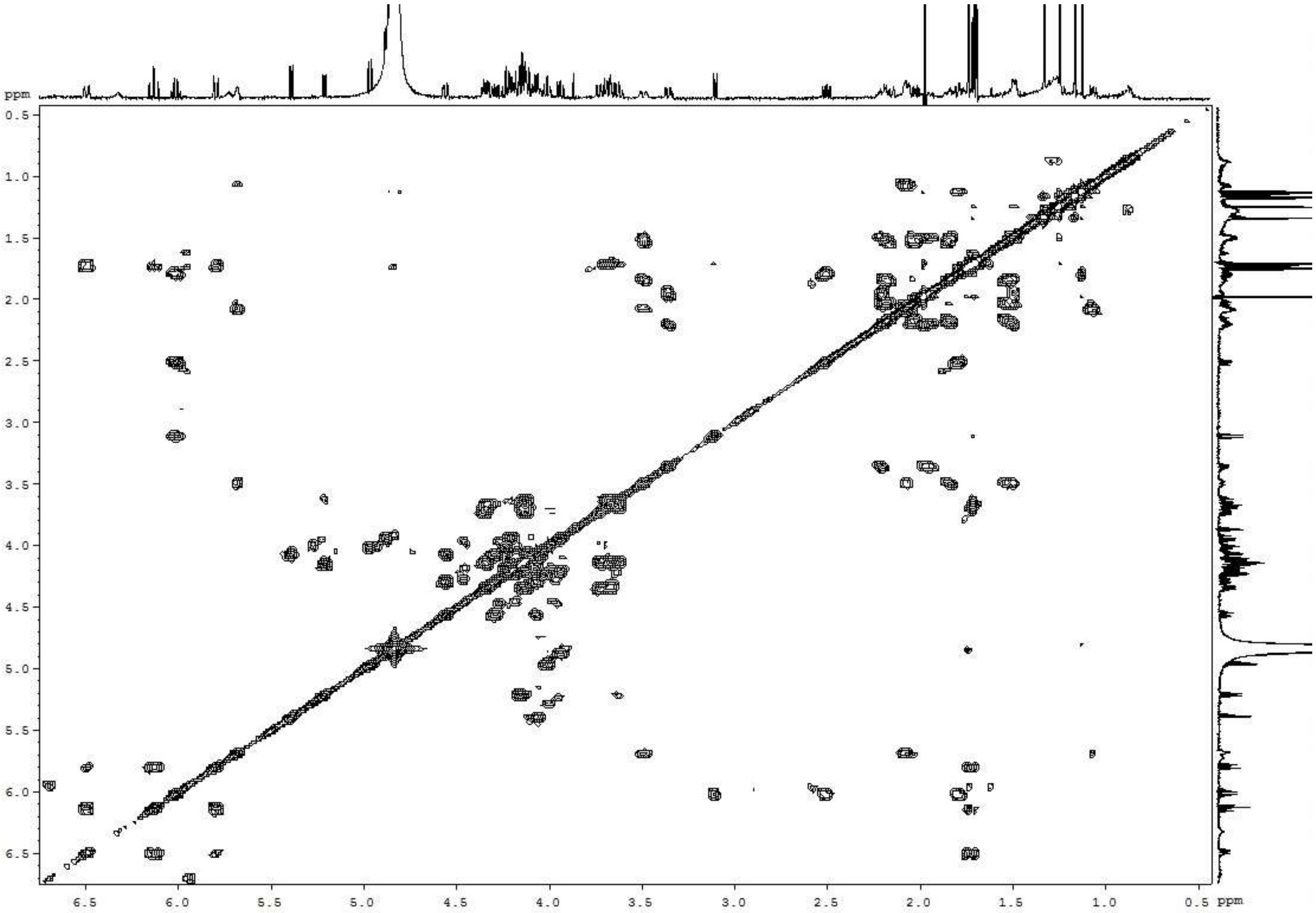

Figure S44. HSQC spectrum of pacificusoside G (**5**) in C_5D_5N.

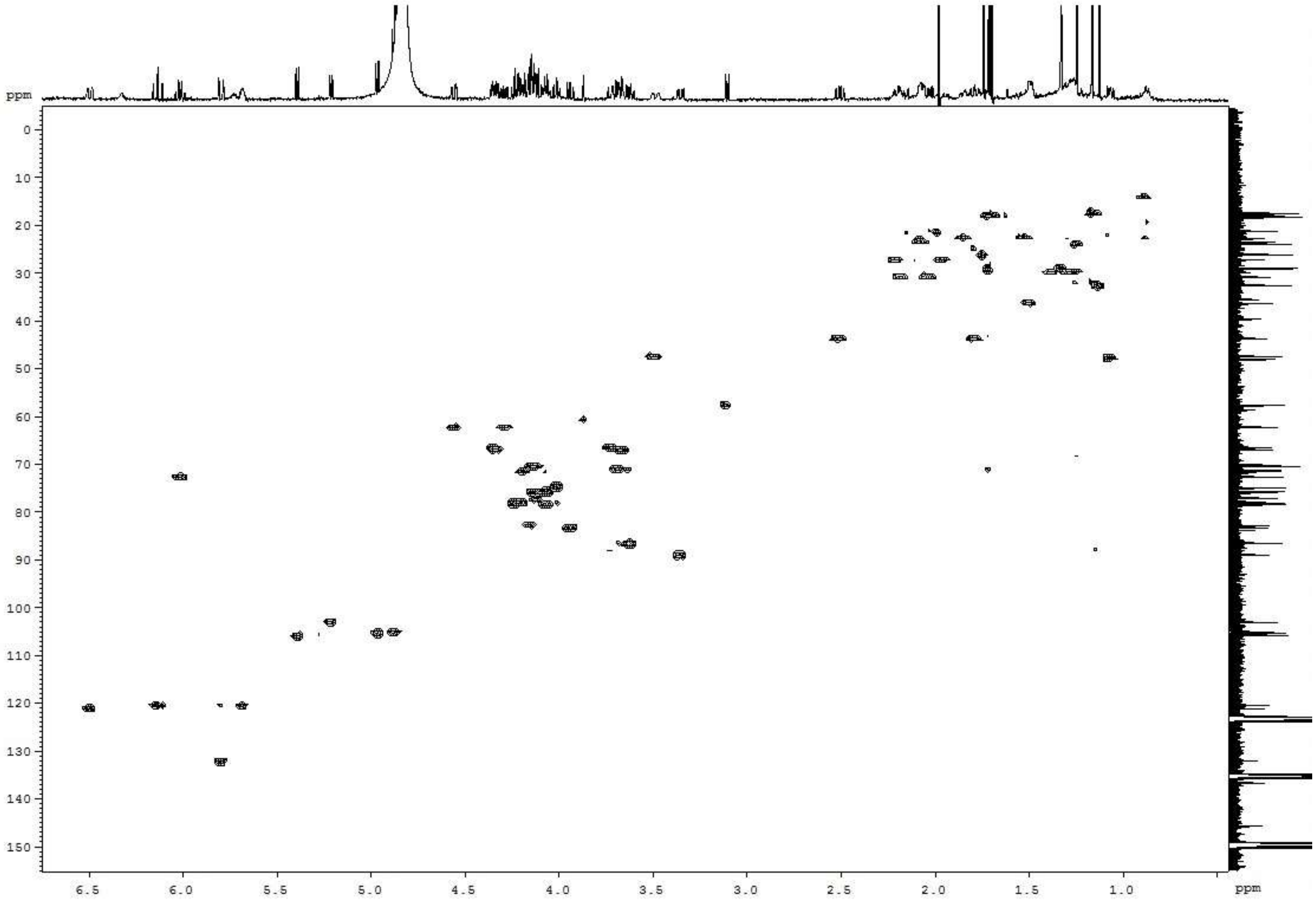

Figure S45. HMBC spectrum of pacificusoside G (**5**) in C_5D_5N.

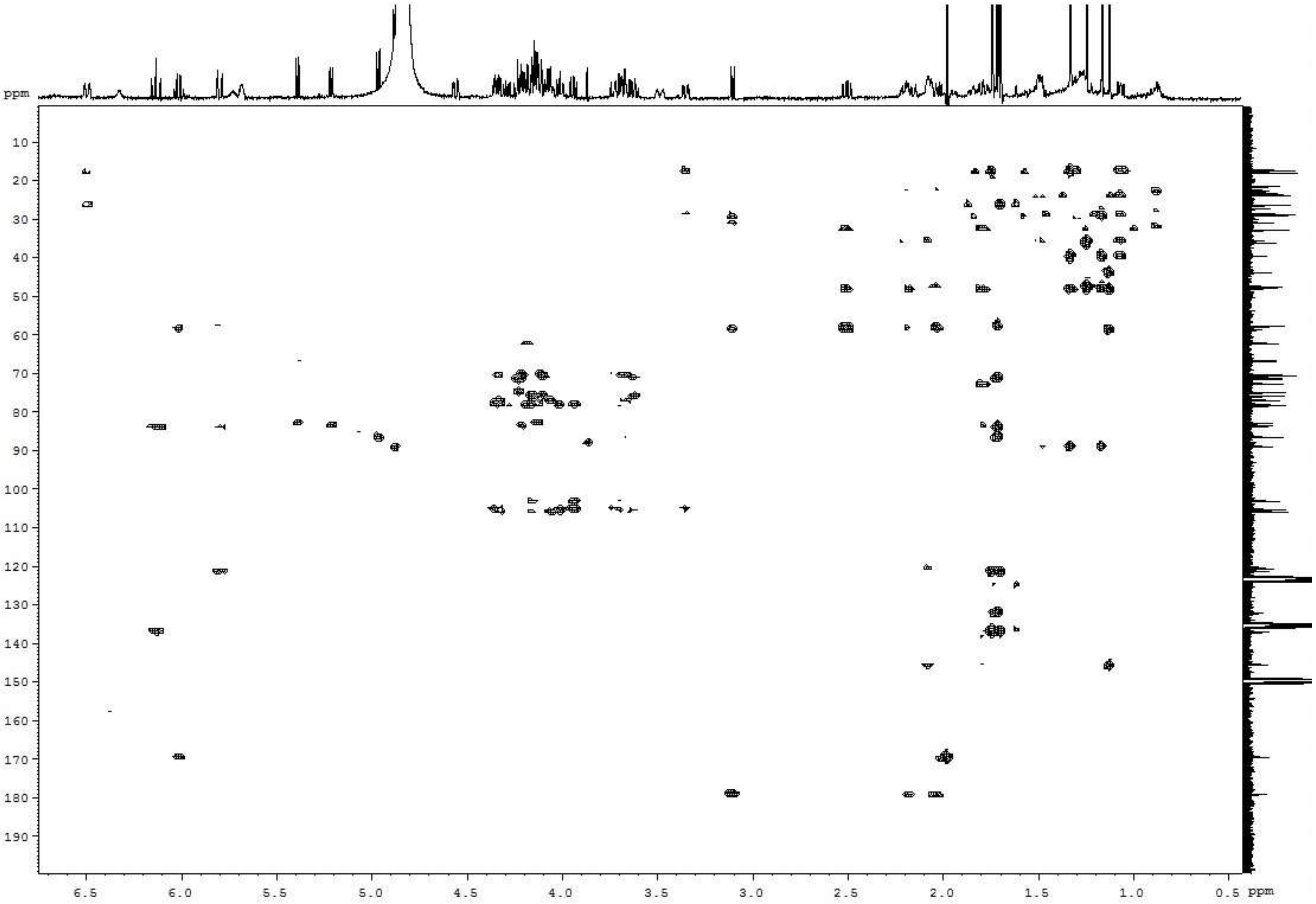

Figure S46. ROESY spectrum of pacificusoside G (**5**) in C_5D_5N.

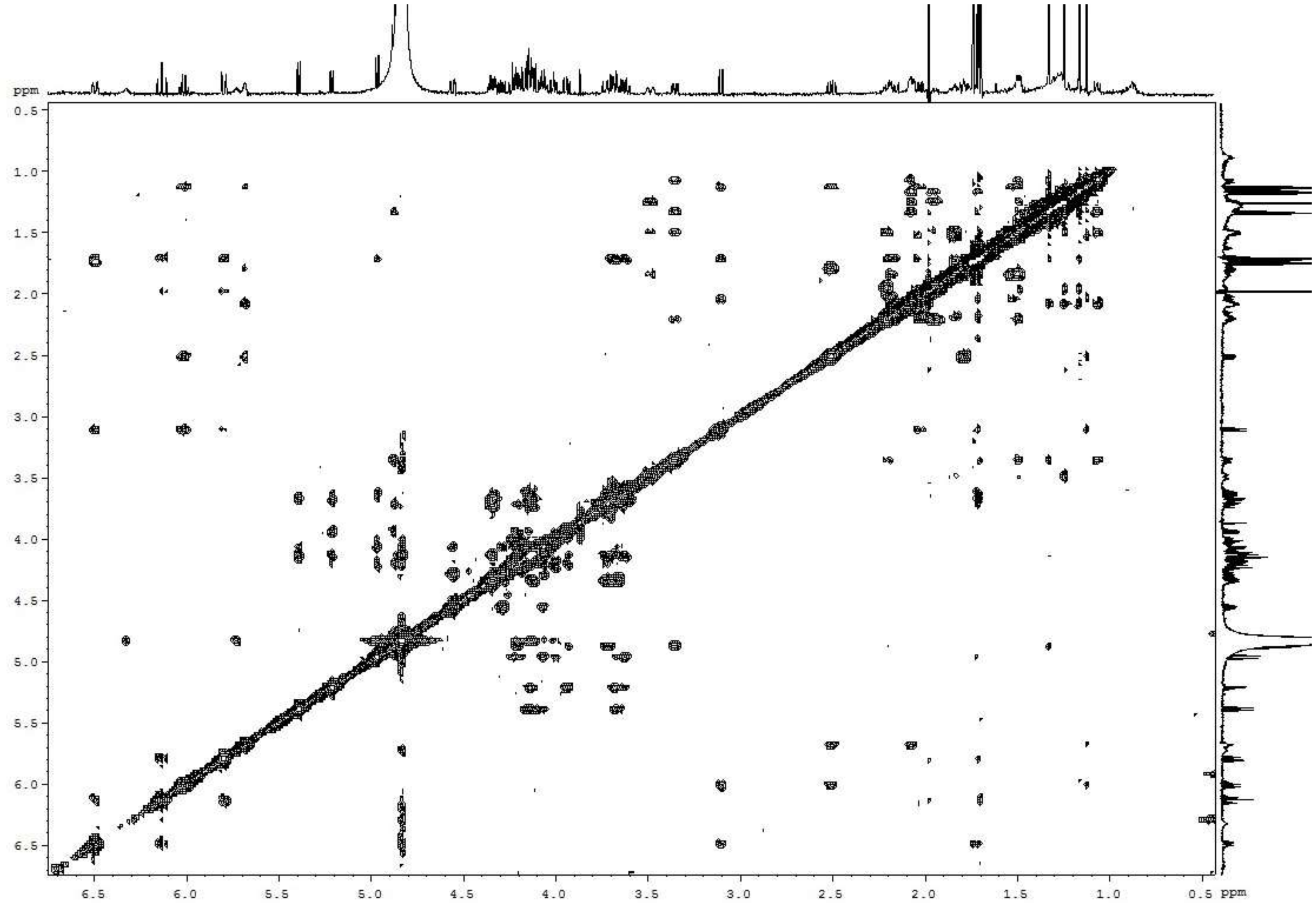

Figure S47. HRESIMS spectrum of pacificusoside H (**6**).

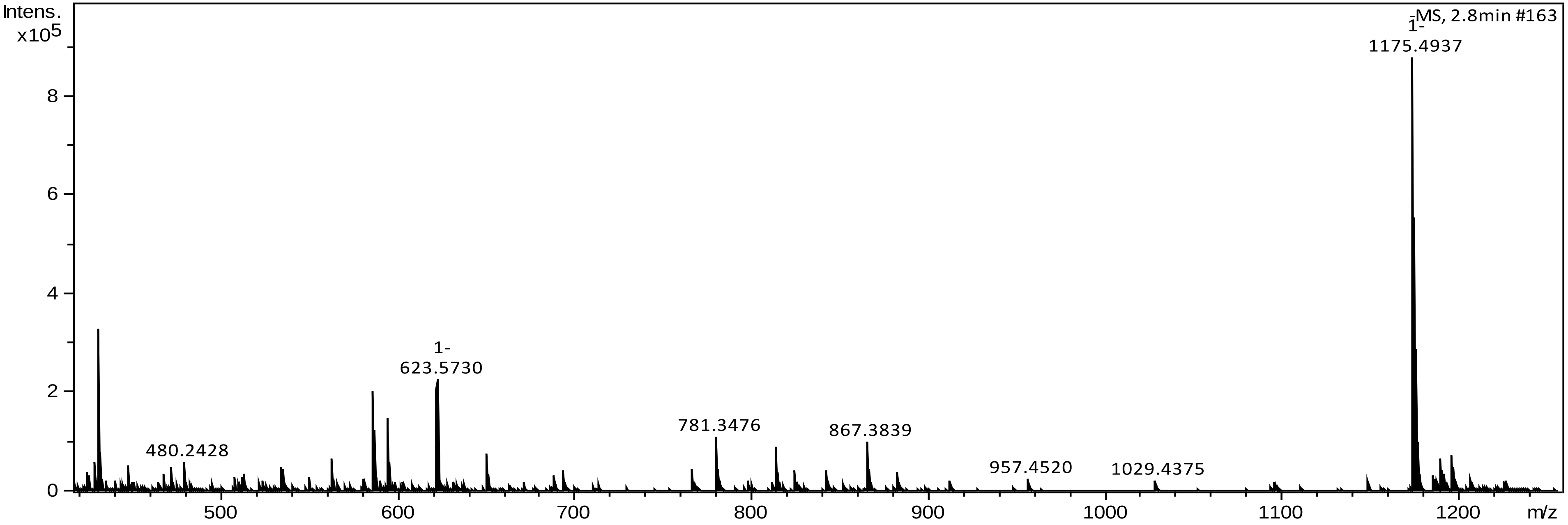

Figure S48. IR spectrum of pacificusoside H (**6**) in KBr.

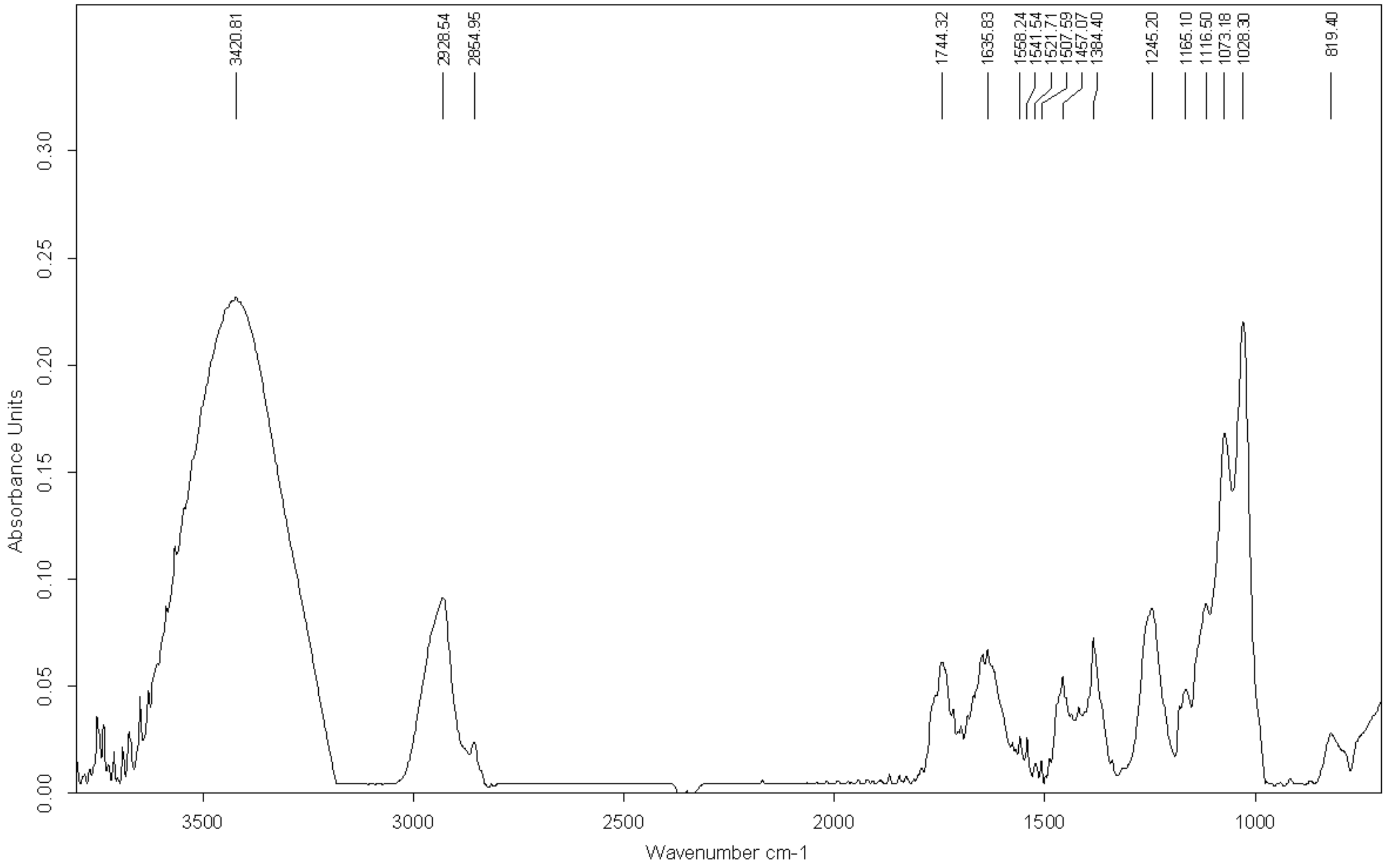

Figure S49. ^{1}H-NMR spectrum of pacificusoside H (**6**) in C_5D_5N.

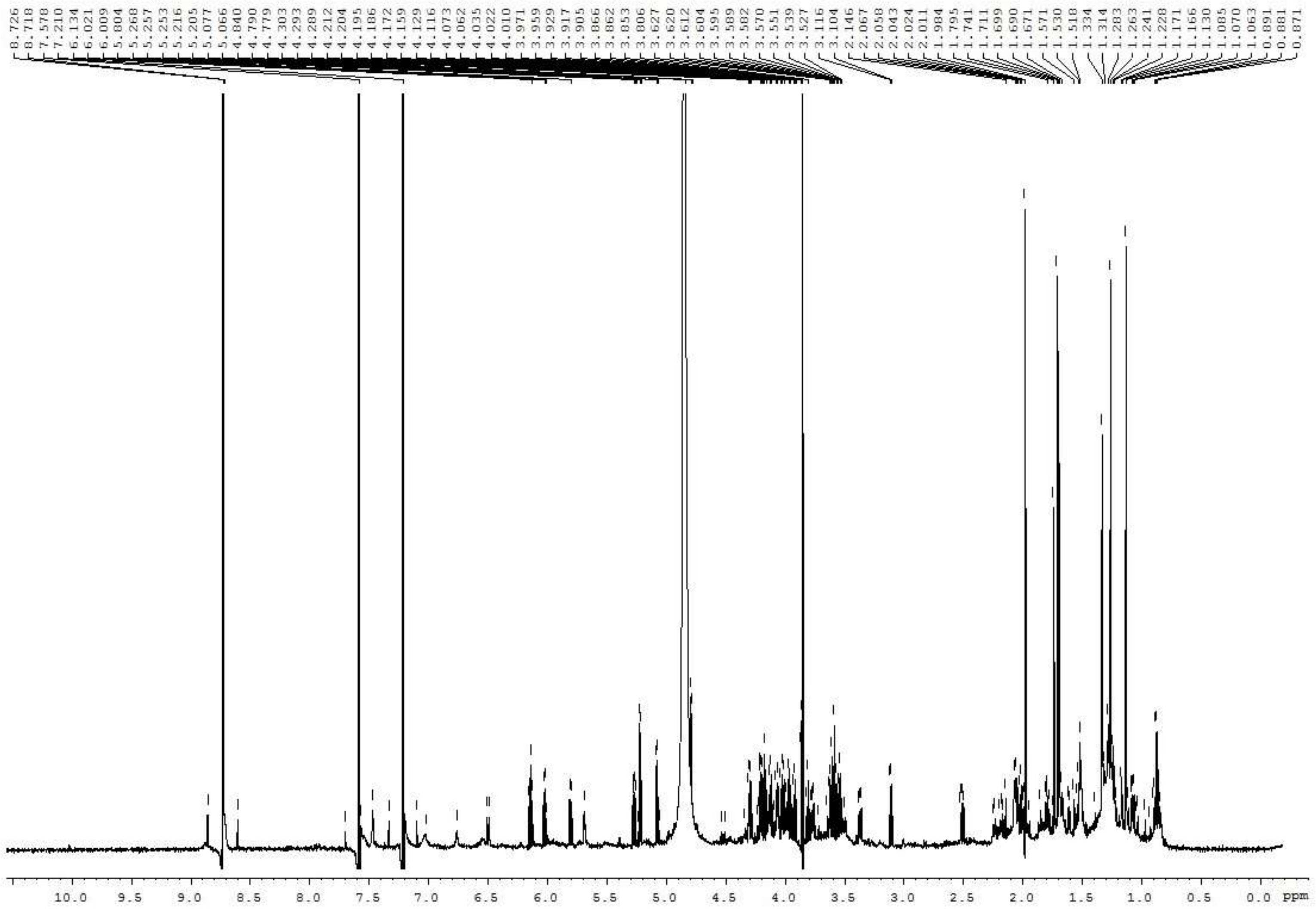

Figure S50. ^{13}C-NMR spectrum of pacificusoside H (**6**) in C_5D_5N.

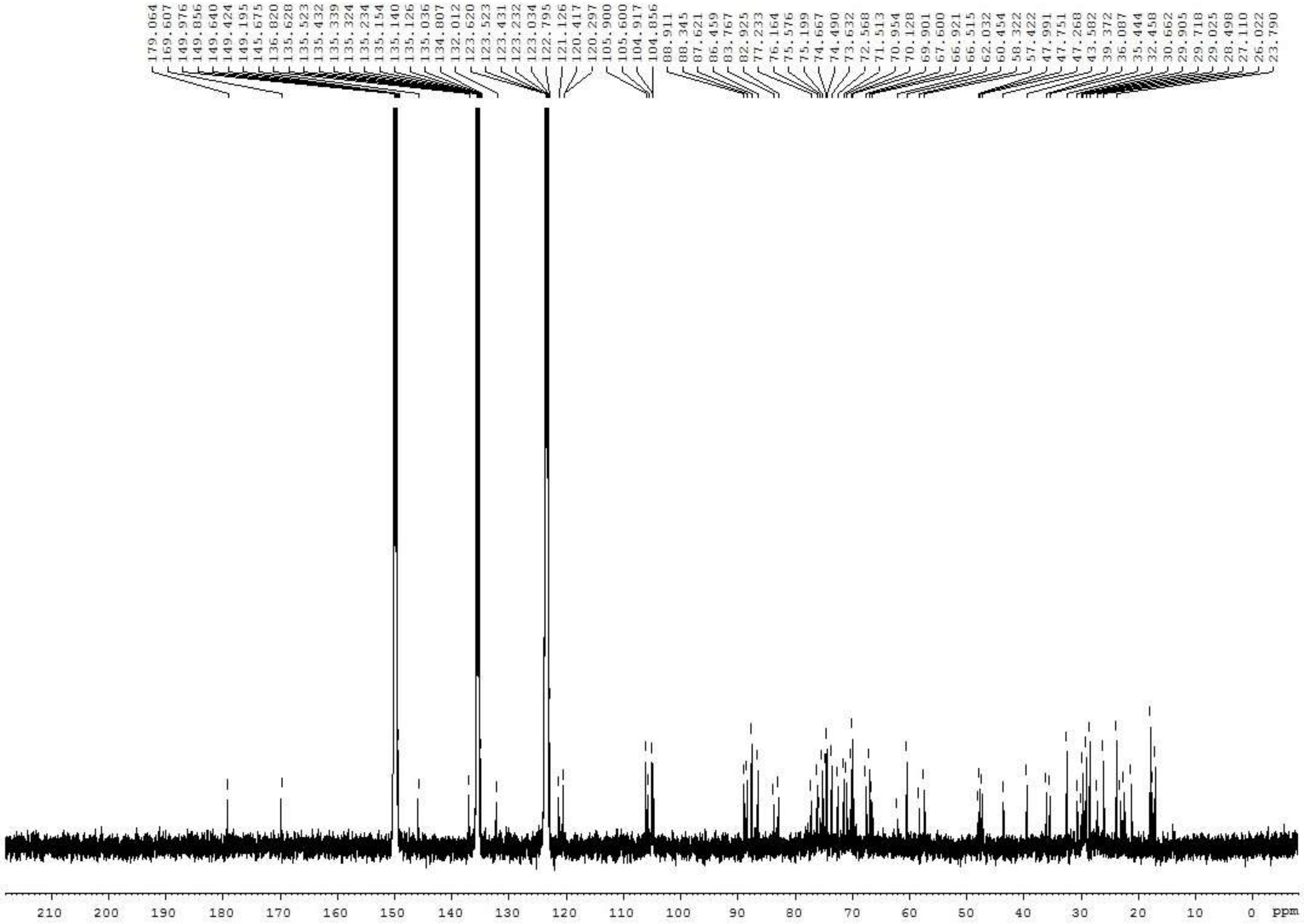

Figure S51. ^{1}H-^{1}H COSY spectrum of pacificusoside H (**6**) in C_5D_5N.

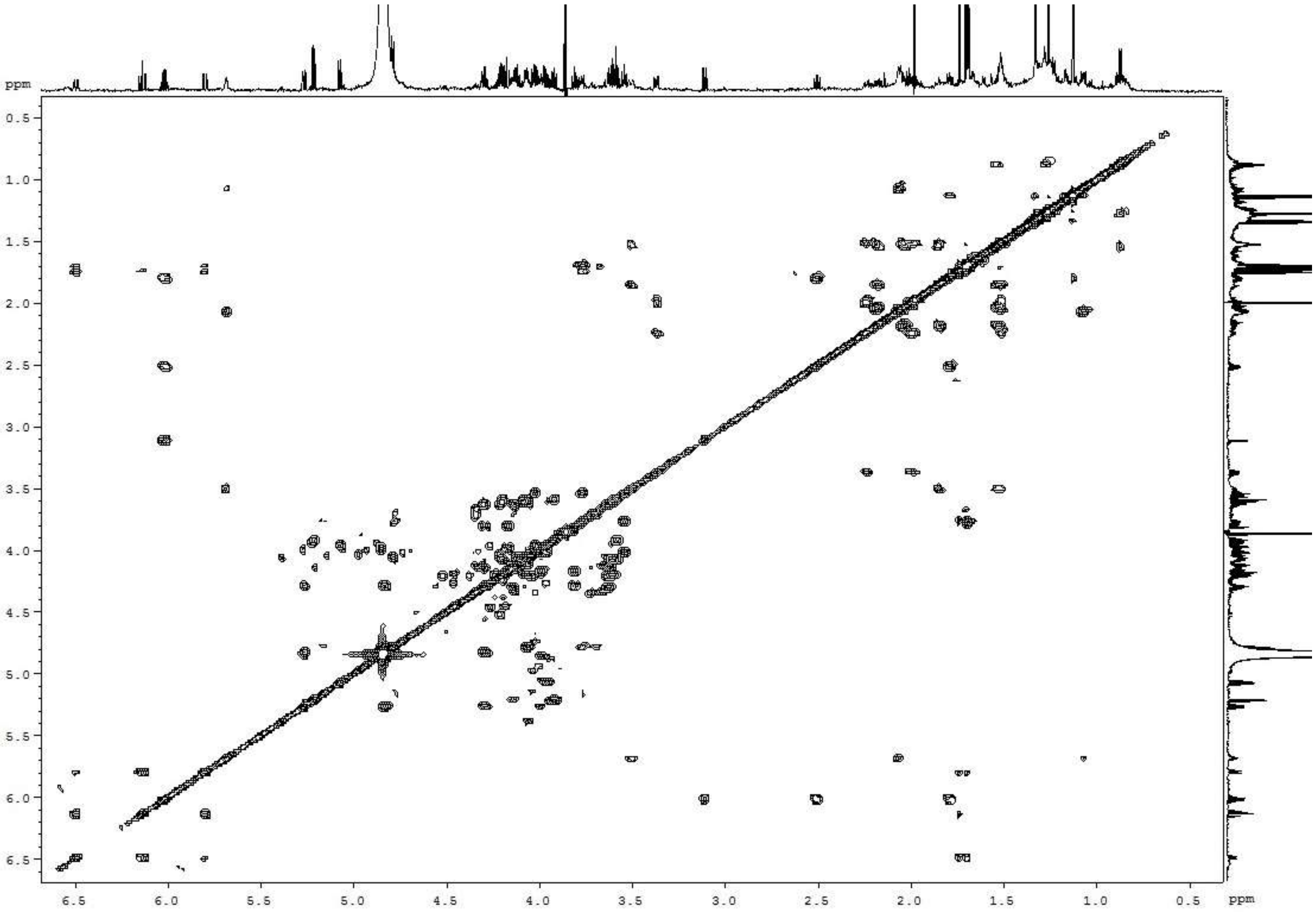

Figure S52. HSQC spectrum of pacificusoside H (**6**) in C_5D_5N.

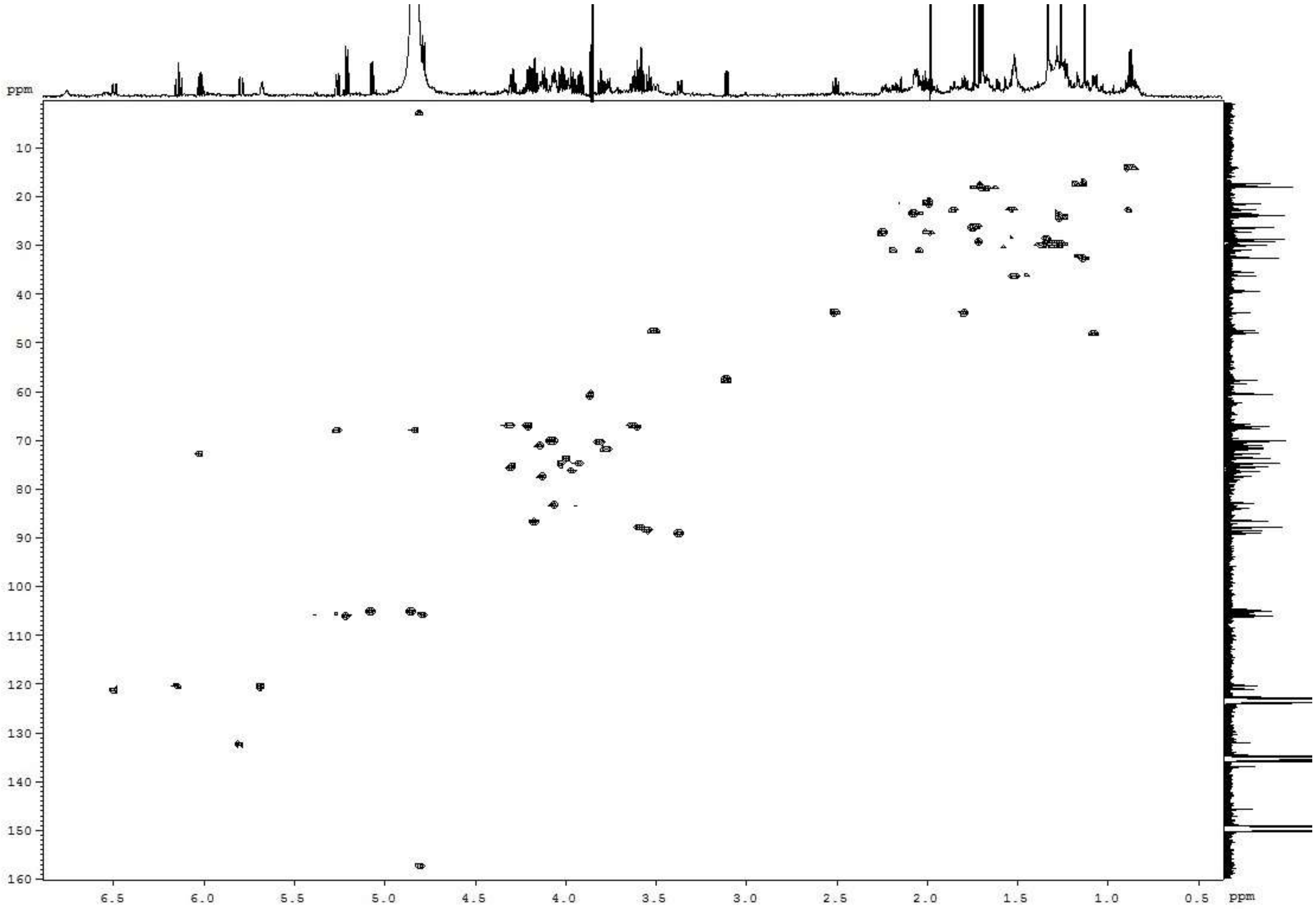

Figure S53. HMBC spectrum of pacificusoside H (**6**) in C_5D_5N.

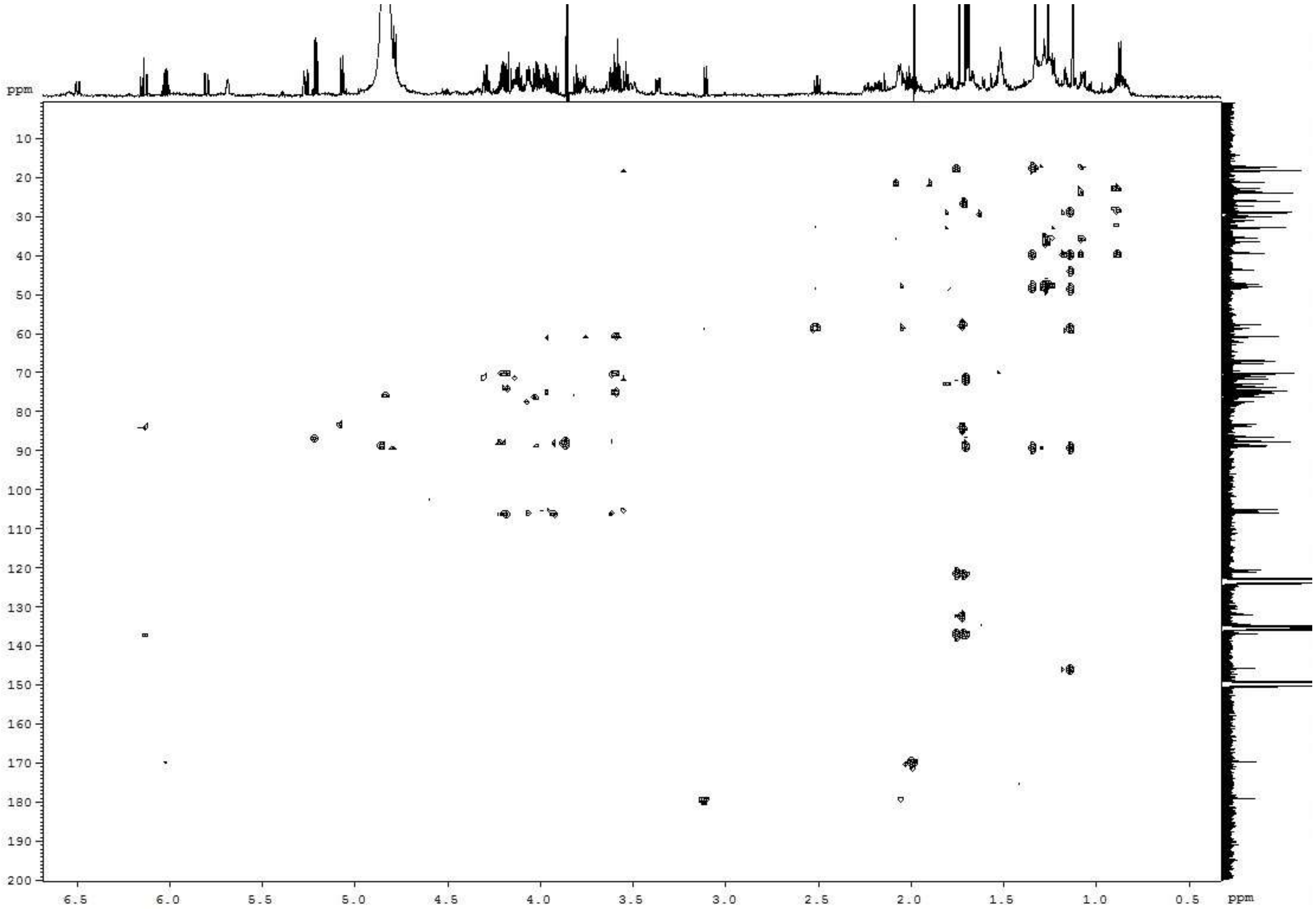

Figure S54. ROESY spectrum of pacificusoside H (**6**) in C_5D_5N.

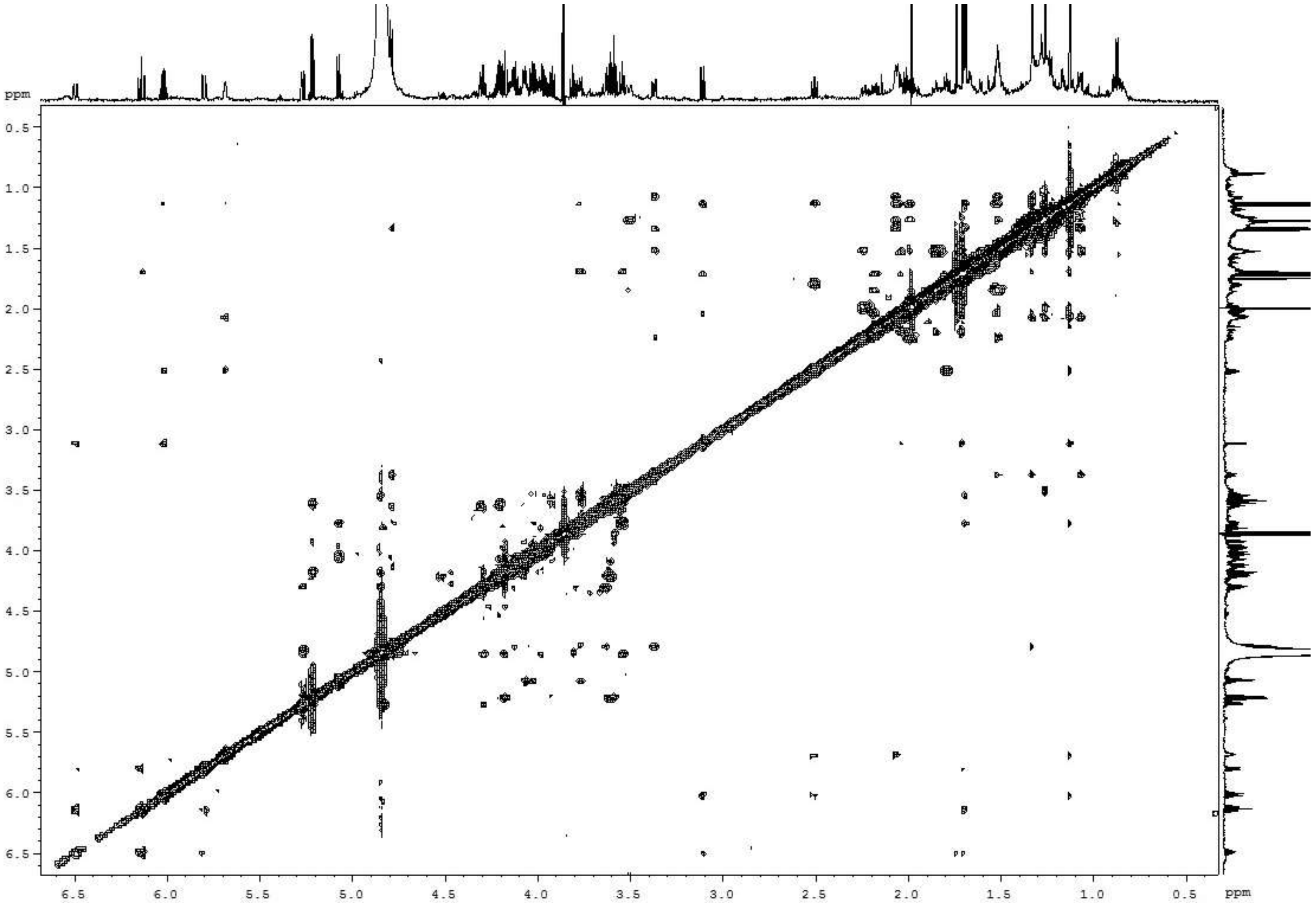

Figure S55. HRESIMS spectrum of pacificusoside I (**7**).

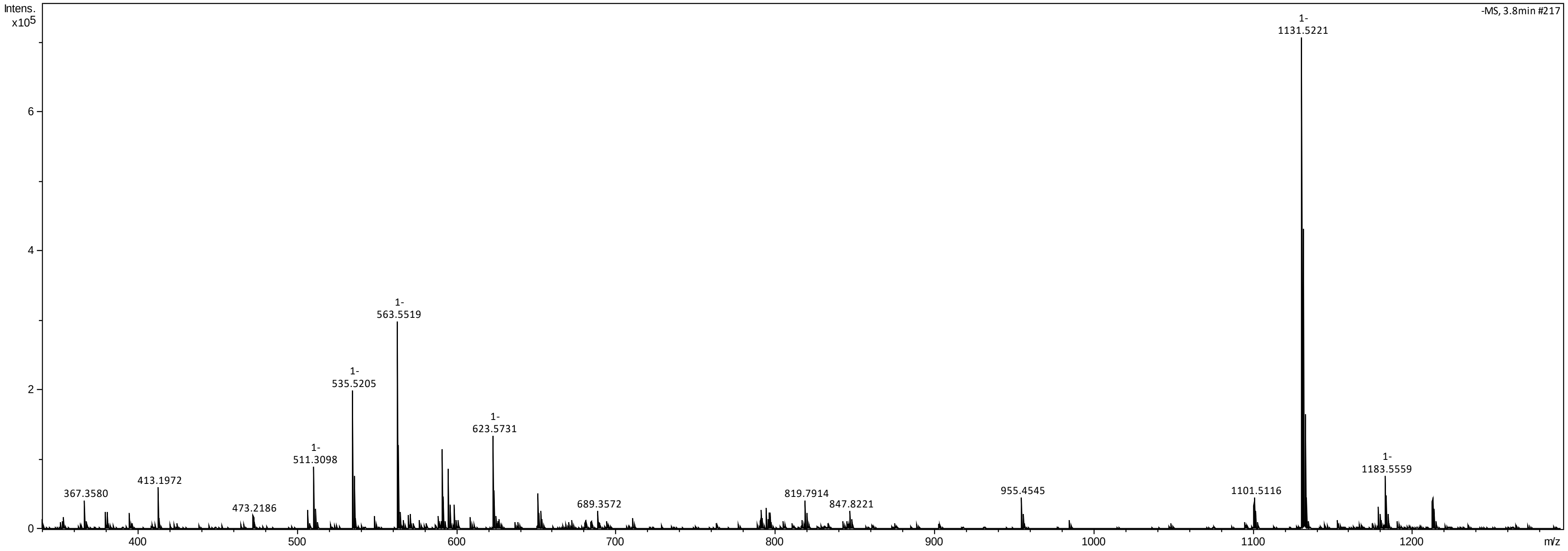

Figure S56. IR spectrum of pacificusoside I (**7**) in KBr.

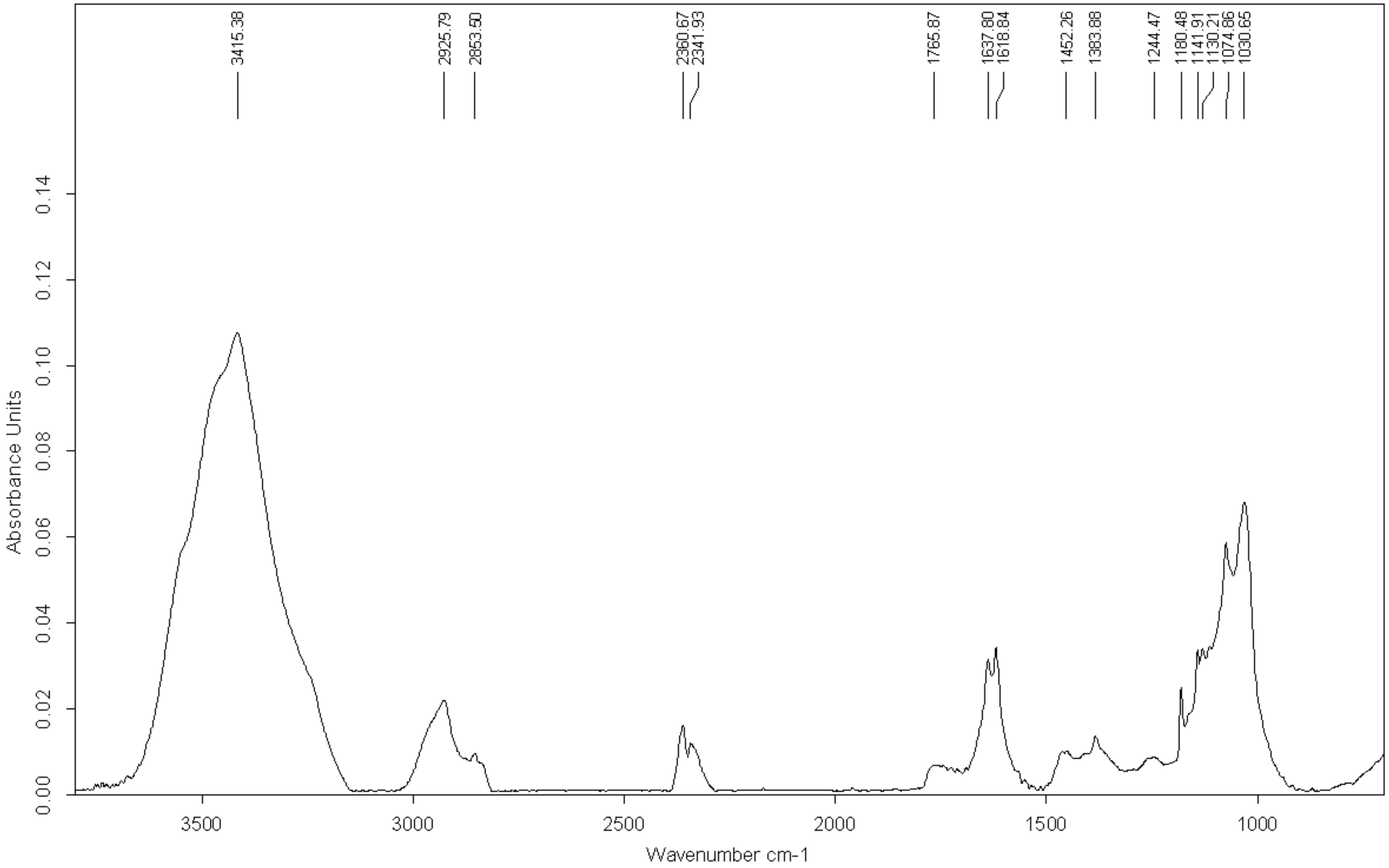

Figure S57. ^{1}H-NMR spectrum of pacificusoside I (**7**) in C_5D_5N.

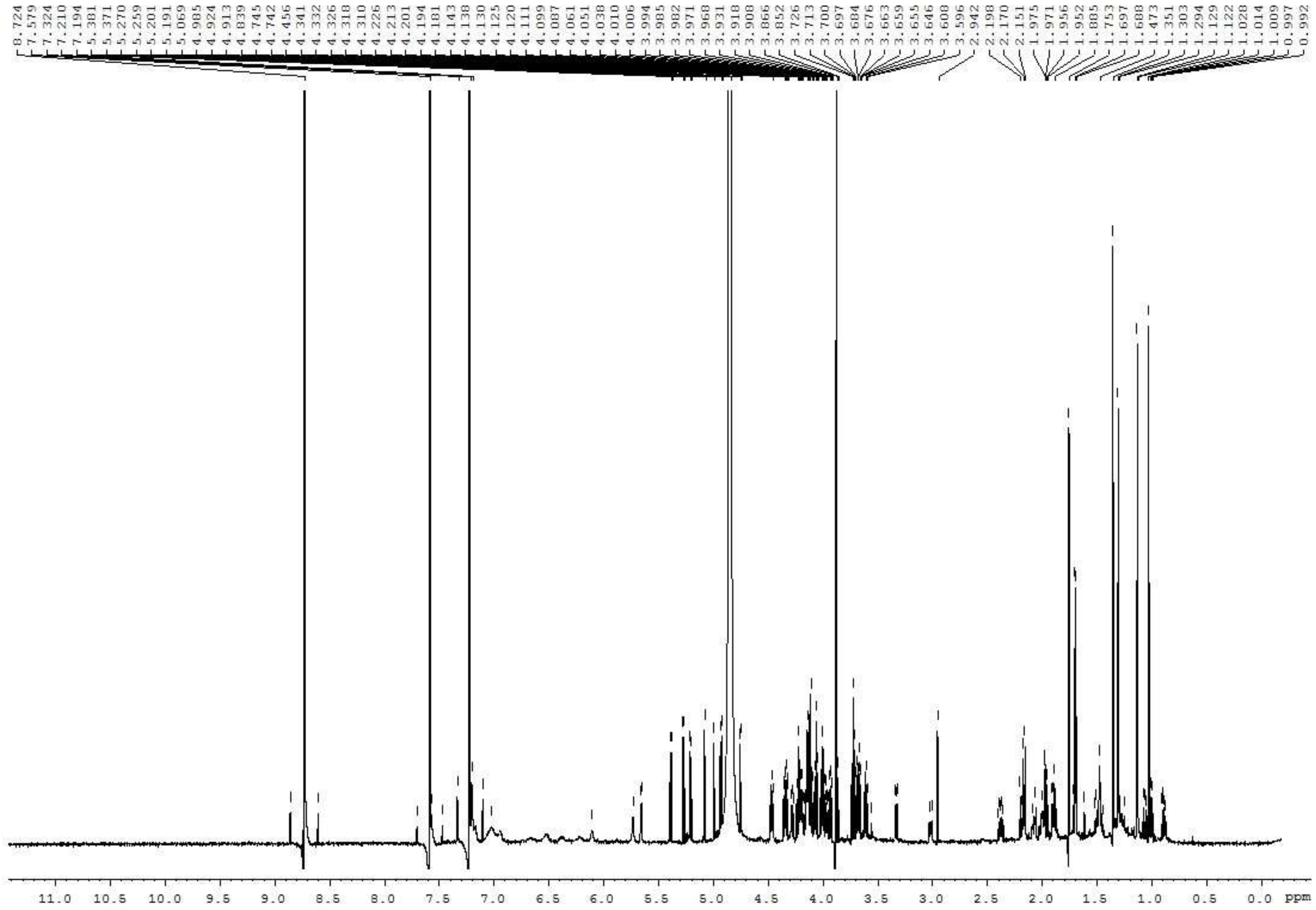

Figure S58. ^{13}C-NMR spectrum of pacificusoside I (**7**) in C_5D_5N.

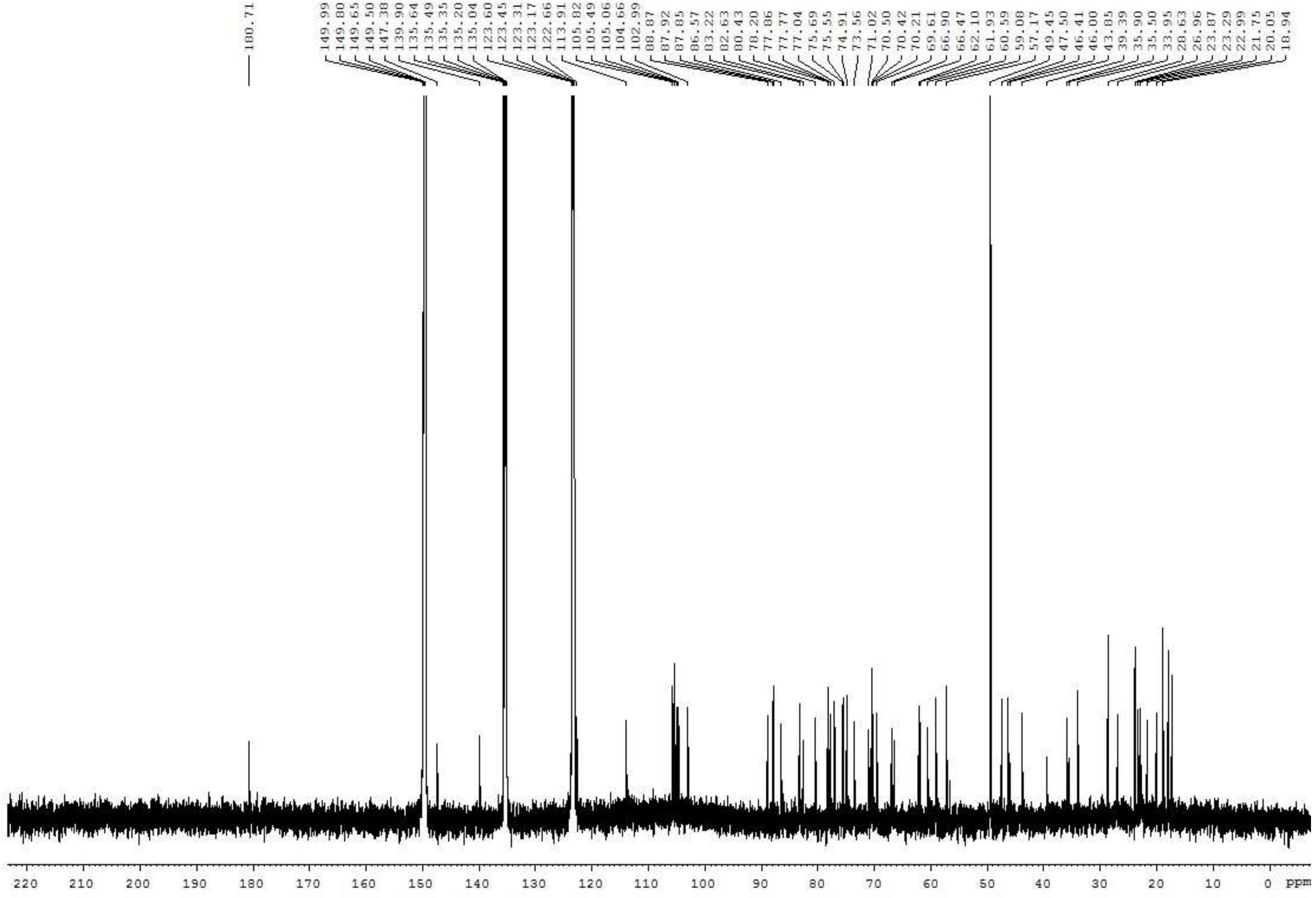

Figure S59. ^1H-^1H COSY spectrum of pacificusoside I (**7**) in C_5D_5N.

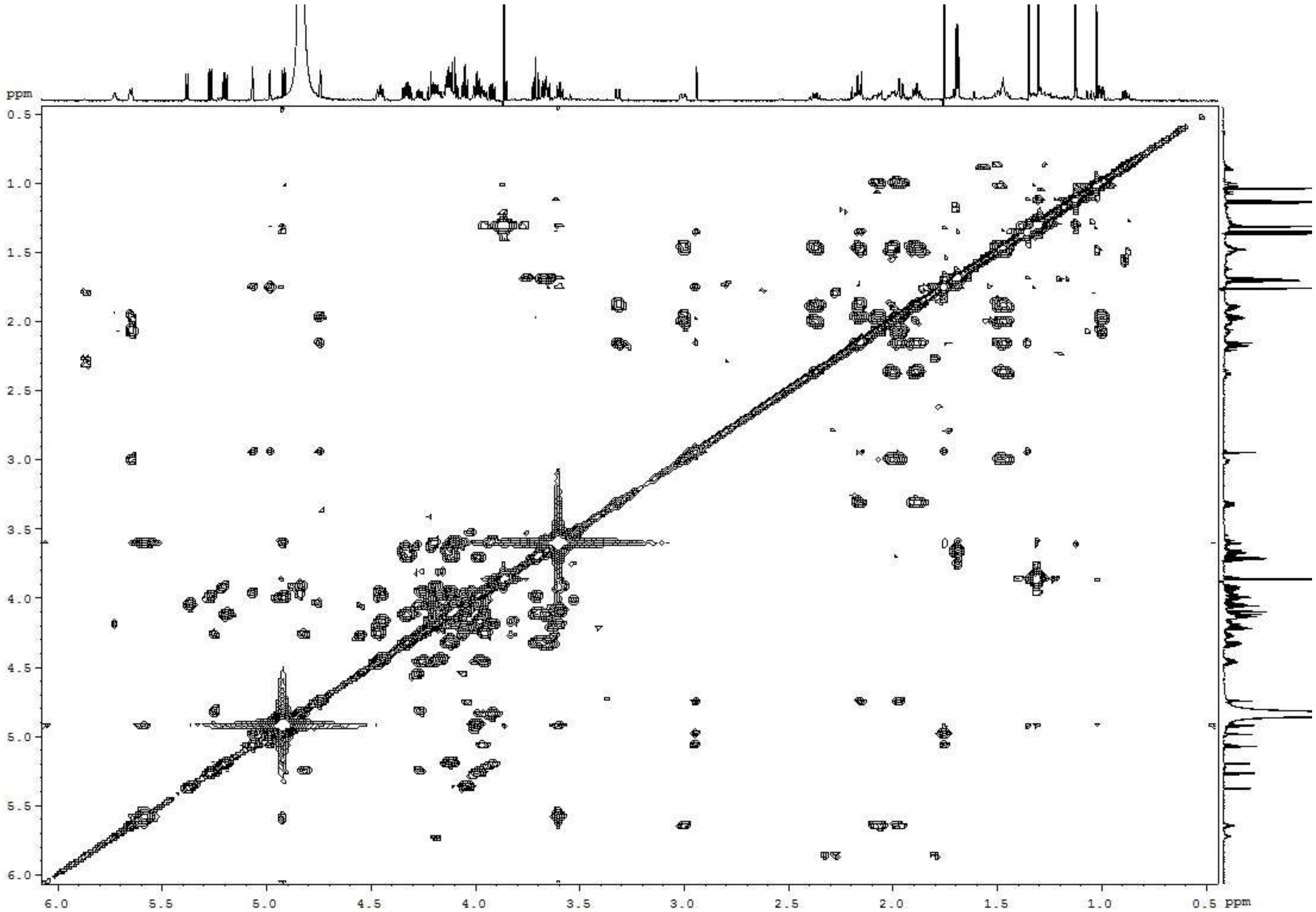

Figure S60. HSQC spectrum of pacificusoside I (**7**) in C_5D_5N.

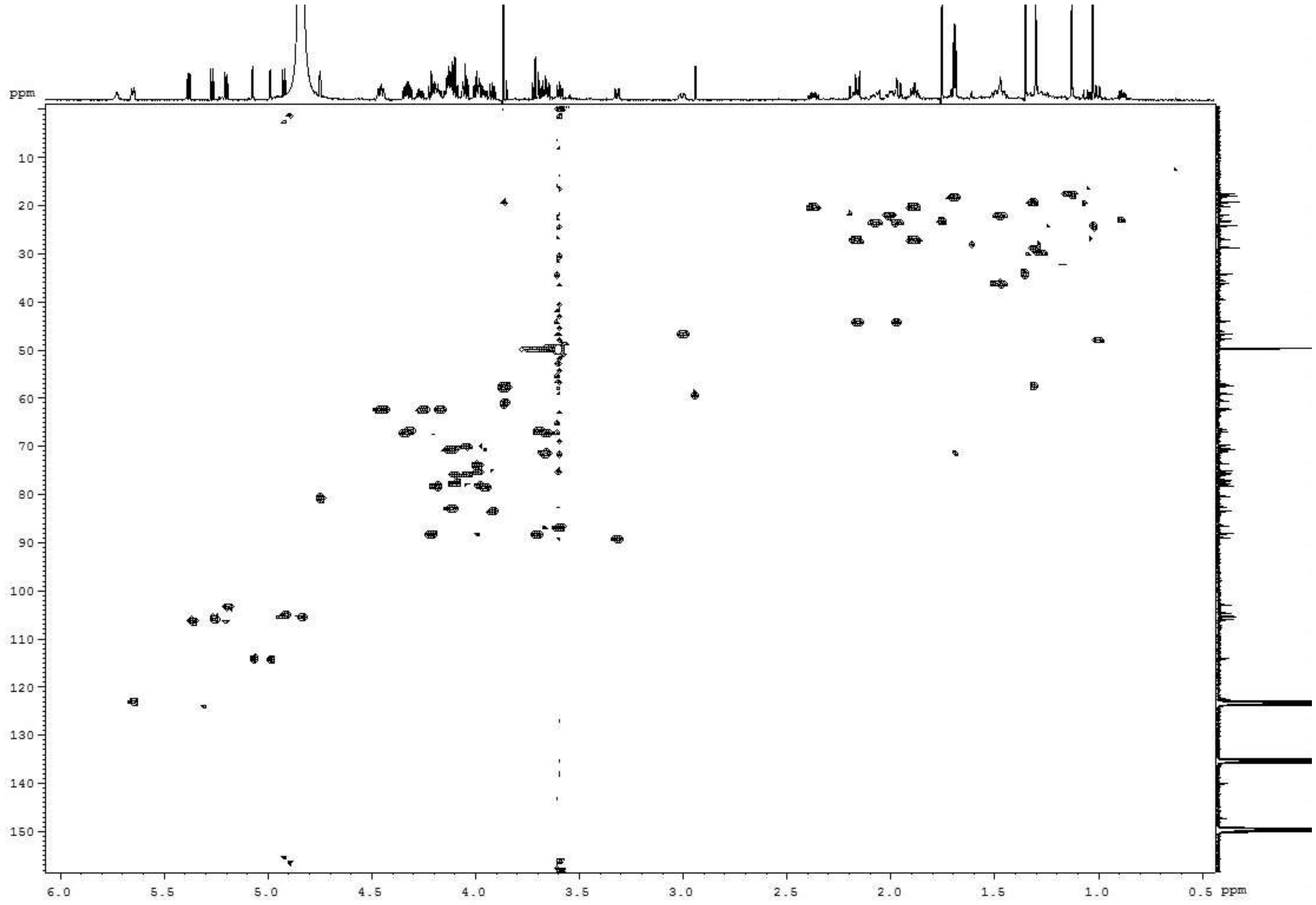

Figure S61. HMBC spectrum of pacificusoside I (**7**) in C_5D_5N.

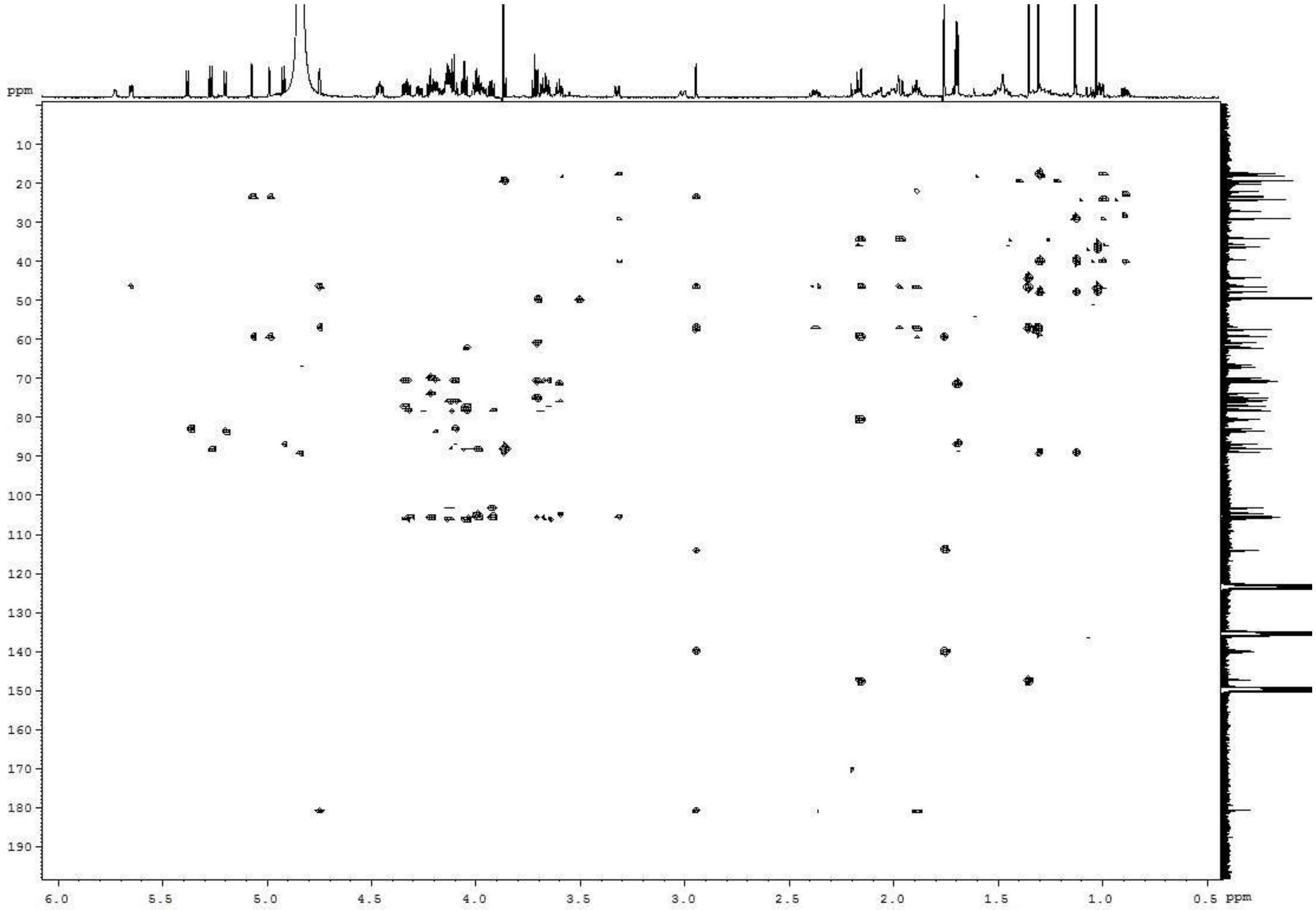

Figure S62. ROESY spectrum of pacificusoside I (**7**) in C_5D_5N.

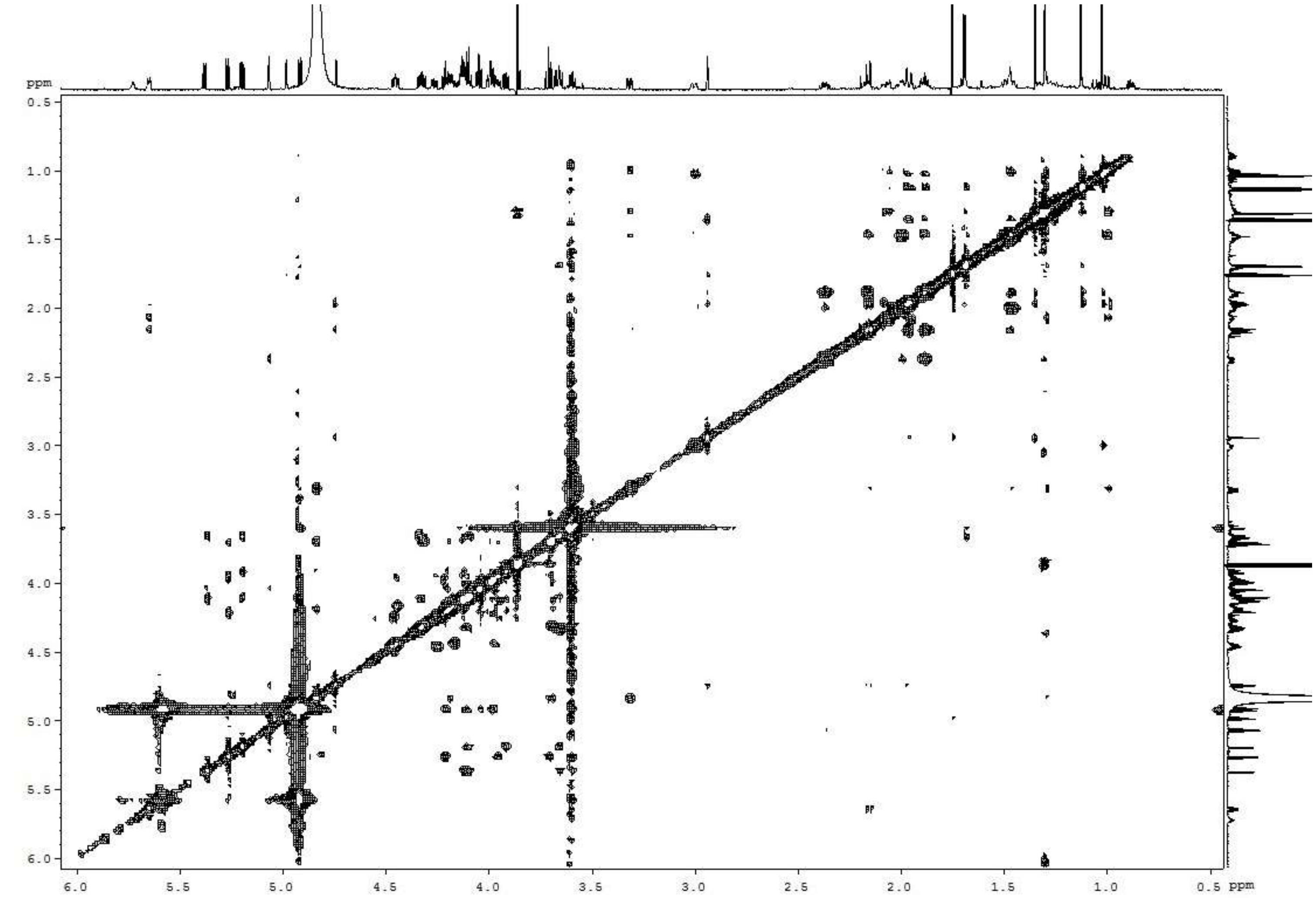

Figure S63. HRESIMS spectrum of pacificusoside J (**8**).

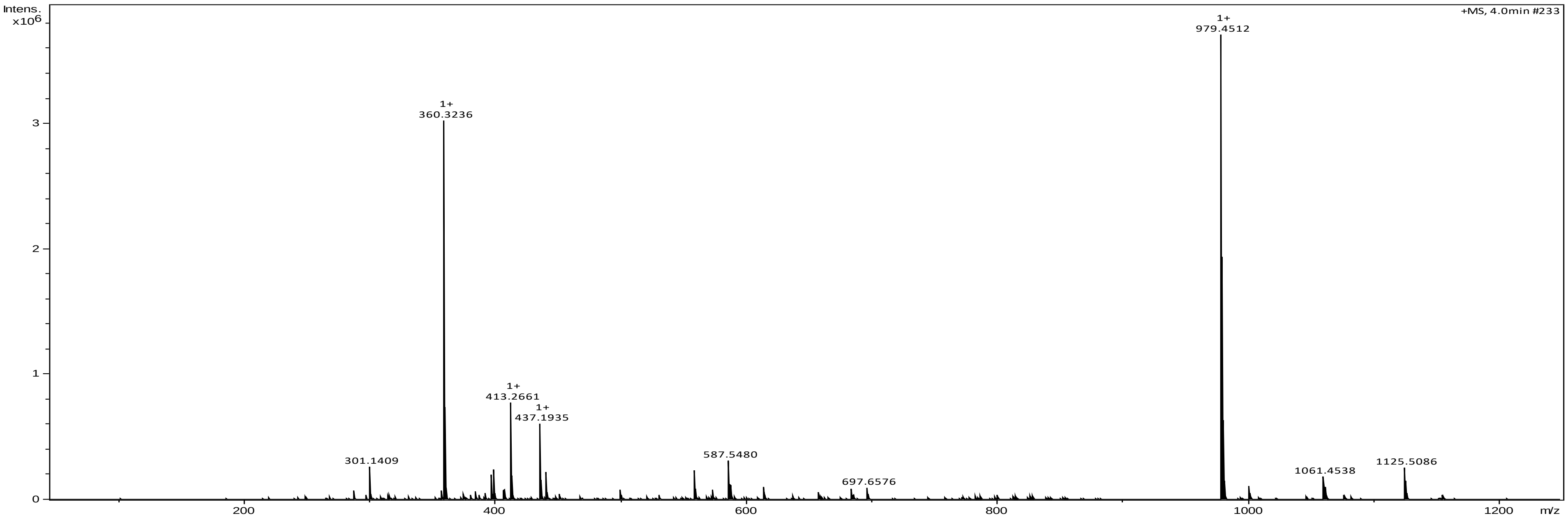

Figure S64. IR spectrum of pacificusoside J (**8**) in KBr.

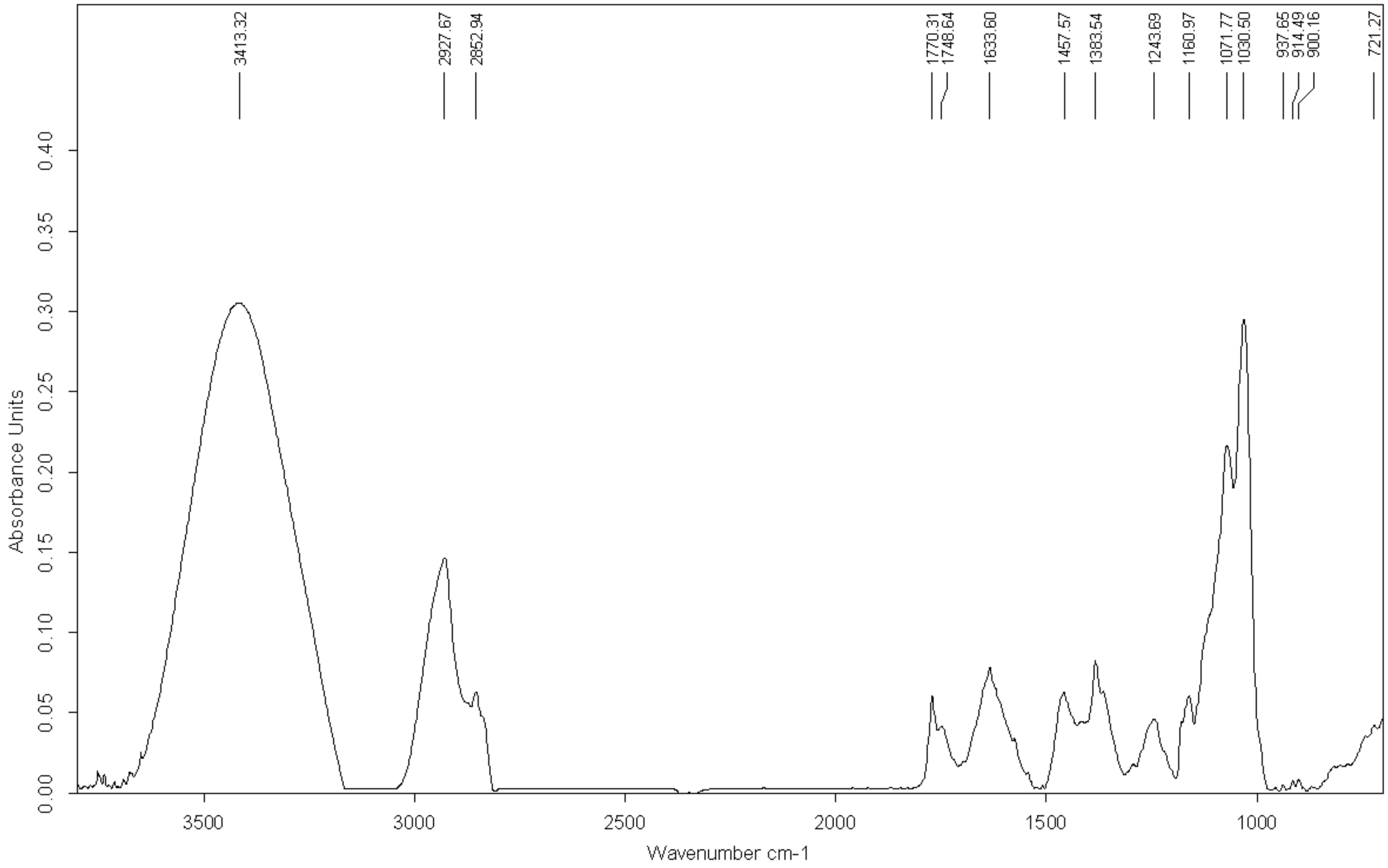

Figure S65. ^{1}H-NMR spectrum of pacificusoside J (**8**) in C_5D_5N.

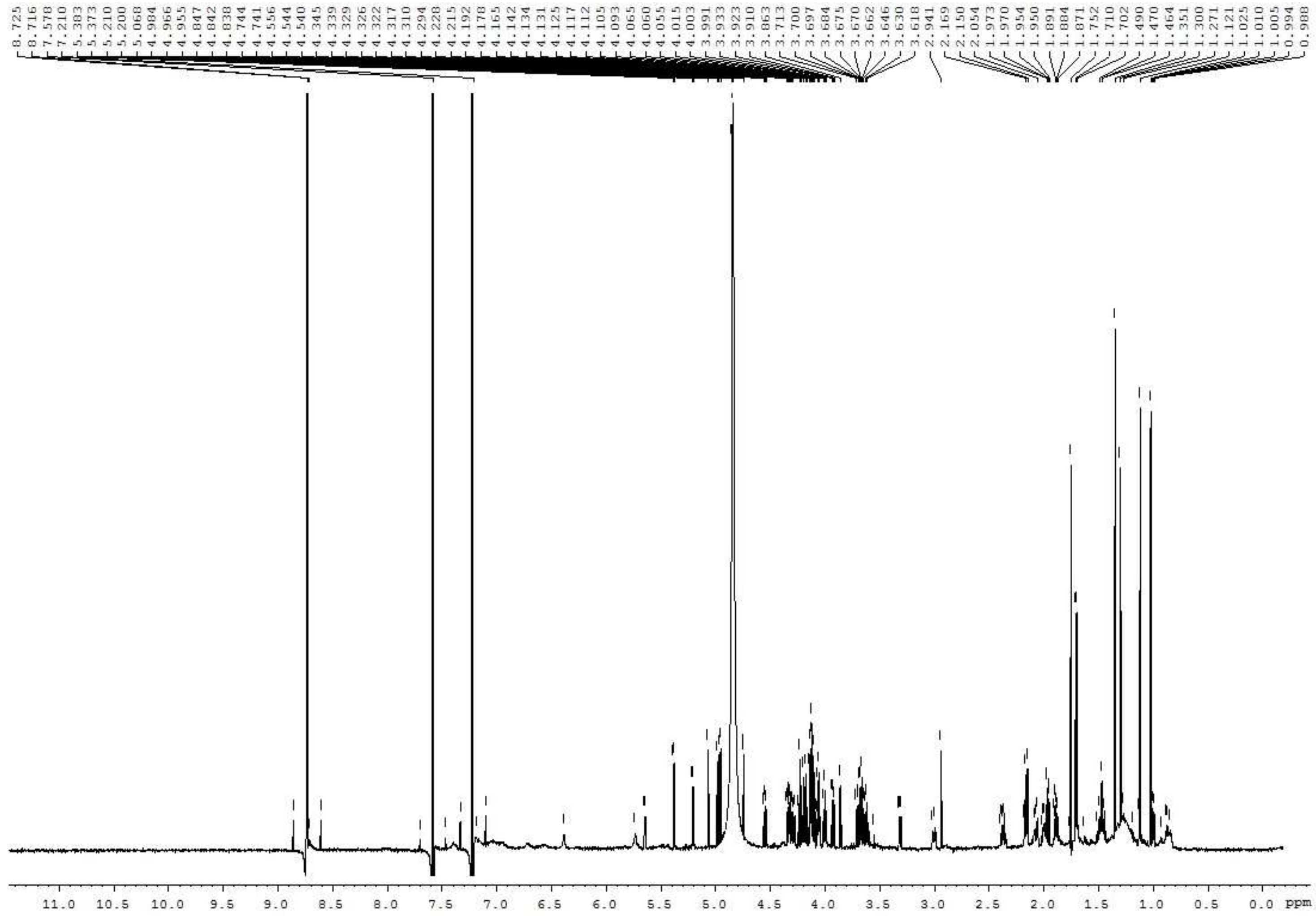

Figure S66. ^{13}C-NMR spectrum of pacificusoside J (**8**) in C_5D_5N.

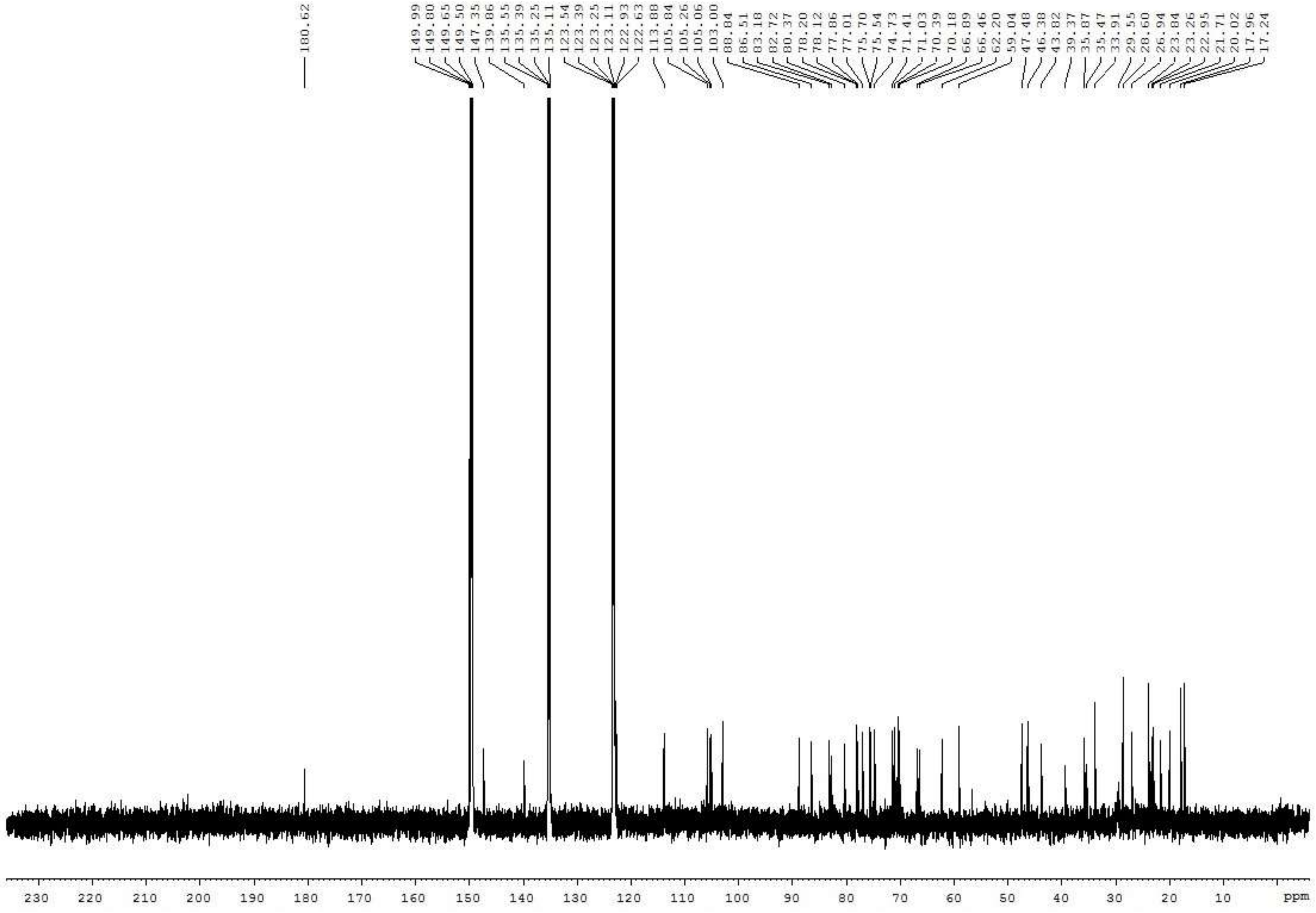

Figure S67. ^1H-^1H COSY spectrum of pacificusoside J (**8**) in C_5D_5N.

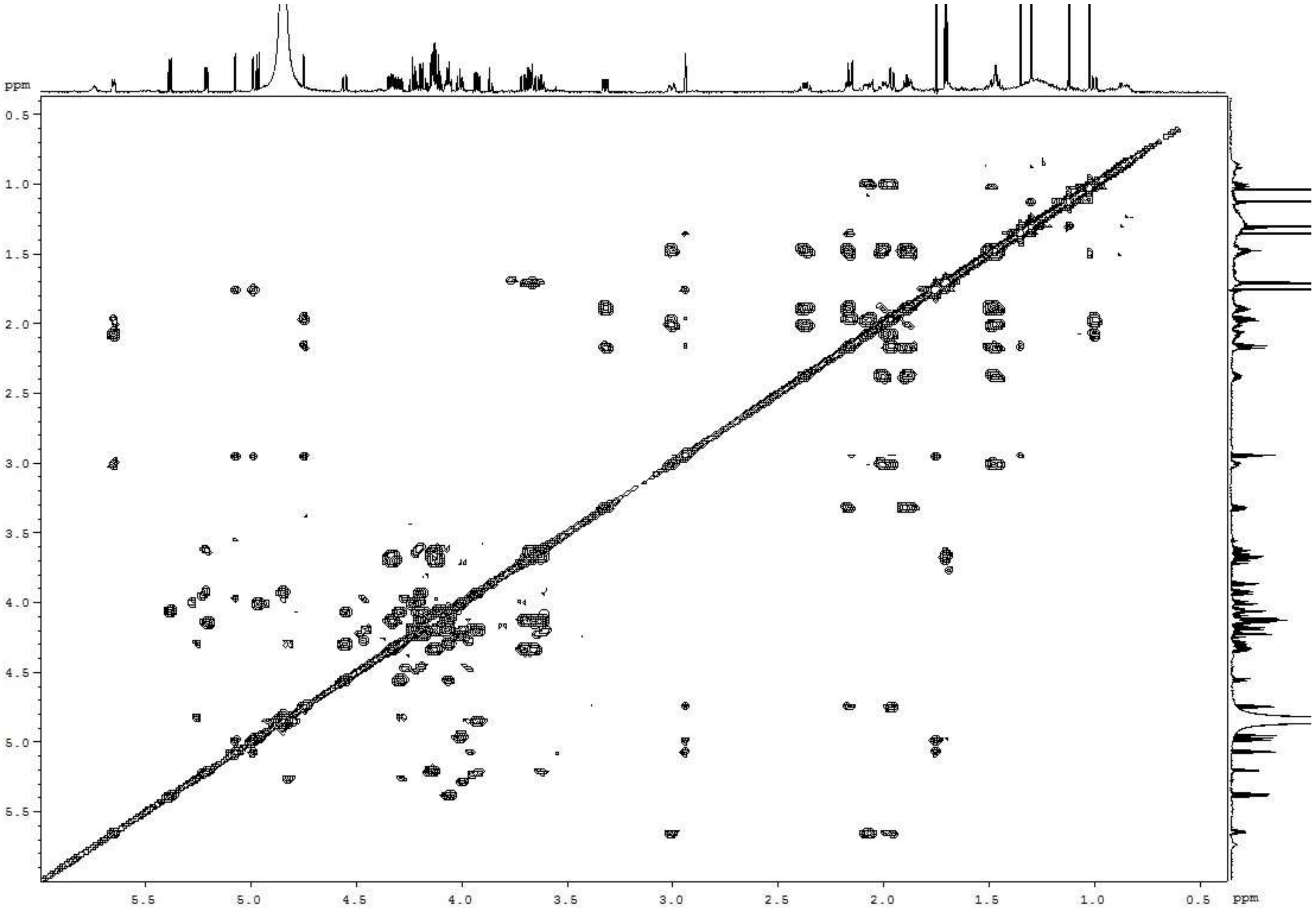

Figure S68. HSQC spectrum of pacificusoside J (**8**) in C_5D_5N.

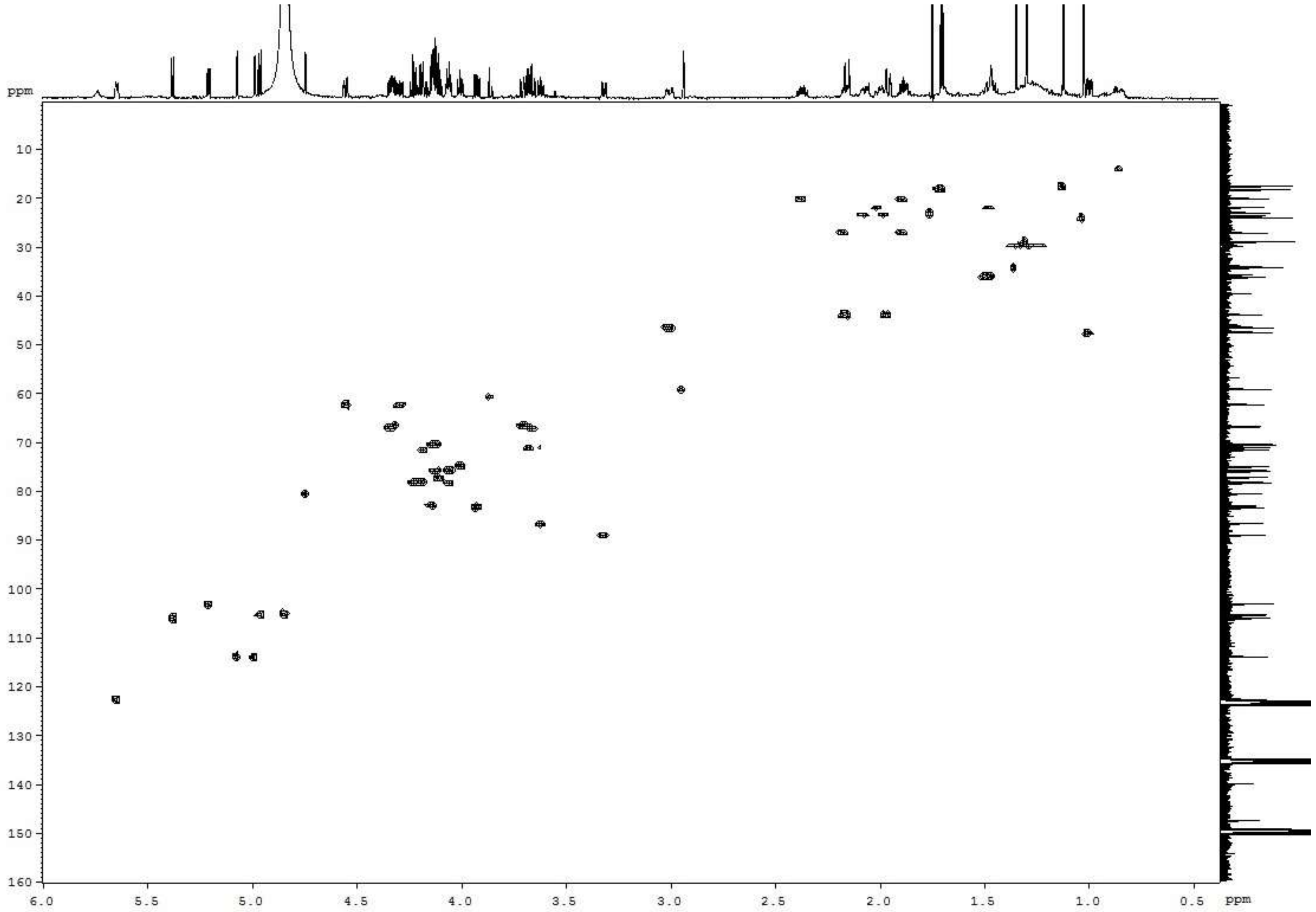

Figure S69. HMBC spectrum of pacificusoside J (**8**) in C_5D_5N.

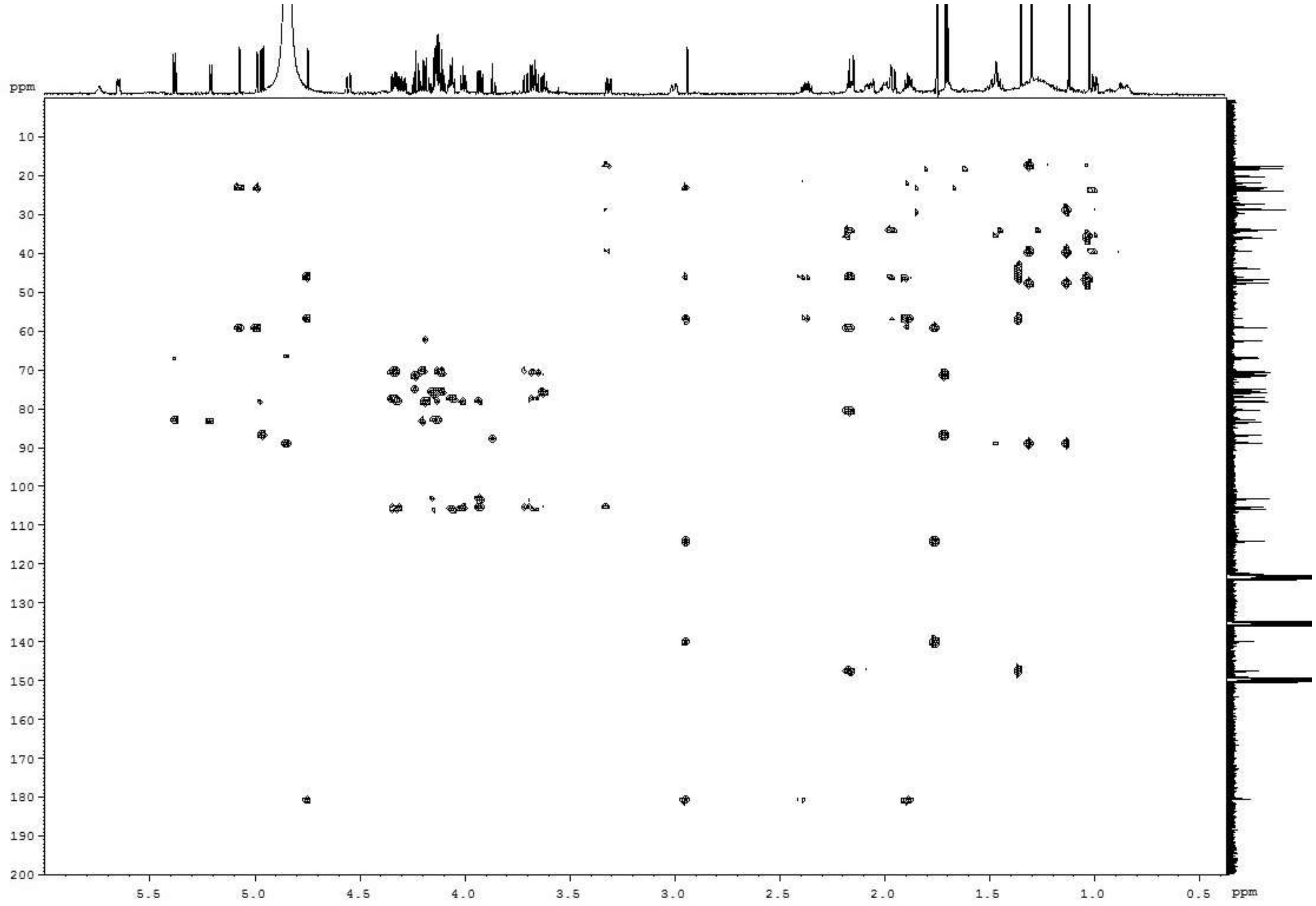

Figure S70. ROESY spectrum of pacificusoside J (**8**) in C_5D_5N.

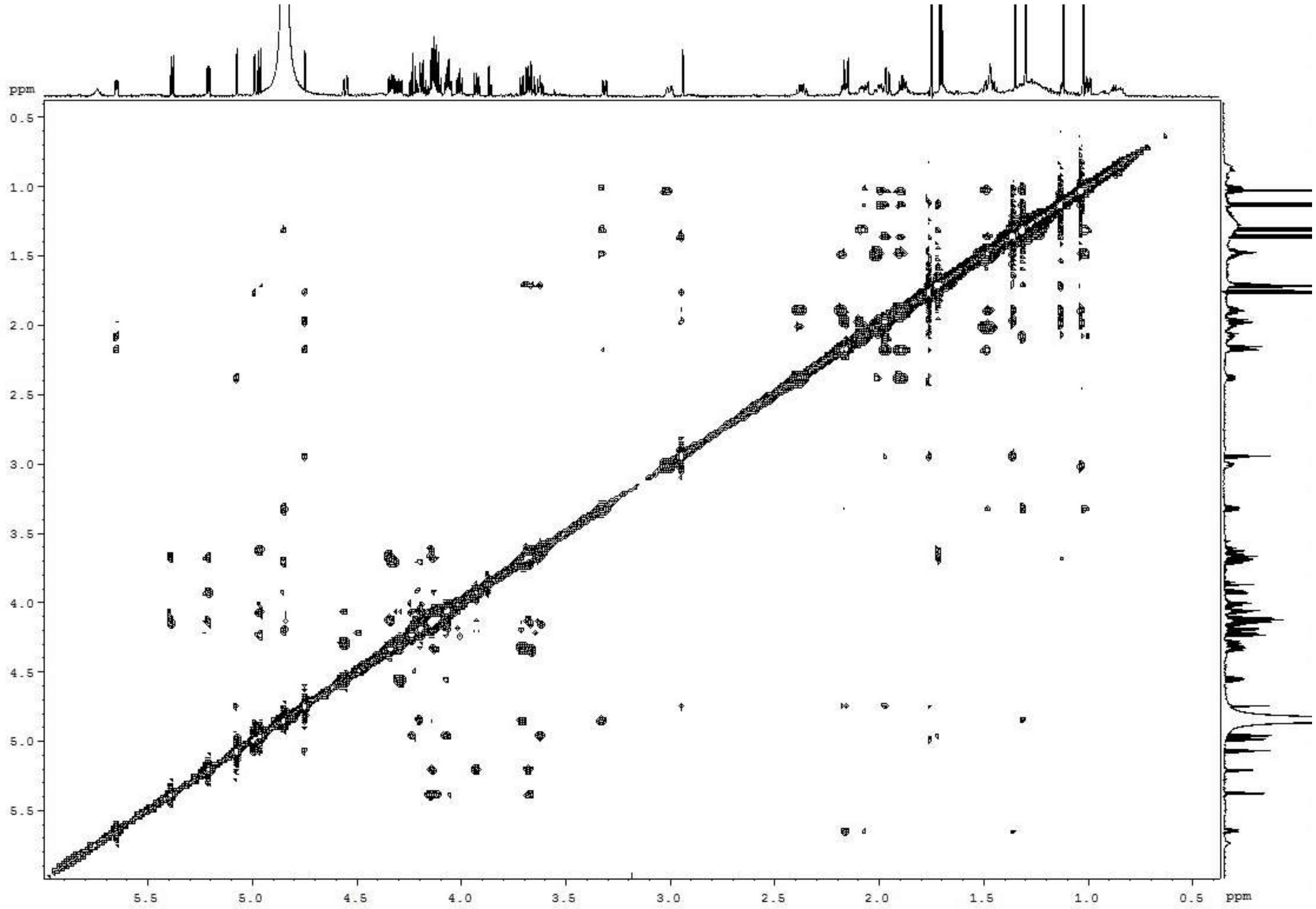

Figure S71. HRESIMS spectrum of pacificusoside K (**9**).

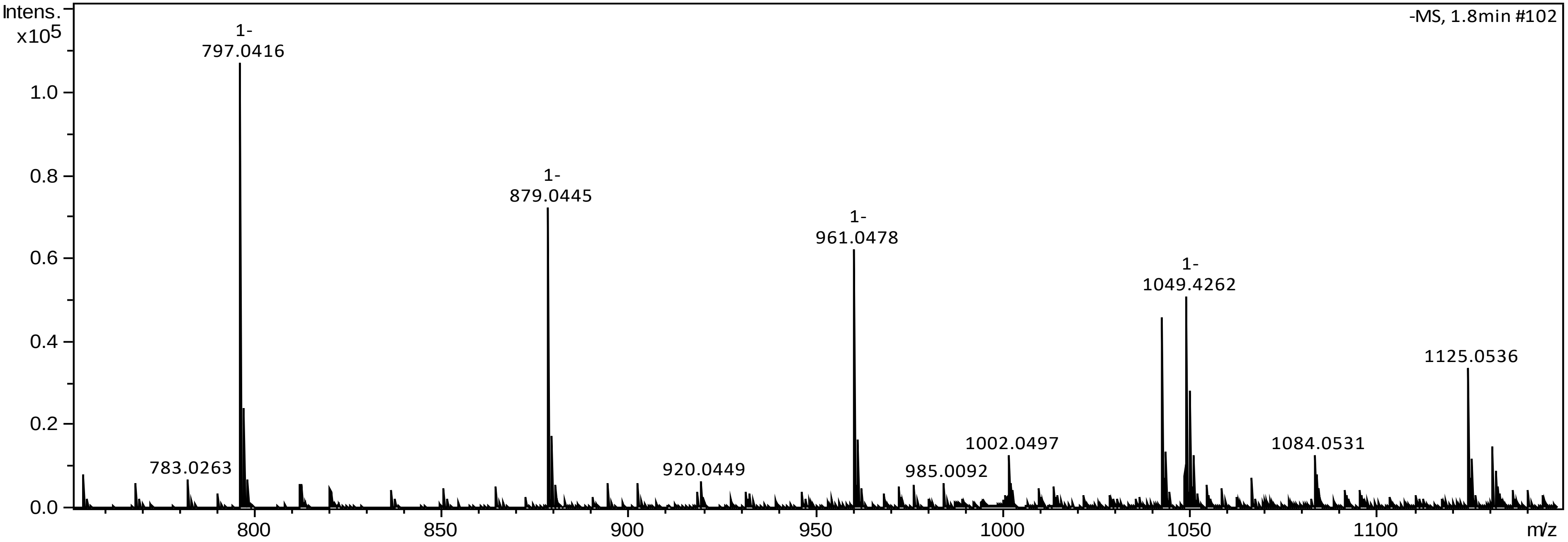

Figure S72. IR spectrum of pacificusoside K (**9**) in KBr.

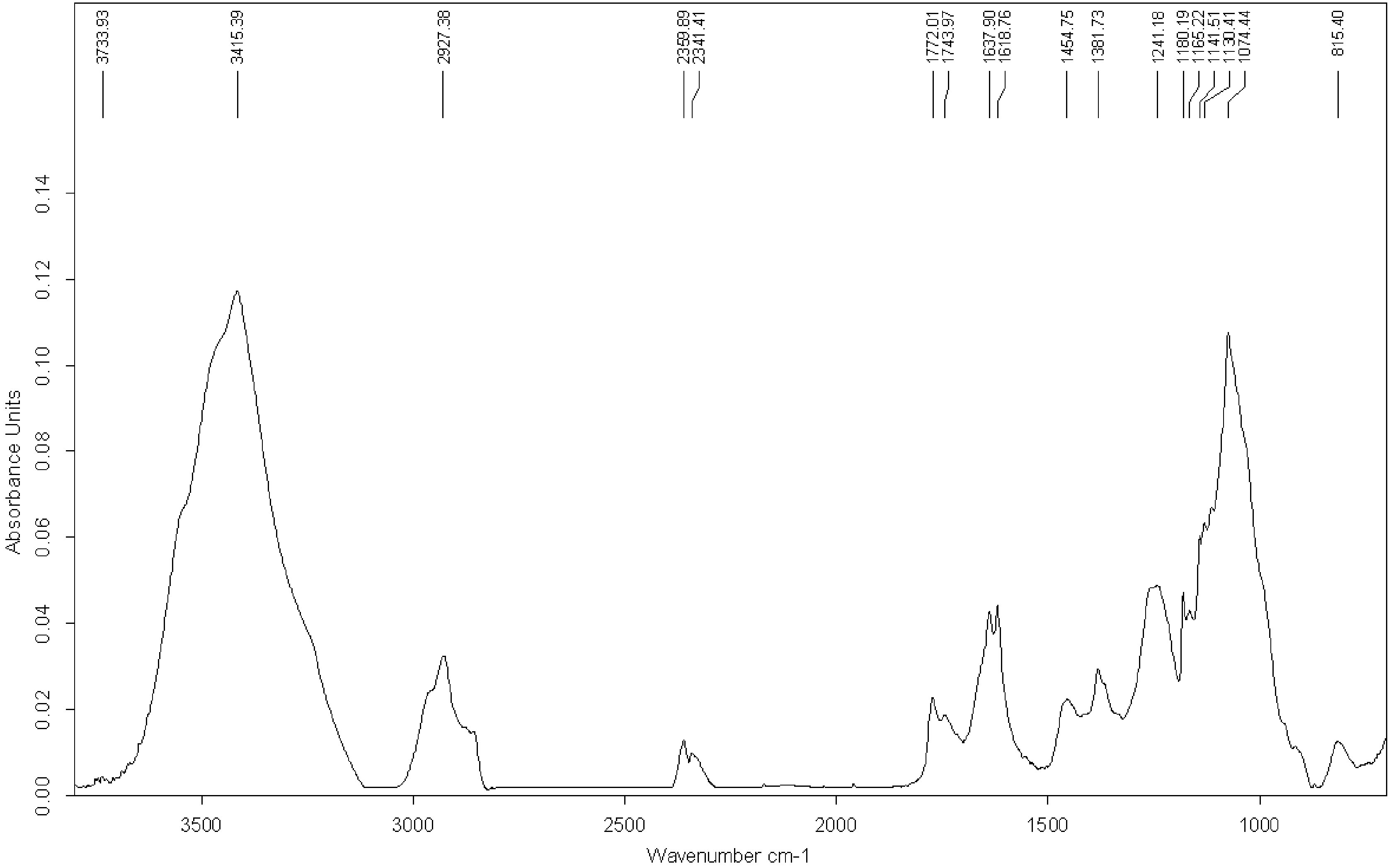

Figure S73. ^{1}H-NMR spectrum of pacificusoside K (**9**) in C_5D_5N.

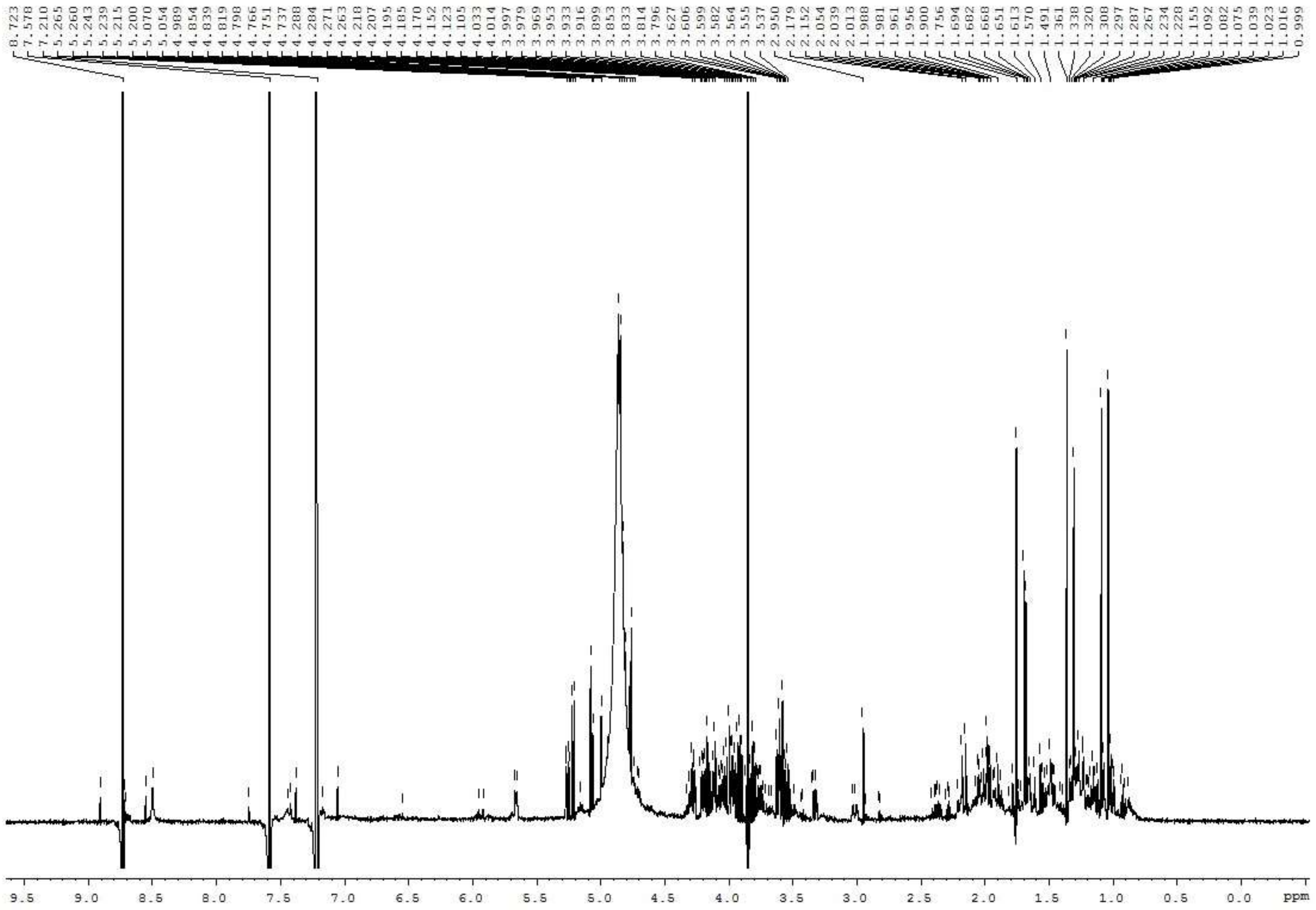

Figure S74. Expansion №1 of ^{1}H-NMR spectrum of pacificusoside K (**9**) in C_5D_5N.

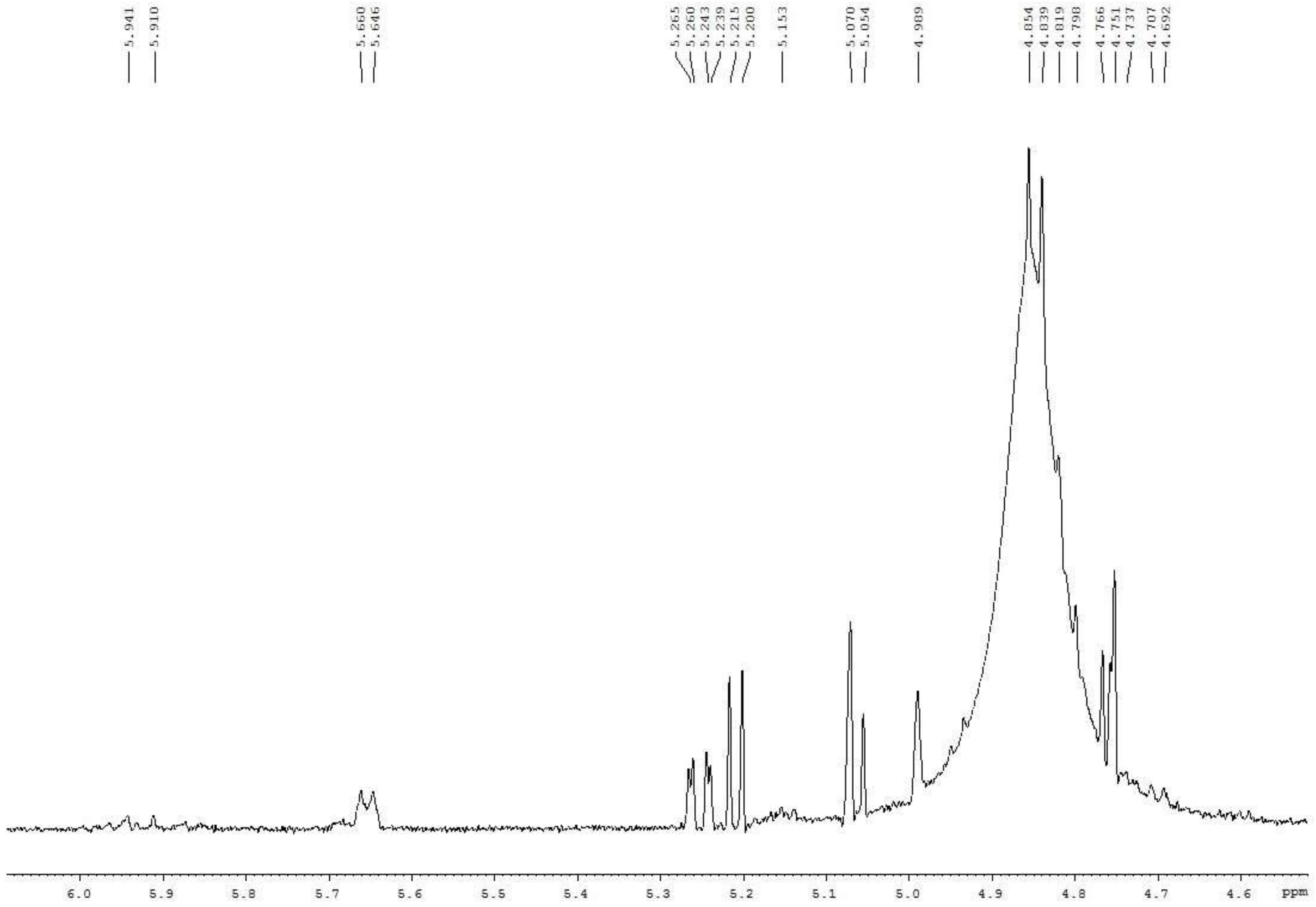

Figure S75. Expansion №2 of ^{1}H-NMR spectrum of pacificusoside K (**9**) in C_5D_5N.

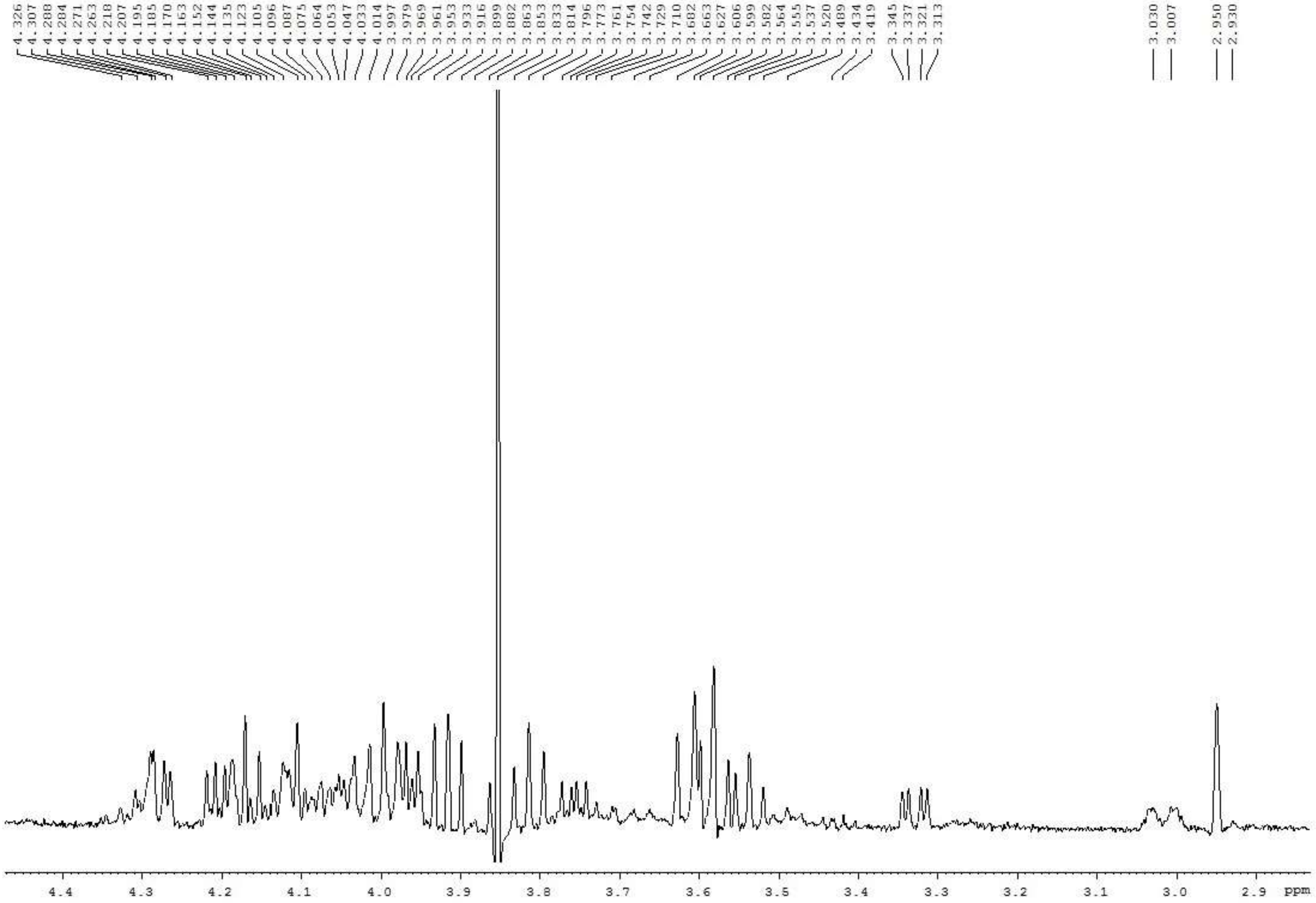

Figure S76. Expansion №3 of ^{1}H-NMR spectrum of pacificusoside K (**9**) in C_5D_5N.

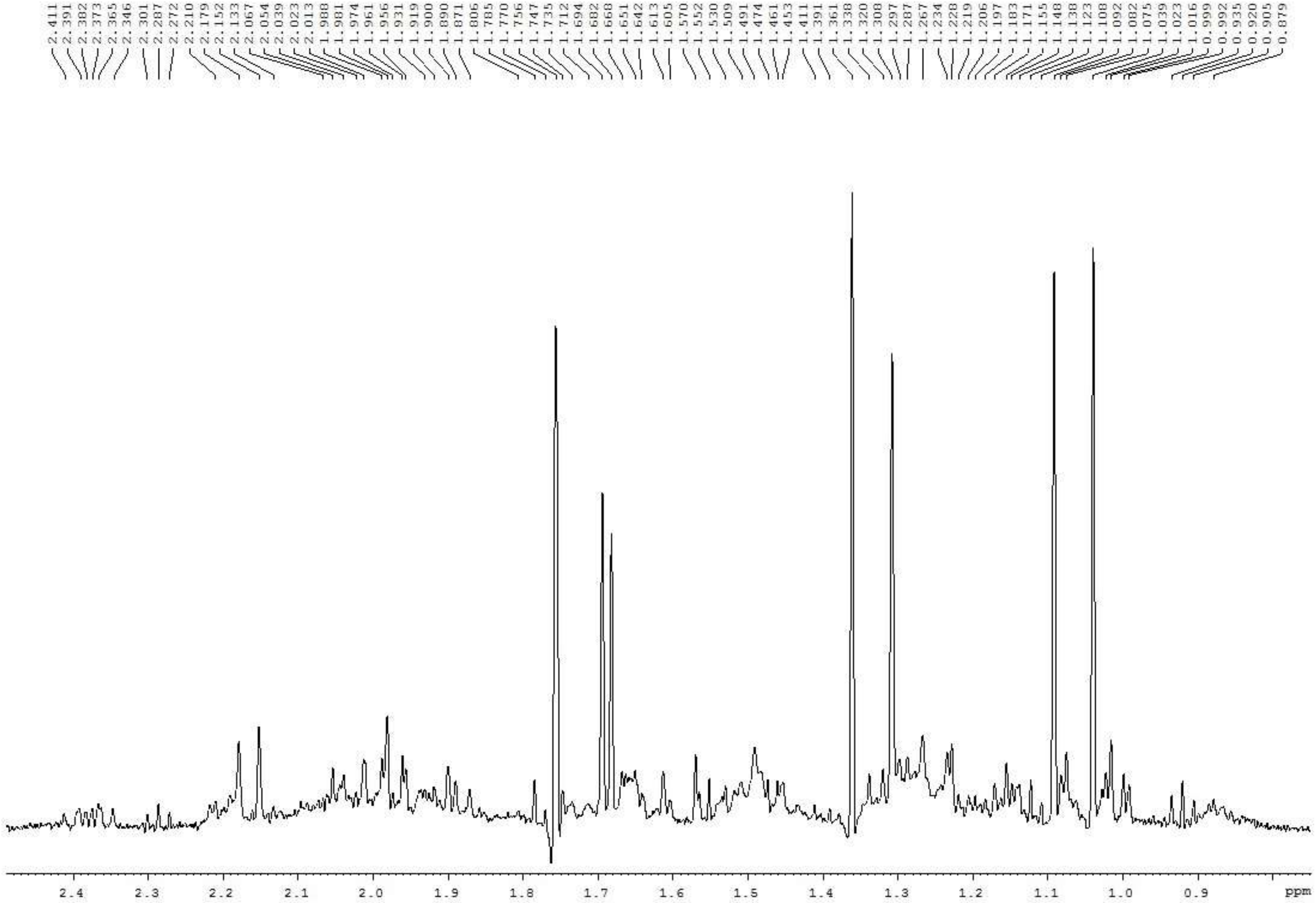

Figure S77. ^{13}C-NMR spectrum of pacificusoside K (**9**) in C_5D_5N.

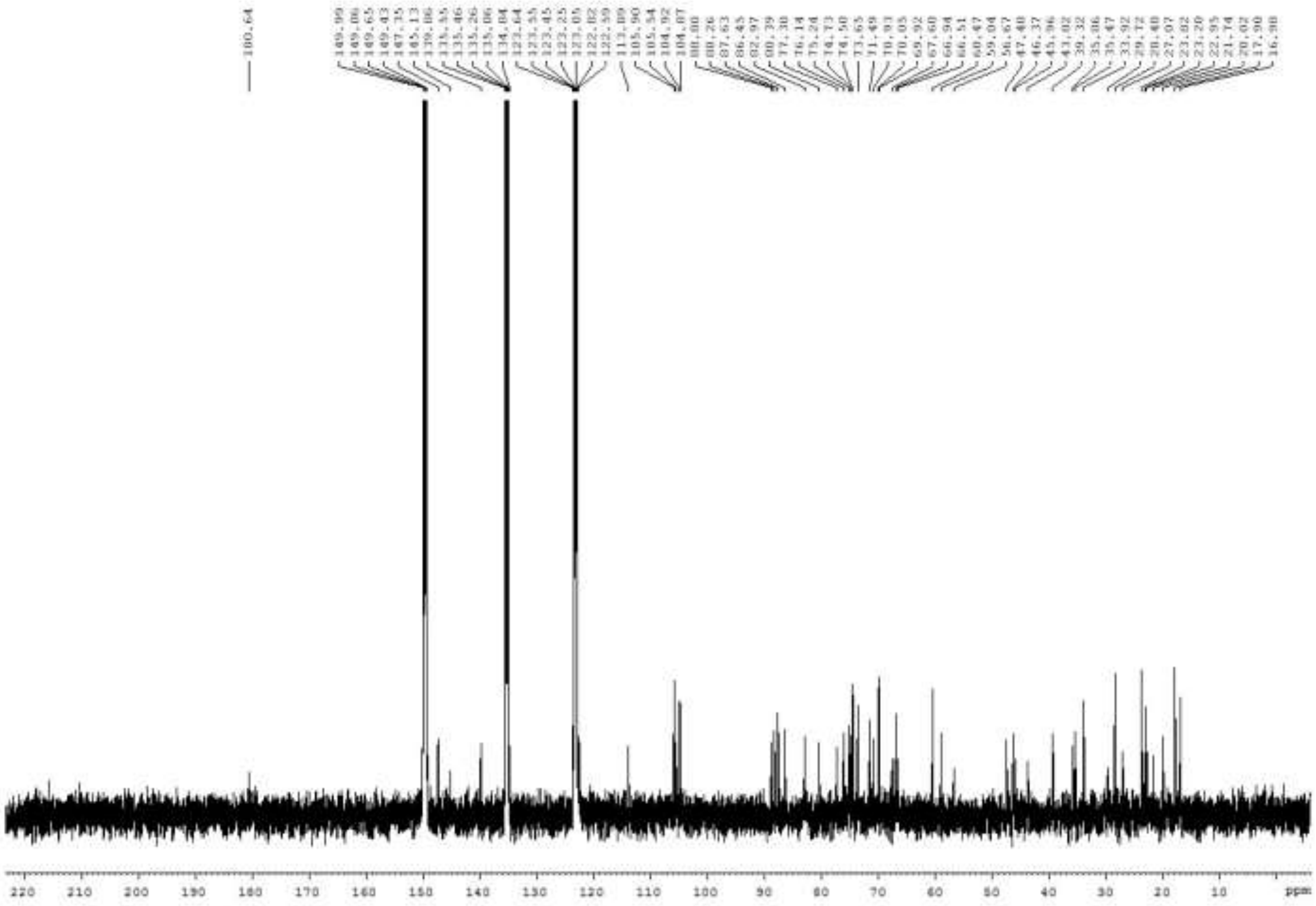

Figure S78. Expansion №1 of ^{13}C-NMR spectrum of pacificusoside K (**9**) in C_5D_5N.

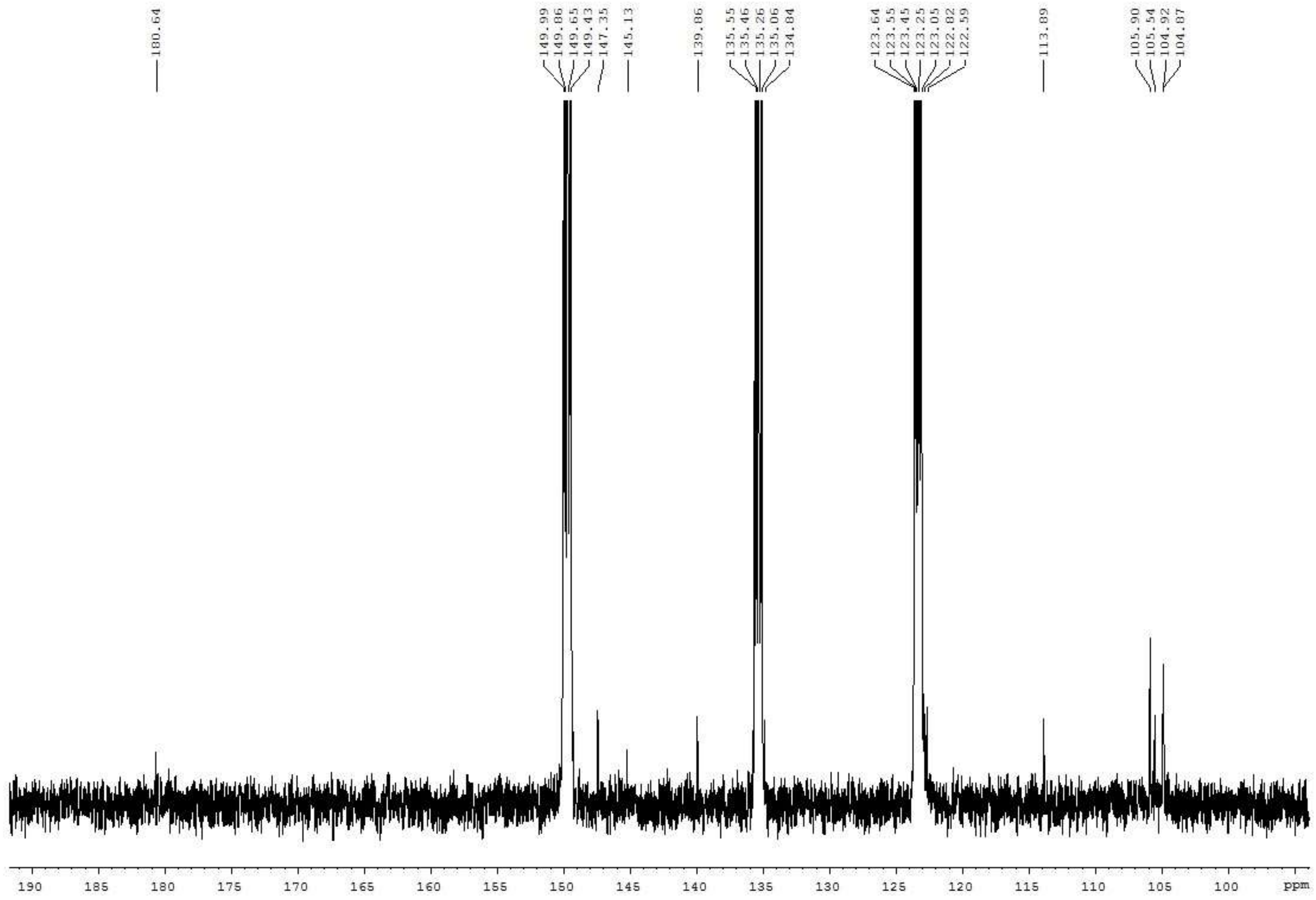

Figure S79. Expansion №2 of ^{13}C-NMR spectrum of pacificusoside K (**9**) in C_5D_5N.

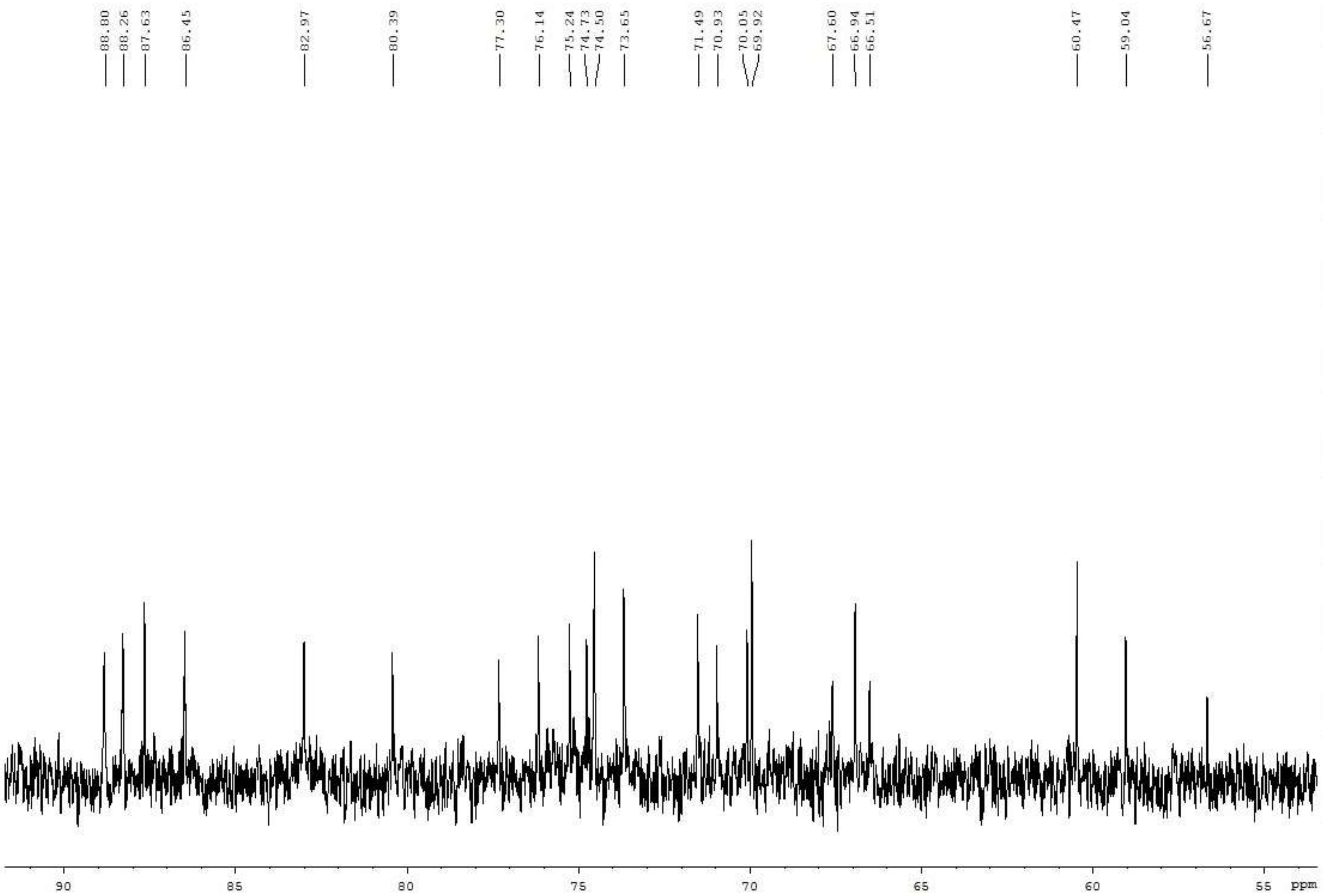

Figure S80. Expansion №3 of ^{13}C-NMR spectrum of pacificusoside K (**9**) in C_5D_5N.

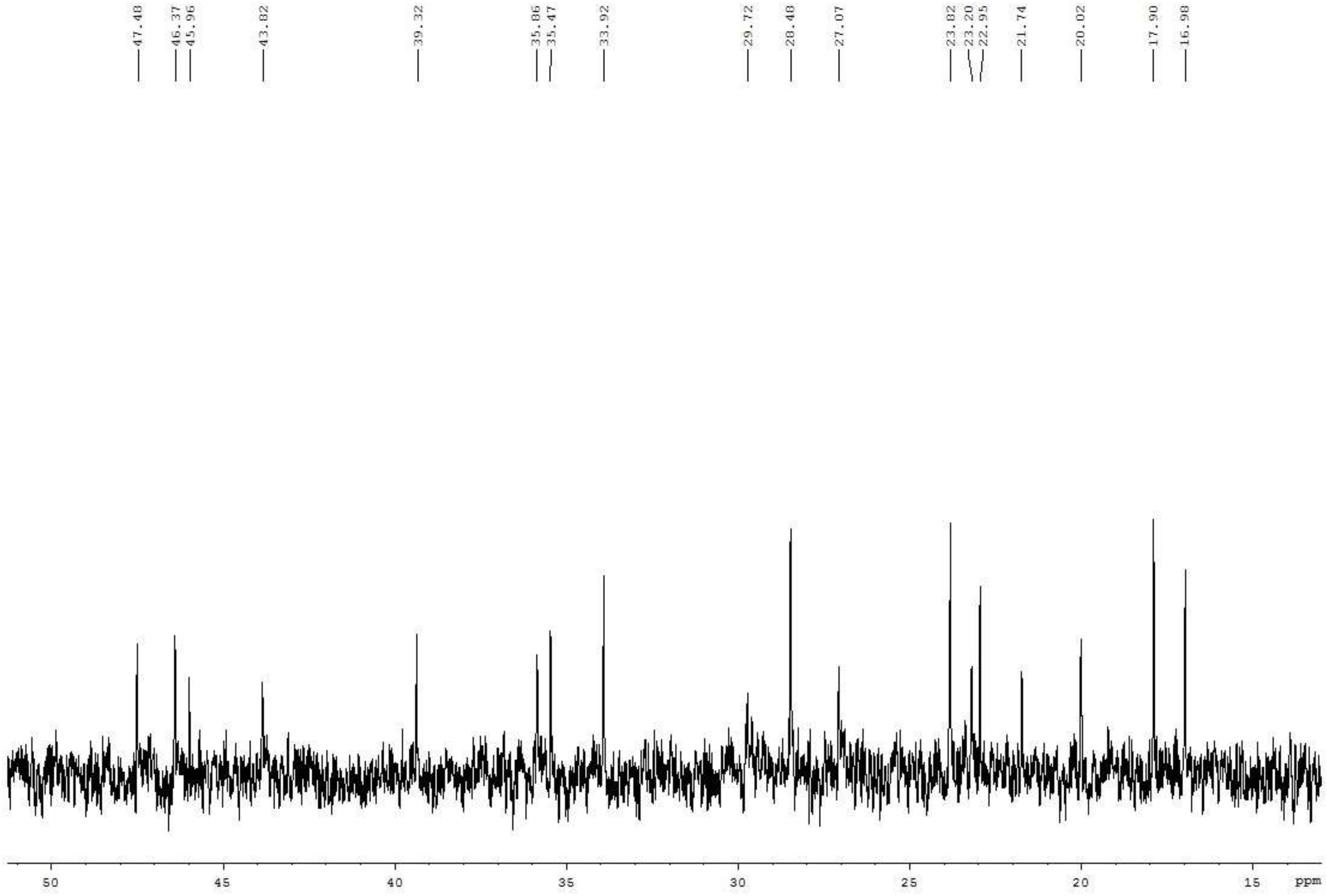

Figure S81. ^{1}H-^{1}H COSY spectrum of pacificusoside K (**9**) in C_5D_5N.

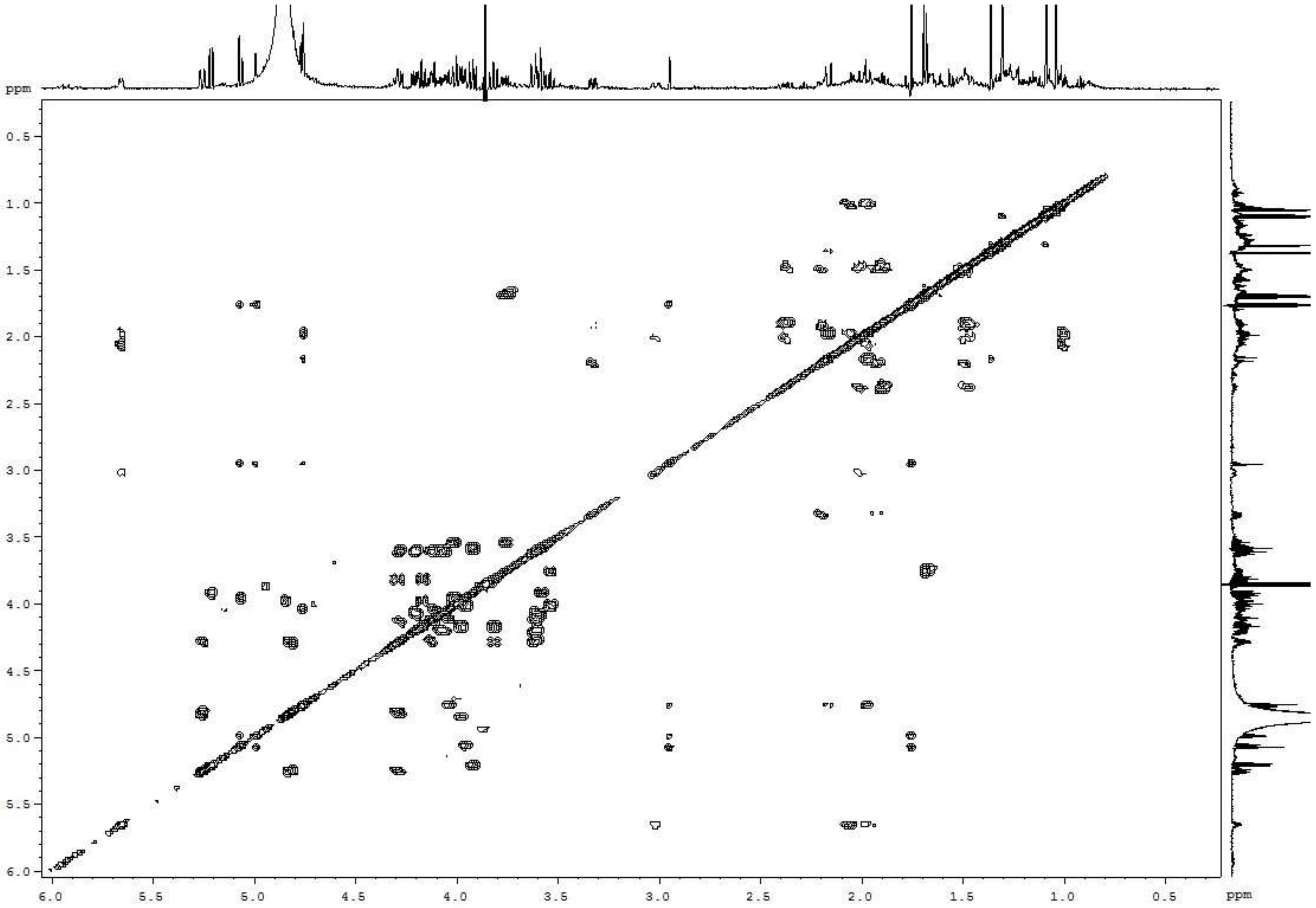

Figure S82. HSQC spectrum of pacificusoside K (**9**) in C_5D_5N.

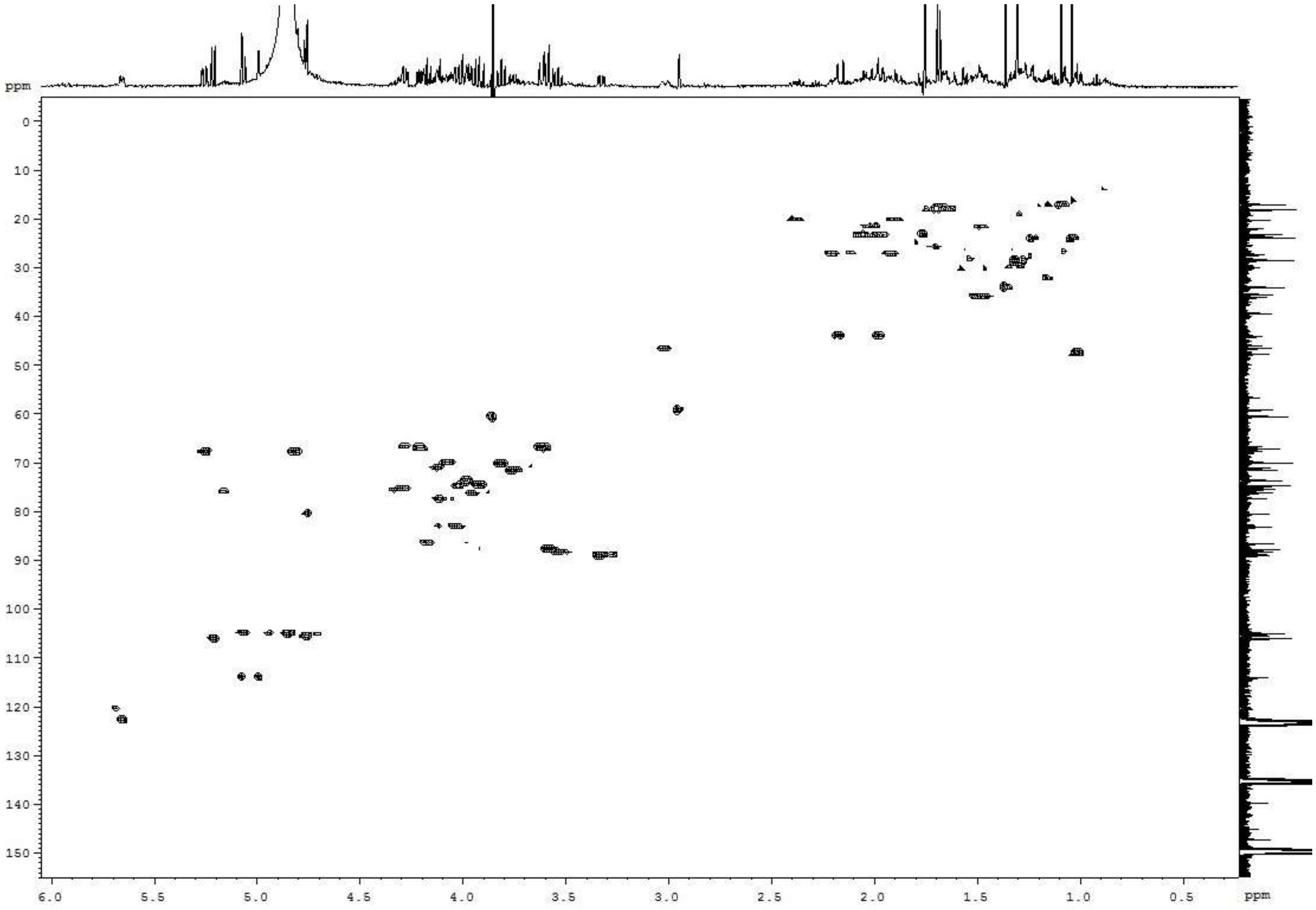

Figure S83. HMBC spectrum of pacificusoside K (**9**) in C_5D_5N.

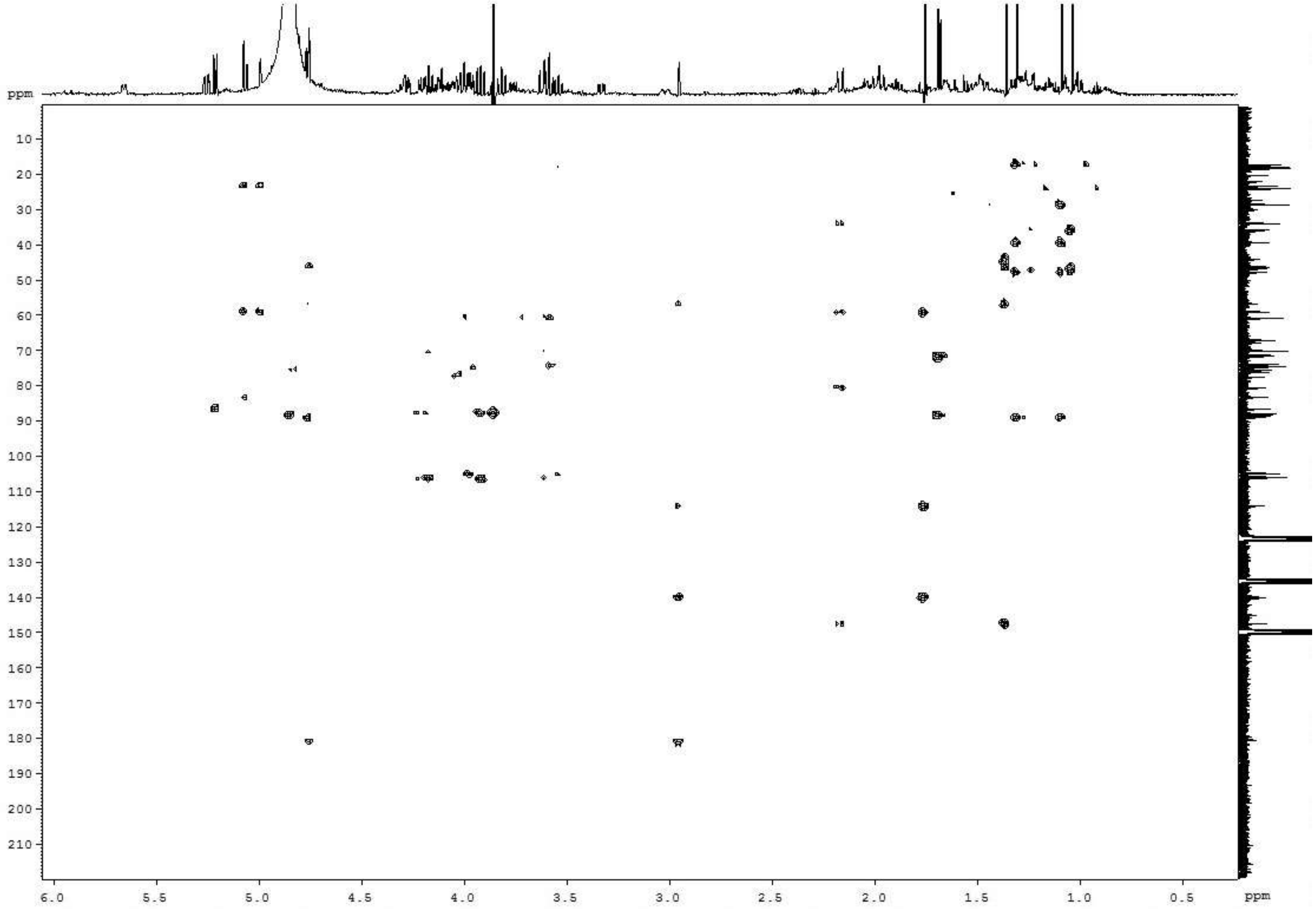

Figure S84. ROESY spectrum of pacificusoside K (**9**) in C_5D_5N.

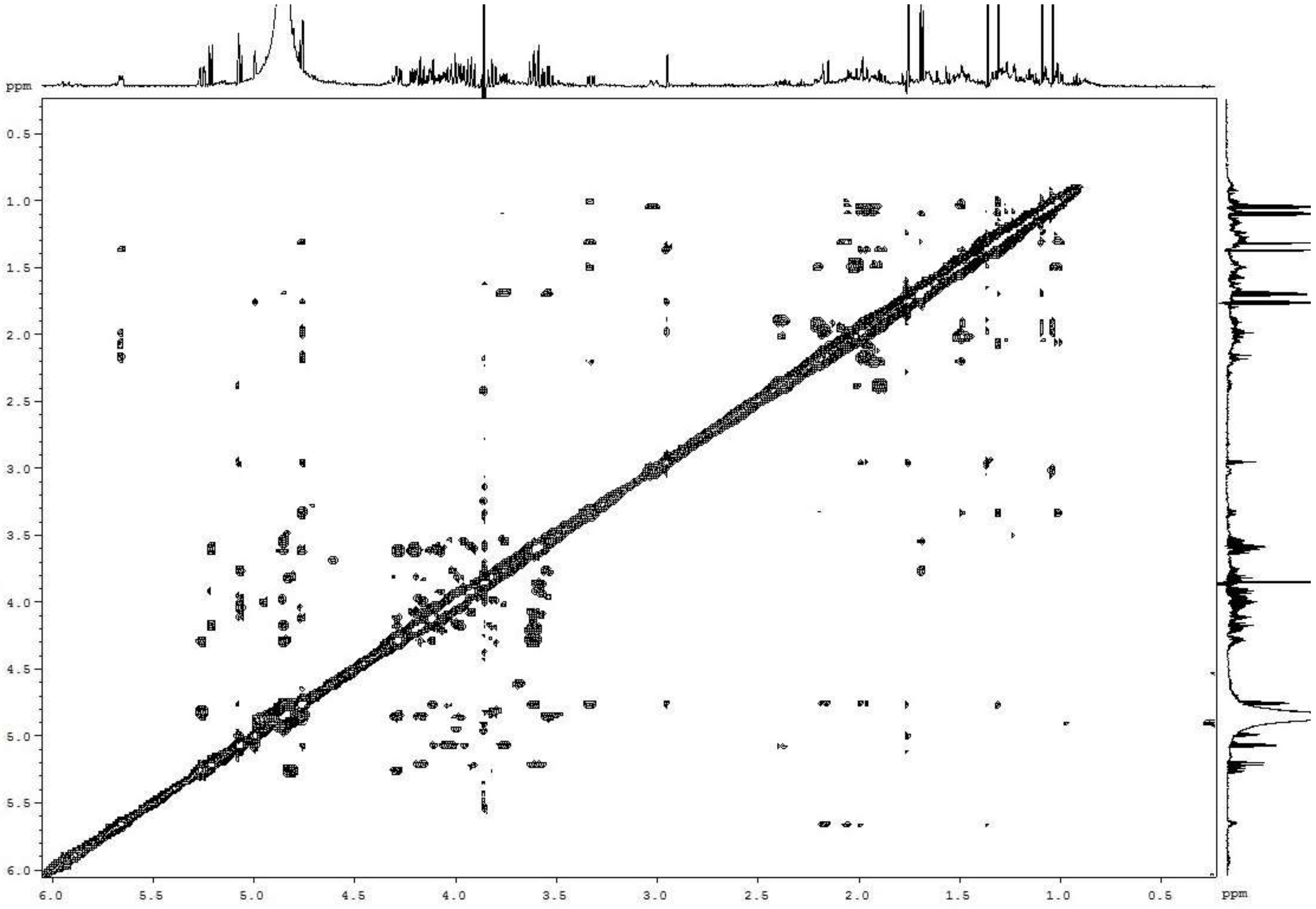

Supplement: Supplementary file 1 [file marinedrugs-20-00216-s001.zip › marinedrugs-1623363-supplementary.pdf]
